# Fine mapping of the major QTLs for biochemical variation of sulforaphane in broccoli florets using a DH population

**DOI:** 10.1038/s41598-021-88652-3

**Published:** 2021-04-26

**Authors:** Zhansheng Li, Yumei Liu, Suxia Yuan, Fengqing Han, Zhiyuan Fang, Limei Yang, Mu Zhuang, Yangyong Zhang, Honghao Lv, Yong Wang, Jialei Ji

**Affiliations:** Key Laboratory of Biology and Genetic Improvement of Horticultural Crops, Ministry of Agriculture, Institute of Vegetables and Flowers, Chinese Academy of Agricultural Sciences, Beijing, China

**Keywords:** Genetics, Agricultural genetics

## Abstract

Glucoraphanin is a major secondary metabolite found in *Brassicaceae* vegetables, especially broccoli, and its degradation product sulforaphane plays an essential role in anticancer. The fine mapping of sulforaphane metabolism quantitative trait loci (QTLs) in broccoli florets is necessary for future marker-assisted selection strategies. In this study, we utilized a doubled haploid population consisting of 176 lines derived from two inbred lines (86,101 and 90,196) with significant differences in sulforaphane content, coupled with extensive genotypic and phenotypic data from two independent environments. A linkage map consisting of 438 simple sequence repeats markers was constructed, covering a length of 1168.26 cM. A total of 18 QTLs for sulforaphane metabolism in broccoli florets were detected, 10 were detected in 2017, and the other 8 were detected in 2018. The LOD values of all QTLs ranged from 3.06 to 14.47, explaining 1.74–7.03% of the biochemical variation between two years. Finally, 6 QTLs (*qSF-C3-1*, *qSF-C3-2*, *qSF-C3-3*, *qSF-C3-5*, *qSF-C3-6* and *qSF-C7*) were stably detected in more than one environment, each accounting for 4.54–7.03% of the phenotypic variation explained (PVE) and a total of 30.88–34.86% of PVE. Our study provides new insights into sulforaphane metabolism in broccoli florets and marker-assisted selection breeding in *Brassica oleracea* crops.

## Introduction

Broccoli (*Brassica oleracea* var. *italica*), a member of Brassicaceae, is a popular vegetable that is rich in many nutrients, such as fiber, vitamin C, and proteins. Broccoli can reduce the risk of cancer and heart disease by decreasing cell damage, reducing inflammation, and protecting against chronic disease. Sulforaphane (SF) plays a key role in anticancer activities by inducing the Nrf-2 pathway and triggering the release of antioxidants and detoxifiers known as phase II enzymes^1^. SF is the second product of glucoraphanin (GRA), which is found in *Brassica oleracea* (*B. oleracea*) vegetables such as broccoli, kale (*Brassica oleracea* var. *acephala* f. *tricolor*), cabbage (*Brassica oleracea* var. *capitata*), Chinese kale (*Brassica oleracea* var. *alboglabra*), and kohlrabi (*Brassica oleracea* var. *caulorapa*), and is particularly abundant in broccoli^2,3^.

SF is the hydrolysis product of GRA and belongs to the aliphatic glucosinolates (GLS). When broccoli sprouts are consumed, GRA contained in vacuoles within the cytoplasm of plant cells is released and converted into SF via myrosinase (MY) located in the cytosol^4^. MYs are thioglucosidases (thioglucoside glucohydrolases, EC 3.2.1.147) that catalyze the initial step of the bioactivation of GLS^5^. MYs are usually composed of two identical 55–65 kDa polypeptides that are heavily glycosylated, resulting in a native molecular weight of the dimeric proteins of 120–150 kDa. MY have been characterized in *Arabidopsis thaliana* (*AtTGG1*-AtTGG6), *Brassica napus* (MA, MB and MC) and *B. oleracea* (broccoli and cabbage) and are classified into MY I (MA, MB, MC, AtTGG1-3) or MY II enzymes (AtTGG4 and 5 and others)^5–9^. Distinct patterns of expression suggest that the different MY enzymes may play different roles, such as showing differences in substrate specificity, since glucosinolate expression in the roots and above-ground tissue is different but overlapping in many species^10–12^. Most factors modifying glucosinolate hydrolysis affect either MY activity and specificity or the activity of the epithiospecifier protein (ESP), which is a very labile protein, in marked contrast to MY. Low concentrations of ascorbic acid and zinc ions can increase MY activity in broccoli and cabbage, while high concentrations of copper ions and magnesium ions decrease the yields of SF, but ferrous ions and ferric ions inhibit the formation of SF^9,13,14^. The amino acid sequence of broccoli MY has been elucidated (Acc. Nr.; MF461331), showing that the subunits have a molecular mass of 50–55 kDa, while the native molecular mass of MY is 157 kDa^15,16^. Therefore, the activity of broccoli MY in different organs can directly affect the recovery rate and yields of SF^17,18^.

GLS have been a topic of agricultural research for more than a century, which was initially often focused on adverse effects in animals fed concentrated crucifer-based feeds. However, there has recently been renewed interest in these compounds responsible for cancer prevention through the consumption of cruciferous vegetables. There are nearly 120 identified GLS from 16 families of angiosperms, including Brassicaceae, Capparaceae, Tovariaceae, and Caricaceae^19,20^. Interestingly, glucosinolate profiles vary widely between species and between varieties. The distribution of GLS frequently differs both qualitatively and quantitatively among plant parts (roots, leaves, sprouts, seeds, seedlings, etc.)^21–23^. GLS show great differences among different organs^20,22,24^. Therefore, the glucosinolate content depends not only on the genotype but also on the growing environment^2,25^. In previous reports, it has been indicated that glucosinolate content is mainly determined by genotype and the interaction between genotype and environment. Most of the known structural genes involved in glucosinolate metabolism have been identified and functionally characterized in *Arabidopsis thaliana*^26–28^. There are three stages in the biosynthesis of GLS: the side-chain elongation of amino acids, the development of the core structure, and secondary side-chain modifications. GLS are usually categorized into three classes based on the structure of their different amino acid precursors: aliphatic GLS, indole GLS, and aromatic GLS. The core pathway has been described on the basis of studies conducted mainly in *Arabidopsis*, and side-chain elongation and modification strongly influence the bioactivities of glucosinolate breakdown products^26^. The evolution and ecological relevance of glucosinolate variation were also reviewed in 2005^29^.

Broccoli is popularly reported to be high in SF, but different organs and tissues of broccoli show different levels of SF, which indicates the diversity of SF metabolism in different organs and developmental stages. To date, few reports have provided insight into the functional genes or loci related to explaining the differences in SF content in broccoli florets. One study revealed QTLs related to glucosinolate synthesis in *B. oleracea* plants based on a double haploid (DH) population derived from a cross between a DH rapid cycling line of Chinese kale and the DH ‘Early Big’ broccoli line^30^. There have been no reports of QTL mapping for SF metabolism in broccoli florets to date. Therefore, the mapping of QTLs responsible for the differences in SF metabolism in different environments will be helpful to better understand the relationships among the environment, MY activity and SF content in broccoli.

## Results

### Biochemical variation

**Figure 1:**
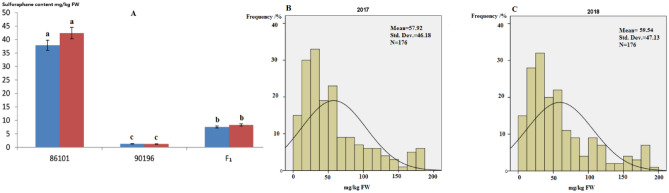
Biochemical variationof SF contents in the 86,101 and 90,196 lines and their hybrid (F_1_) (**A**). The lowercase letters indicate the significant differences in SF contents between the two parents and their hybrid F_1_ at *p* < 0.05. Frequency histogram of SF contents distributed in the DH population between 2017 (**B**) and 2018 (**C**).

Different SF contents were detected in the parental lines 86,101 (P_1_) and 90,196 (P_2_), and this difference was significant (1.37 mg/kg DW versus 37.49 mg/kg FW, respectively) (*p* < 0.05) (Fig. 1A). More importantly, there was a significant difference in the SF contents of the florets, and this population was suitable for constructing a permanent F_1_ DH population including 176 lines for the mapping of QTLs for SF metabolism in broccoli (Fig. 1).The DH family showed differences in the distribution in florets depending on genotype, and the coefficients of variation ranged from 0 to 0.20 and 0 to 0.14 in 2017 and 2018, respectively (Fig. 1B,C).

In our previous studies, analysis of variance (ANOVA) revealed significant differences (*p* < 0.01) in SF contents among DH plants in both 2017 (2.03 to 183.27 mg/kg FW) and 2018 (2.17 to 187.97 mg/kg FW), which indicated the existence of heritable variation. And meanwile, there existed segregation distortion and over-parent heterosis in the DH family, thus, the suitability of the DH population for genetic analysis. Significant variance of SF in the DH population was observed at *p* < 0.01 level. This result demonstrated that biochemical variation of SF contents was mainly under control of genetic factors and that climate might also play a role. Additionally, the Pearson correlation test was applied to the DH family, with a correlation coefficient ranging from 0.93 to 0.98 (*p* < 0.01). The data showed that there was a significant correlation between 2017 and 2018. The ranges and coefficients of variation (CV/%) among the DH population were greater than those in the P_1_ (1.20–1.66%), P_2_ (1.57–3.51%), and F_1_ (0.78–2.22%) populations, suggesting the existence of real variations in heredity and more genetic polymorphisms in the DH populations, which laid a good foundation for the subsequent genetic analysis.

The frequency distribution of SF contents in the DH population showed a continuous distribution and was difficult to group, suggesting that the SF content trait in broccoli is quantitative.

### Mixed major gene plus polygene inheritance analysis

A total of 38 specific models were obtained by IECM estimation in both two years, and three candidate models were selected by following the smaller AIC criterion (Table S1). According to fitness tests of *χ*^2^ uniformity test, Smirnov’s test, and Kolmogorov’s test, the G-1 model was seleted as optimal genetic model for SF contents in 2017 and 2018. In G-1 model, biochemical variation of sulforaphane was controlled by three major genes plus polygenes with addictive effect and epistatic effect. The genetic parameters of the optimal model were calculated using the least squares method (Table 1). In 2017 and 2018, the mean values of SF content were 57.91 mg/kg FW and 59.32 mg/kg FW, respectively. The additive effects of two pairs of major genes were 23.88 to 24.37 (*d*_*a*_), -7.16 to -7.02(*d*_*b*_), and 7.18 to 7.58 (*d*_*c*_) in both years. Correspondingly, the interaction effects of two major genes were -14.01 to -13.52 (*i*_*ab*_), 0.76 to 0.81(*i*_*ac*_), and -30.38 to -29.19(*i*_*bc*_), and the interaction effects of three major genes were -23.98 to -23.35 (*i*_*abc*_). The heritabilities of the major genes were 88.69% and 89.01% in 2017 and 2018, and the heritabilities of the major genes were and 20.45%, respectively. The mean value of the Hsi was 0.85. The additive effects of two pairs of major genes were 3.27% and 2.86%, respectively.

**Table 1:** Estimates of genetic parameters for SF contents in both years based on G-1 model.

Year	1st order genetic parameter								2nd order genetic parameter					
	*m*	*d*_a_	*d*_b_	*d*_c_	*i*_*ab*_	*i*_*ac*_	*i*_*bc*_	*i*_*abc*_	*σ*_*p*_^*2*^	*σ*_mg_^2^	*σ*_pg_^2^	*σ*_e_^2^	*h*_mg_^2^ (%)	*h*_pg_^2^ (%)
2017	57.91	24.37	− 7.16	7.18	− 13.52	0.81	− 30.38	− 23.98	2276.53	2037.49	58.79	184.99	88.69	3.27
2018	59.54	23.88	− 7.02	7.58	− 14.01	0.76	− 29.19	− 23.35	2268.32	2039.65	58.12	185.31	89.01	2.86

*m*, Mean; *d*_a_, *d*_b_ and *d*_c_, Additive effects of the first, second and third major genes; *i*_*ab*_, *i*_*ac*_, *i*_*bc*_, and *i*_*abc*_, The epistatic effect of additive × additive between two and three major genes; *σ*_p_^*2*^, Phenotypic variance; *σ*_pg_^2^, Polygenic variance; *σ*_mg_^2^, Major gene variance; *σ*_e_^2^, Environmental variance; *h*_mg_^2^(%), Major gene heritability; *h*_pg_^2^(%), Polygenic heritability.

### Linkage map construction

**Figure 2:**
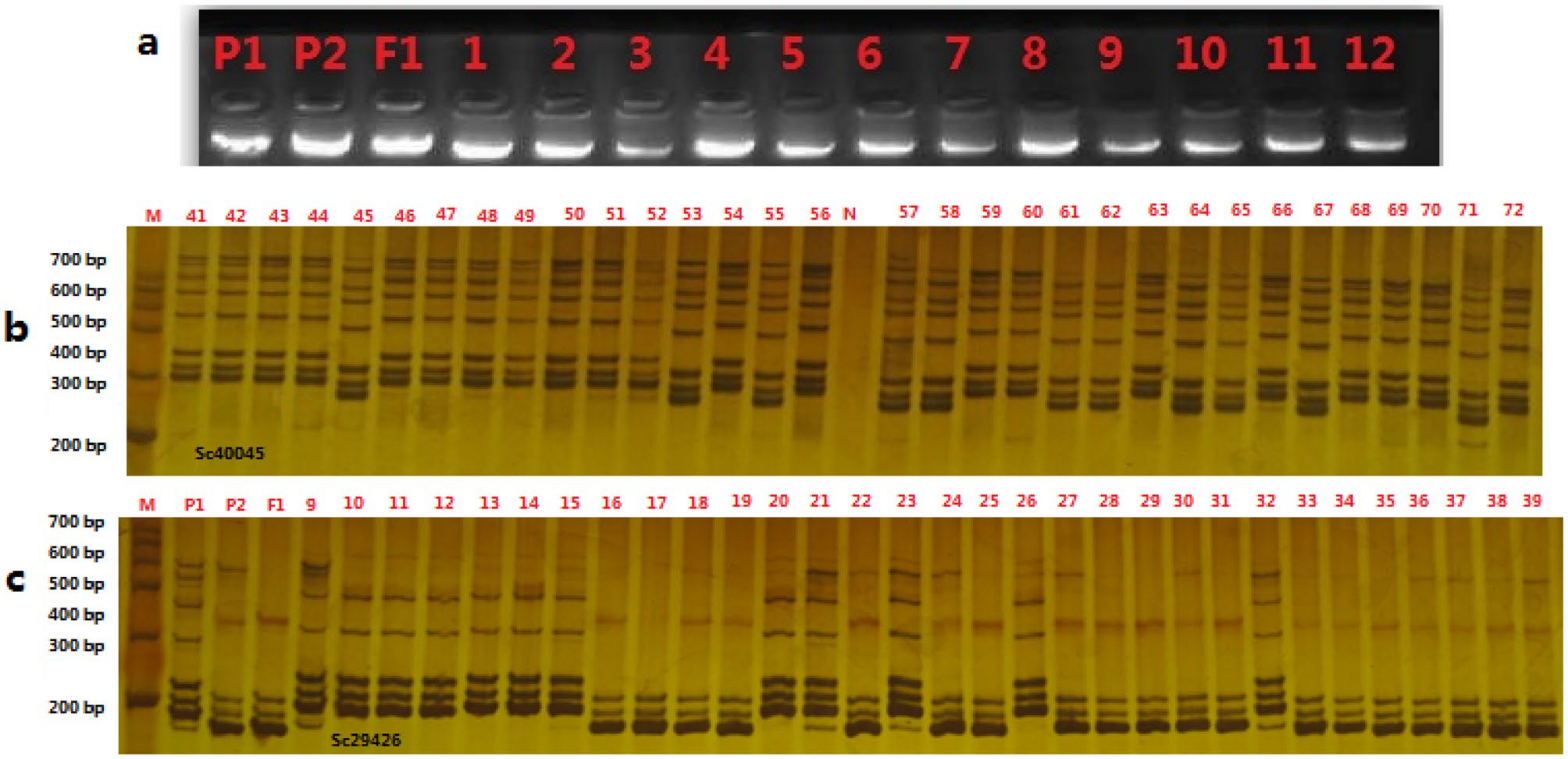
The electrophoretic profile of genome DNA (**a**) by agrose and SSR markers (Sc40045 and Sc29426) in experimental plants by polyacrylamide gelelectrophoresis (PAGE) (**b**,**c**).

The linkage map in this study consisted of 438 SSR markers on 9 chromosomes: it covered 1168.26 cM of the whole genome; and the average distance between each marker was 2.99 cM (Table 2). Table 2 showed that chromosome 3 exhibited the greatest number of SSR markers (169) and the longest length of the genetic map (430.52 cM). Chromosome 5 presented the lowest number of SSR markers (23), with a genetic map length of 221.3 cM. Chromosome 1 showed the lower number of SSR markers (29) and the shortest length of the genetic map (48.35 cM). The average physical distance between the mapped markers was calculated to be 0.85–2.30 Mb based on 603 Mb^31,32^. The electrophoretic profile of genome DNA (a) by agrose and SSR markers in experimental plants by polyacrylamide gelelectrophoresis (PAGE) was shown in Fig. 2.

**Table 2:** The profile of the linkage groups based on the broccoli DH population.

Chromosome	Length (cM)	Number of markers	Min (cM)	Max (cM)	Average (cM)
1	48.35	29	0	48.35	1.67
2	84.45	35	0	84.45	2.41
3	430.52	169	0	430.52	2.55
4	127.44	74	0	127.44	1.73
5	90.4	23	0	90.4	3.93
6	49.76	30	0	49.76	1.66
7	133.51	30	0	133.51	4.45
8	93.31	23	0	93.31	4.06
9	110.52	25	0	110.52	4.42

### Fine mapping of 6 major regions for SF metabolism

**Figure 3:**
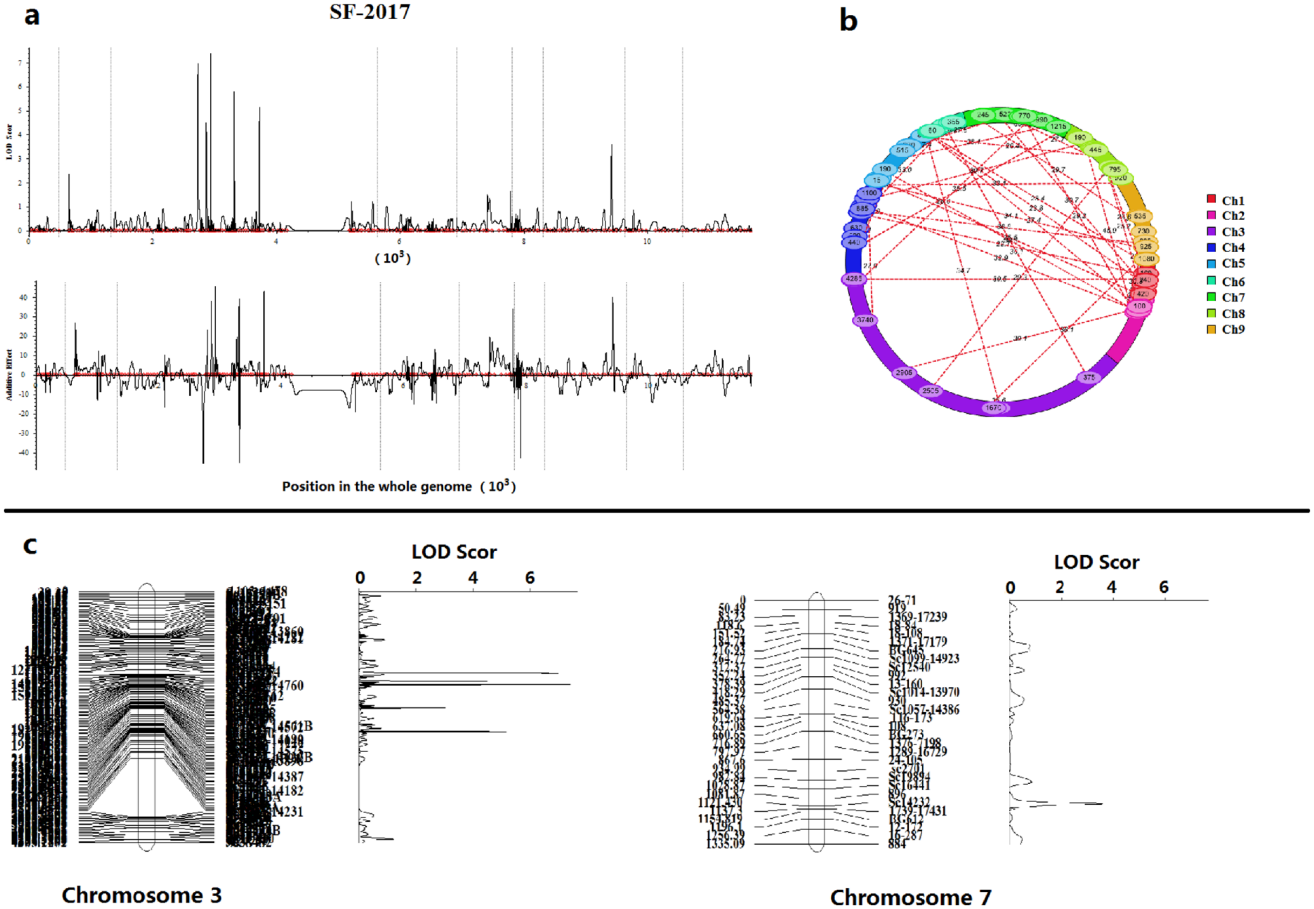
The profile of QTLs for SF content in broccoli florets based on 9 linkage maps (**a**), LOD values and additive effects of QTLs detected in 2017. The epistatic effects and QTL interaction effects with the environment (QE) at LOD = 3.0 (**b**). The major QTLs for biochemical variation of SF content were located on chromosomes 3 and 7 (**c**).

Using the biparental populations (BIP) module of inclusive composite interval mapping (ICIM) in QTL IciMapping 4.2 software for QTL analysis, a total of 18 QTLs for SF metabolism in broccoli florets were found during the two study years, which were mapped on chromosomes C2, C3, C5 and C7 (Table 3; Fig. 3a,b). The LOD values of all QTLs ranged from 3.06 to 14.47, explaining the biochemical variation of 1.74%-7.03% observed between the two years (Table 3). Among the 18 QTLs, 10 were detected in 2017 (*qSF-C2*, *qSF-C3-1*, *qSF-C3-2*, *qSF-C3-3*, *qSF-C3-4*, *qSF-C3-5*, *qSF-C3-6*, *qSF-C5-1*, *qSF-C5-2* and *qSF-C7*), and the other 8 were detected in 2018 (*qSF-C3-0*, *qSF-C3-1*, *qSF-C3-2*, *qSF-C3-3*, *qSF-C3-4*, *qSF-C3-5*, *qSF-C3-6* and *qSF-C7*) (Table 3). Furthermore, the results showed that 6 highly similar QTLs were stably detected in more than one environment, accounting for 4.54%-7.03% of the biochemical variation explained (PVE) with positive or negative additive effects (Add)^33,34^ (Table 3).

**Table 3:** Common QTLs in two independent genetic backgrounds.

Year	QTLs	Chromosome	Position	Left marker	Right marker	LOD	PVE (%)	Add
2017	*qSF-C2(* +*)*	2	16.6	13,450	8C0147	4.64	3.11	2.61
	*qSF-C3-1(* +)*	3	153.4	Sc1089-14,760	Sc3215	9.86	5.23	3.97
	*qSF-C3-2(* +)*	3	160.2	8C0226	Sc21910	14.47	5.76	4.41
	qSF-C3-3(-)*	3	198.6	sf50643	8C0445	11.85	6.68	− 4.62
	*qSF-C3-4(-)*	3	199.9	Sc1045-14,575	Sc8597	4.51	6.15	− 4.18
	*qSF-C3-5(* +)*	3	239.4	Sc2207	Sc4534	10.36	6.82	4.53
	*qSF-C3-6(* +)*	3	239.7	Sc4534	8C0828	12.25	7.03	4.59
	*qSF-C5-1(* +*)*	5	50.1	sc4037	sc25806	3.06	1.74	1.96
	*qSF-C5-2(* +*)*	5	87.9	sc7907	8C0211	3.34	4.61	3.42
	*qSF-C7(* +)*	7	111.6	896	Sc14232	10.93	6.56	4.79
2018	*qSF-C3-0(-)*	3	140.2	1165–1544	Sc38233	6.92	7.01	− 4.65
	*qSF-C3-1(* +)*	3	153.5	Sc1089-14,760	Sc3215	4.47	4.69	3.85
	*qSF-C3-2(* +)*	3	160.1	8C0226	Sc21910	7.38	6.18	4.64
	qSF-C3-3(-)*	3	198.7	sf50643	8C0445	5.73	6.51	− 4.62
	*qSF-C3-4(* +*)*	3	199.4	8C0227	Sc1045-14,575	4.06	5.52	3.96
	*qSF-C3-5(* +)*	3	239.2	Sc2207	Sc4534	4.57	6.35	4.34
	*qSF-C3-6(* +)*	3	239.5	Sc4534	8C0828	5.15	6.59	4.40
	*qSF-C7(* +)*	7	111.1	896	Sc14232	3.58	4.54	3.87

( +) Represents a positive additive effect, indicating that the alleles at the locus increased the content of SF; (-) represents a negative additive effect, indicating that the alleles at the locus decreased the content of SF; PVE represents the phenotypic variance explained; Add represents the additive effect; *(in red) indicates an overlapping chromosomal region detected in the two years and considered to be the same locus.

According to comparisons between two environments, 6 QTLs for SF metabolism in broccoli florets were considered to be significant QTLs, which were located on chromosomes 3 and 7 (Fig. 3c). Among these QTLs, 5 (*qSF-C3-1*, *qSF-C3-2*, *qSF-C3-3*, *qSF-C3-5*, and *qSF-C3-6*) were distributed on chromosome 3, and 1 (*qSF-C7*) was distributed on chromosome 7. Moreover, these significant QTLs showed a higher PVE and additive effects (Table 3). The *qSF-C3-1*, *qSF-C3-2*, *qSF-C3-3*, *qSF-C3-5*, and *qSF-C3-6* QTLs explained 4.69%-5.23%, 4.69%-5.76%, 6.51%-6.68%, 6.35%-6.82%, and 6.56%-7.03% of the observed biochemical variation, respectively, including one QTL with a negative additive effect (*qSF-C3-3*). The *qSF-C7* QTL, found on chromosome 7, explained 6.56%-6.59% of the observed biochemical variation, with a positive additive effect (3.87–4.79). In addition, 6 important QTLs, *qSF-C3-1*, *qSF-C3-2*, *qSF-C3-3*, *qSF-C3-5*, *qSF-C3-6*, and *qSF-C7,* were stably detected with the same flanking markers corresponding to the left markers and right markers of Sc1089-14,760 and Sc3215, 8C0226 and Sc21910, sf50643 and 8C0445, Sc2207 and Sc4534, Sc4534 and 8C0828, and 896 and Sc14232, respectively (Table 3).

## Discussion

A variety of genetic and environmental factors ultimately affect the metabolite levels of glucosinolate in *Brassica* crops, such as *Brassica napus*^35,36^, *Brassica rapa*^37^, and *Brassica juncea*^38,39^. It has been reported that this conclusion is also applicable the GRA and SF in broccoli. Moreover, different organs of broccoli, including the seeds, seedlings, sprouts, leaves, and stalks, present quite different SF contents, which are mostly determined by the genotype and its interactions with the environment^22,40–44^. Similarly, in the florets and leaves that we analyzed, it was shown that there were significant variations in SF accumulation in different genotypes and organs at different developmental stages (florets at mature, buds to flowers at bolting)^3,22^. In addition, it has been proved that glucoraphanin content can be regulated and affected by several genes, such as *BCAT4* (branched-chain aminotransferase 4), *MAM1* (methylthioalkylmalate synthase 1), *CYP79F1* (dihomomethionine N-hydroxylase), *AOP2* (2-oxoglutarate-dependent dioxygenases)^45–48^. In our study, genetic analysis of SF contents in broccoli florets was firstly estimated by a DH population in both years, and the result revealed that there might be at least three major genes controlling the biochemical variation of SF contents, at the same time, the environment was also an influence factor. So our result provided a direct evidence in SF or GRA metabolism in crucifer plants, which was consistent with most previous reports. Therefore, according to previous reports, broccoli shows considerable differences in SF contents in different organs and developmental stages, suggesting that SF metabolism is regulated by different genes than glucosinolate biosynthesis. To date, most QTL mapping studies of glucosinolate have focused on *Arabidopsis*, *Brassica napus* and *Brassica rapa* crops, and few reports have provided QTL information on SF in broccoli florets based on a permanent DH population derived from broccoli varieties to study the underlying regulatory mechanism. Therefore, the mapping of QTLs responsible for the differences in SF metabolism in different environments is helpful to better understand the relationships among the environment, MY activity and SF content in broccoli^18^.

**Figure 4:**
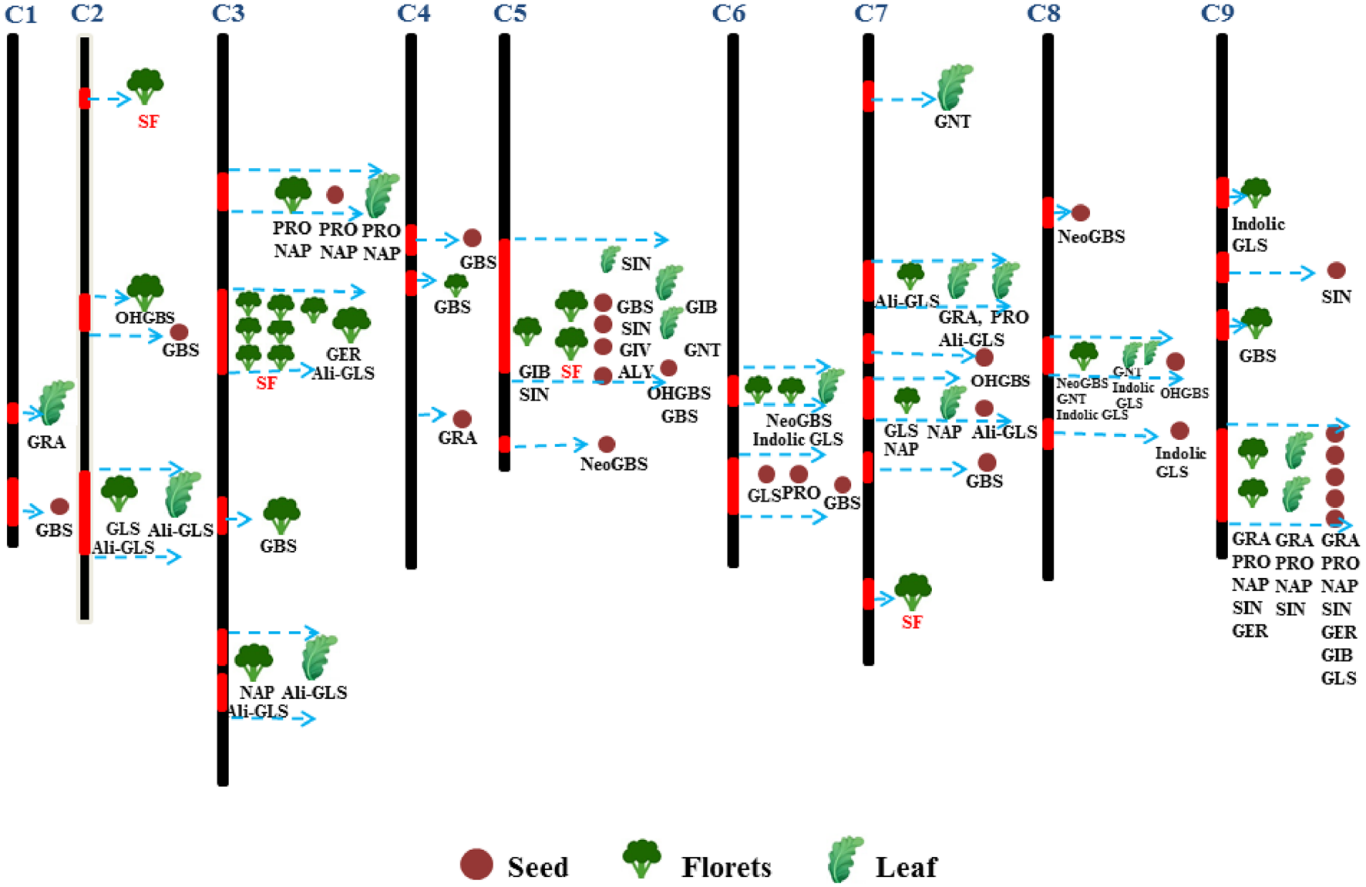
Framework map of broccoli populations showing the metabolic QTLs for SF, total GLS, and individual GLSs, including aliphatic GLS (Ali-GLS): glucoraphanin (GRA), gluconapin (NAP), glucobrassicanapin (GBN), glucoerucin (GER), progoitrin (PRO), sinigrin (SIN), glucoiberverin (GIV), glucoiberin (GIB), and glucoalyssin (ALY); indolic GLS (indolic GLS): glucobrassicin (GBS), neoglucobrassicin (neoGBS), and 4­hydroxyglucobrassicin (OHGBS); and an aromatic GLS (Aro-GLS): gluconasturtiin (GST). The chromosome bars were mapped based on the *B. oleracea* genome, previous reports and our research^30,31,49^. QTLs are represented by different shapes and colors depending on different plant organs: seeds (auburn), florets (dark green), and leaves (green). The numbers and regions of their coverage in the seeds, florets and leaf indicate the genetic loci and alleles. The column cabel (red) in the chromosomes shows the QTLs regeion for individual GLSs, and dotted arrow (light blue) points to the single or many traits. Different colors of QTLs for GLS and SF were used to distinguish between previous studies (in dark) and this work (in red).

SF plays an important role in anticancer effects and the prevention of cerebrovascular disease. Most people obtain nutrition from broccoli by consuming the florets or their extracts, so this study focused on the investigation of QTLs for SF metabolism in broccoli florets by using a permanent DH population including 176 individuals. In our study, significant QTLs for SF metabolism in broccoli florets were mapped to chromosomes 3 and 7. In previous studies, QTLs for total GLS, aliphatic GLS, GRA, progoitrin (PRO), gluconapin (NAP), glucoerucin (GER), glucobrassicin (GBS), and 4-hydroxyglucobrassicin (OHGBS) were found on chromosomes 3 and 7 (Fig. 4)^30,49,50^. GRA is the precursor of SF, whose production is catalyzed by MY, and it belongs to the aliphatic GLS; therefore, we emphasized the mapping of GRA, aliphatic GLS and MY. In previous reports, we found that some QTLs for aliphatic GLS and GRA were located on chromosomes 1, 2, 3, 4, 7 and 9; QTLs for both aliphatic GSL and GRA were located on chromosome 7; and QTLs for GRA alone were located on chromosomes 1, 7 and 9 (Fig. 4). In our study, 18 QTLs for SF were detected on chromosomes 2, 3, 5 and 7, and 6 significant QTLs were mapped to chromosomes 3 and 7, which indicated that some QTLs identified in this study were consistent with those identified in previous reports, but our results showed more QTLs on chromosome 3, suggesting shorter genetic distance and that more detailed information will need to be obtained in future research. Therefore, to a large extent, the important QTLs for SF metabolism in broccoli florets might be located on chromosome 7. In fact, SF metabolism is determined by polygenic regulation, and major genes and microgenes both play important roles in different organs, developmental stages and environments^13,17,20,26,29,30,41,43^.

In the two years, 6 common QTLs (*qSF-C3-1*, *qSF-C3-2*, *qSF-C3-3*, *qSF-C3-5*, *qSF-C3-6* and *qSF-C7*) were stably detected with the same flanking markers. Considering to the similar contorl of two envirnmonts, except for the slight changes in temperature, the results might provide a reliable experiment basis for studying molecular mechanism of SF regulation. At present, the research on QTL mapping for SF metabolism in broccoli is limited and is not sufficiently deep. On the basis of this study and several previous reports, we can infer that the *qSF-C3-1*, *qSF-C3-2*, *qSF-C3-5*, *qSF-C3-6* and *qSF-C7* QTLs play an important role in SF accumulation as upstream regulated genes with positive effects. The *qSF-C3-3* QTL might be a negatively regulated gene in the SF synthesis pathway and could be an ESP-related gene or a secondary product-regulated gene for substrates competing with GRA. In addition to 6 common QTLs, 5 other QTLs (*qSF-C2*, *qSF-C3-0*, *qSF-C3-4*, *qSF-C5-1* and *qSF-C5-2*) were detected on chromosomes 2, 3 and 5. In previous reports, QTLs for aliphatic and total GLS have been found on chromosomes 2 and 3. In the present study, we also found QTLs (*qSF-C5-1* and *qSF-C5-2*) on chromosome 5 that have not been reported previously.

It has been reported that the AOP family plays an important role in the side chain modification of GLS. The function of the *AOP2* gene is absent in broccoli, and *AOP3* is associated with the apparent regulatory control of aliphatic GLS accumulation by catalyzing the production of hydroxyalkyl glucosinolate from methylsulfinylalkyl glucosinolate with C3 side chains, but the specific roles of *AOP1* and *AOP3* in controlling aliphatic GLS accumulation are less well known^26,51–54^. In our study, several QTLs (*qSF-C3-0*, *qSF-C3-4*, *qSF-C5-1* and *qSF-C5-2*) detected as special regions in broccoli might be related to the AOP family, which requires further research.

We detected some major and special QTLs for SF metabolism in broccoli florets based on a DH population in various environments. It is believed that these QTLs can be used for marker-assisted selection breeding and fine mapping.

## Methods

### Plant materials

A DH of broccoli was developed from F_1_ plants resulting from the cross of parents from inbred lines 86,101 and 90,196, and there were 176 plants (genotypes) in this DH family generated from F_1_ cultivated by a pollination method as our previously described^55^. Actually, DH is normally used to retain the desired alleles in the genome and a quicker way to produce homogenous line, instead of self-pollination over generations to produce inbred lines. In this study, all plant materials were bred and planted at the same farm of the Institute of Vegetables and Flowers (39°90′N, 116°29′E), Chinese Academy of Agricultural Sciences (Beijing) (IVF-CAAS). These 176 DH lines, their parents (individual 30 plants), and the F_1_ (30 plants) were all grown in autumn 2017 (environment 1) and 2018 (environment 2) in Beijing (IVF-CAAS), separately. All plants were sown on July 6–8 in 2017 and 2018, and were planted in the field after one month. The two environments included 266 plants with three repeats (n = 3, total 798 plants) at random, and the plants were spaced 30 cm × 50 cm apart with 15 plants in each row. For the DH population, the experimental plots were surrounded by two additional rows planted to serve as a protective buffer. There were similar control and management for environment 1 and environment 2, the difference was that the monthly average temperature in september and october 2018 was 18.6 °C to 35 °C and 10.2 °C to 21.6 °C, which were a little higher than corresponding month in 2017 (17.2 to 32.3 °C and 8.1 °C to 19.2 °C).

**Figure 5:**
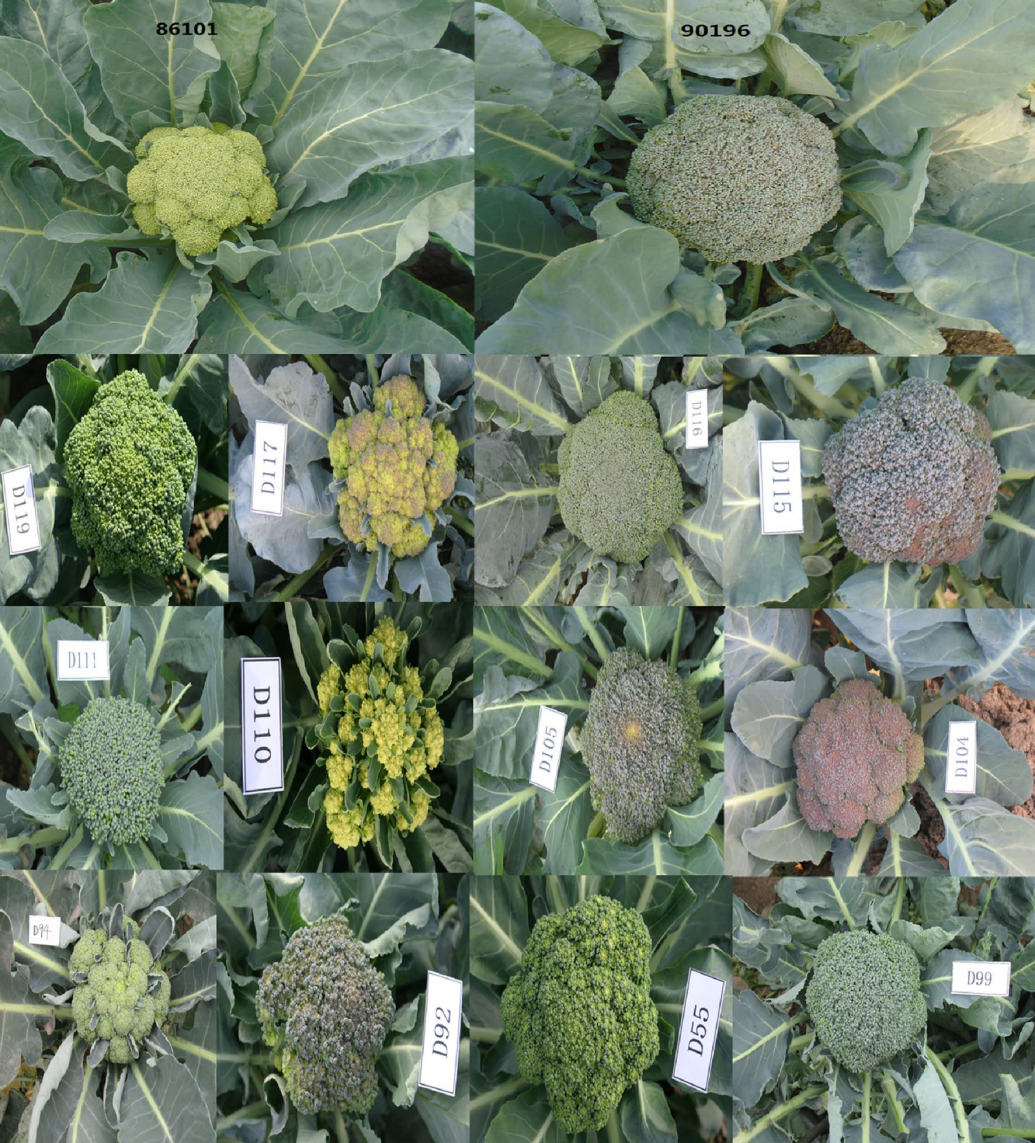
Phenotypic traits of the parental lines 86,101 and 90,196 and some individual DH lines.

Line 86,101 showed very early maturity (55 days after planting in the field) and exhibited some clovers in its small florets a yellow-green broccoli head color (Fig. 5). Inbred line 90,196 also showed early maturity (60 days after planting in the field) but exhibited no clovers in its middle florets, and its broccoli head color was green and changed to deep purple under freezing temperature. The DH family presented differences in the phenotypes of traits such as head color, shape, size, and the presence of clovers (Fig. 5).

### Pretreatment and genetic analysis of SF

When the broccoli plants were mature, the florets were harvested, and the plant materials of the parents, F_1_ hybrid, and each DH line were collected and cut into small pieces 5 cm in diameter. All the samples were immediately frozen in liquid nitrogen and stored at − 80 °C. Then, the frozen samples were dried in a lyophilizer (BETA 2–8 LD plus, Christ). The dried samples were powdered using an IKA-A10 (IKA-Werke GmbH & Co. KG) mill, and the fine powder was used for SF extraction and quantitative analysis by RP-HPLC according to methods described in our previous reports^3,22,56^. According to mixed major gene plus polygene inheritance analysis, genetic analysis of sulforaphane content in the DH population and parental lines was performed following our previous report^57^. The maximum likelihood method based on the iterated expectation conditional maximization (IECM) algorithm, was used for estimating the distribution parameters. The genetic analysis (parameters) were carried out by a least-squares method in the optimal model choosen by the Akaike information criterion (AIC).

### Genotype analysis and QTL mapping

Genomic DNA was extracted from the young leaves of the two parents, the F_1_ plants and the DH families using the hexadecyl trimethyl ammonium bromide (CTAB) method, and DNA quality was detected by 1% agarose electrophoresis. A total of 176 individuals were genotyped using 438 SSR markers. These SSR markers were obtained from the *Brassica* website (http://www.brassica.bbsrc.ac.uk and http://www.brassica.bbsrc.ac.uk) (398 pairs), *B. oleracea* genome (01–20) (2170 pairs) (Table S2)^31^, and *B. oleracea* expressed sequence tags (ESTs) were obtained from the National Center for Biotechnology Information database (978 pairs)^58^. For PCR, the reaction volume of 10 μL contained 5 μL of a 2X reaction mix, 0.5 μL of the forward primer, 0.5 μL of the reverse primer, 2 μL of genomic DNA template and 2 μL of ddH_2_O. The cycling conditions were as follows: 5 min 94 °C; 40 cycles of 30 s at 94 °C, 30 s at 55 °C and 1 min at 72 °C; and a final extension of 10 min at 72 °C. Thereafter, 6% denaturing polyacrylamide gel electrophoresis (PAGE) was used to separate the PCR products.

QTL analysis was carried out via inclusive composite interval mapping (ICIM-ADD) with QTL IciMapping version 4.2 software (http://www.isbreeding.net). The critical LOD score for a significant QTL was set at 3.0, and the walking speed for the genome-wide scan was set at 1 cM, through which both the additive and dominant effects of a QTL can be estimated^34,59^. The LOD threshold for each significant QTL was calculated via 1000 permutations at *p* < 0.05. Conditional QTL analysis between 86,101 and 90,196 in the DH population was conducted using the software QGAstation2.0 based on a mixed model for the complex quantitative traits^60^.

### Statistical analysis

The calculation of descriptive statistics, frequency distributions and one-way ANOVA was performed using SPSS 19.0 software (http://www.spss.com). Additionally, Microsoft Office Excel 2010 software was used for data entry and simple analysis.

### Human and animal rights

This article does not contain any studies with human participants or animals performed by any of the authors.

## Supplementary Information

Supplementary Information.

## Data Availability

Data supporting the current study can be obtained by contacting the corresponding author (lizhansheng@caas.cn).

## Abbreviations

DH: Doubled haploid
QTLs: Quantitative trait loci
BIP: Biparental populations
ICIM: Inclusive composite interval mapping
SSR: Simple sequence repeats
ESTs: Expressed sequence tags
cM: Centimorgan
LOD: Logarithm of odds
PVE: Phenotypic variation explained
*B. oleracea*: *Brassica oleracea*
SF: Sulforaphane
GRA: Glucoraphanin
GLS: Glucosinolate
PRO: Progoitrin
NAP: Gluconapin
GER: Glucoerucin
GBS: Glucobrassicin
OHGBS: 4-Hydroxyglucobrassicin
NeoGBS: Neoglucobrassicin
GBN: Glucobrassicanapin
SIN: Sinigrin
GIV: Glucoiberverin
GIB: Glucoiberin
ALY: Glucoalyssin (ALY)
GST: Gluconasturtiin
Ali-GLS: Aliphatic glucosinolates
indolic GLS: Indolic glucosinolates
Aro-GLS: Aromatic glucosinolate
MY: Myrosinase
ESP: Epithiospecifier protein
CTAB: Hexadecyl trimethyl ammonium bromide
CV: Coefficients of variation
Add: Additive effect
