# Supplementary material for: Fine mapping of the major QTLs for biochemical variation of sulforaphane in broccoli florets using a DH population
Source: Sci Rep. 2021 Apr 26;11:9004. doi: 10.1038/s41598-021-88652-3 (PMC8076207; doi:10.1038/s41598-021-88652-3)
Supplement: Supplementary file 1 — Supplementary Information. [file 41598_2021_88652_MOESM1_ESM.docx]

**Fine mapping of the major QTLs for biochemical variation of sulforaphane in broccoli florets using a DH population**

**Zhansheng Li*****, Yumei Liu, Suxia Yuan, Fengqing Han, Zhiyuan Fang, Limei Yang, Mu Zhuang, Yangyong Zhang, Honghao Lv, Yong Wang, Jialei Ji**

*Corresponding Author: Zhansheng Li, E-mail: lizhansheng@caas.cn, ORCID: 0000-0003-0132-2741

Author list:

**Zhansheng Li**, E-mail: [lizhansheng@caas.cn](mailto:lizhansheng@caas.cn), Key Laboratory of Biology and Genetic Improvement of Horticultural Crops, Ministry of Agriculture, Institute of Vegetables and Flowers, Chinese Academy of Agricultural Sciences, Beijing, China

**Yumei Liu**, E-mail: [liuyumei@caas.cn](mailto:liuyumei@caas.cn), Key Laboratory of Biology and Genetic Improvement of Horticultural Crops, Ministry of Agriculture, Institute of Vegetables and Flowers, Chinese Academy of Agricultural Sciences, Beijing, China

**Suxia Yuan**, E-mail: [yuansuxia@caas.cn](mailto:yuansuxia@caas.cn), Key Laboratory of Biology and Genetic Improvement of Horticultural Crops, Ministry of Agriculture, Institute of Vegetables and Flowers, Chinese Academy of Agricultural Sciences, Beijing, China

**Fengqing Han**, E-mail: [hanfengqing@caas.cn](mailto:hanfengqing@caas.cn), Key Laboratory of Biology and Genetic Improvement of Horticultural Crops, Ministry of Agriculture, Institute of Vegetables and Flowers, Chinese Academy of Agricultural Sciences, Beijing, China

**Zhiyuan Fang**, E-mail: [fangzhiyuan@caas.cn](mailto:fangzhiyuan@caas.cn), Key Laboratory of Biology and Genetic Improvement of Horticultural Crops, Ministry of Agriculture, Institute of Vegetables and Flowers, Chinese Academy of Agricultural Sciences, Beijing, China

**Limei Yang**, E-mail: [yanglimei@caas.cn](mailto:yanglimei@caas.cn), Key Laboratory of Biology and Genetic Improvement of Horticultural Crops, Ministry of Agriculture, Institute of Vegetables and Flowers, Chinese Academy of Agricultural Sciences, Beijing, China

**Mu Zhuang**, E-mail: [zhuangmu@caas.cn](mailto:zhuangmu@caas.cn), Key Laboratory of Biology and Genetic Improvement of Horticultural Crops, Ministry of Agriculture, Institute of Vegetables and Flowers, Chinese Academy of Agricultural Sciences, Beijing, China

**Yangyong Zhang**, E-mail: [zhangyangyong@caas.cn](mailto:zhangyangyong@caas.cn), Key Laboratory of Biology and Genetic Improvement of Horticultural Crops, Ministry of Agriculture, Institute of Vegetables and Flowers, Chinese Academy of Agricultural Sciences, Beijing, China

**Honghao Lv**, E-mail: [lvhonghao@caas.cn](mailto:lvhonghao@caas.cn), Key Laboratory of Biology and Genetic Improvement of Horticultural Crops, Ministry of Agriculture, Institute of Vegetables and Flowers, Chinese Academy of Agricultural Sciences, Beijing, China

**Yong Wang**, E-mail: [wangyong03@caas.cn](mailto:lvhonghao@caas.cn), Key Laboratory of Biology and Genetic Improvement of Horticultural Crops, Ministry of Agriculture, Institute of Vegetables and Flowers, Chinese Academy of Agricultural Sciences, Beijing, China

**Jialei Ji**, E-mail: [jijialei@caas.cn](mailto:jijialei@caas.cn), Key Laboratory of Biology and Genetic Improvement of Horticultural Crops, Ministry of Agriculture, Institute of Vegetables and Flowers, Chinese Academy of Agricultural Sciences, Beijing, China

**Table S1. The AIC values of sulforaphane (SF) content in different genetic models**

| Model | AIC value | Model | AIC value | Model | AIC value |
| --- | --- | --- | --- | --- | --- |
| A-0 | 1974.41 | E-1-4 | 1870.77 | E-2-7 | 1925.54 |
| A-1 | 2014.48 | E-1-5 | 1870.77 | E-2-8 | 1925.54 |
| B-1-1 | 1957.92 | E-1-6 | 1946.77 | E-2-9 | 1925.54 |
| B-1-2 | 2016.96 | E-1-7 | 1950.72 | F-1 | 1865.63 |
| B-1-3 | 2014.97 | E-1-8 | 1950.72 | F-2 | 1993.99 |
| C-0 | 1923.54 | E-1-9 | 1933.79 | F-3 | 1955.41 |
| C-1 | 2030.72 | E-2-0* | 1845.79 | F-4 | 2017.73 |
| D-0 | 1925.55 | E-2-1 | 1865.33 | G-0 | 1852.70 |
| D-1 | 2032.47 | E-2-2 | 2036.32 | G-1* | 1829.27 |
| E-1-0* | 1843.79 | E-2-3 | 2034.30 | G-2 | 2028.42 |
| E-1-1 | 1875.74 | E-2-4 | 1927.59 | G-3 | 2009.24 |
| E-1-2 | 2034.32 | E-2-5 | 1854.06 | G-4 | 2034.14 |
| E-1-3 | 2032.28 | E-2-6 | 1927.54 |  |  |

Note：AIC standard for A kaike’s information criterion, * presents the candidate models (in red)

**Table S2. 2170 pairs of SSR primers designed based on *B. oleracea* genome**

| No | Primer code | Forward primer (5′- 3′) | Reverse primer (5′- 3′) |
| --- | --- | --- | --- |
| 1 | scaffold1173 | AAAACATACAAACTCGGCGTG | AGGTTTTGCGAAATGGTGAC |
| 2 | C18570725 | AAAAGAAGGGCTTCTCCGTC | CCACAGACAACAATCCAGCA |
| 3 | scaffold57260 | AAACTTGCAGGGGAGAAGGT | GGCATCTTCCTCTTCGTCTTC |
| 4 | scaffold3690 | AACCCTTTCACGGTAAGCCT | TGCCAAAAACCGAACTCTTC |
| 5 | scaffold20851 | AAGACGAGTCCACTGCGGTA | CCCATCGTGTTATGTTGCAT |
| 6 | scaffold18 | AAGATATACAGTCCCGGCCC | GAAACAACTCACCAGTGCGA |
| 7 | scaffold13054 | AAGCGGCATAAGTTTGCTTC | CAGGTCACTTTCAGGTGGGT |
| 8 | scaffold6568 | AAGGAATGTCTTCACACGCC | AACCATAGCTTAGCTTGTAAATAAAAA |
| 9 | scaffold29929 | AAGGCCATGGATGAACTGAC | GTTTGGTCCATCTCGTTCGT |
| 10 | scaffold23046 | AATCCACAACTCTTTCGTCCA | CCAAATGGTTTAGGATACAACGA |
| 11 | scaffold36289 | AATCCATTGCCTTCATTTGC | TGACCTGAAACCCACAACAA |
| 12 | scaffold26032 | ACAAAGCATCAACATCGTGC | GCGATTCATGGAAGCCTACT |
| 13 | scaffold2528 | ACCAAGCCGATGACAAAAAC | CTACGGCTGAAGAGGAAACG |
| 14 | scaffold3999 | ACCCACTGCACAGTCACAGA | TTGCGCCACTGTGAAGTCTA |
| 15 | scaffold14381 | ACCCGAAGGCCTAGTTTGAT | ACCAATGAAACCTGCTCTGG |
| 16 | scaffold1944 | ACCGCACTTATTTCACCTCG | TTTGAGTTCATAACATTTCCAATGA |
| 17 | scaffold59311 | ACCTACGAACAGGGAAACCA | TGCAAATAGATCGAAGAGAGAACA |
| 18 | scaffold12712 | ACTGGAAAAGTGAGCACGGT | TGTCAAGGGCAGACAACAAG |
| 19 | scaffold11978 | AGAAATTAGGGCATGGCTCA | TAGGTCCGAGATTGTCGTCC |
| 20 | scaffold415 | AGACACACCGGAACCTCATC | ACACCCTACCGTATGGAAAAT |
| 21 | scaffold34103 | AGAGAAGGAAGTGGACGCAA | TCAACTGGAAGCAGTGGAGA |
| 22 | scaffold30268 | AGCTTATTCCGTTTGGCTGA | AAGGCTACCCCACATTCACA |
| 23 | scaffold26600 | AGGAGCTGGACCAGAAGGTT | GAGGGACCATTGTTCTGCAT |
| 24 | scaffold3004 | AGGCCAACTTTGGAGGTCTT | GCATGAGTCAGCTTCAGATTCTT |
| 25 | scaffold37008 | ATAATTGGCAGTCGACGAGG | CCCCATCATGACGCTCTAGT |
| 26 | scaffold12388 | ATCCCAATCATCTGAACCCA | GTCGTAGCAAGTGGTCAGCA |
| 27 | scaffold26222 | ATCCGAACCTTCTCATGCAC | TCATGAATCCGTATTTTTGTCG |
| 28 | scaffold27468 | ATCTCGAATCAAGGTTTGCG | TTCATAACAAACAAGAAAACAGGAA |
| 29 | scaffold5911 | ATGACGAGGCCATAGGTGAC | CAACGACACTTGCAAAATGG |
| 30 | scaffold2116 | ATTGTCTGCCTCAGTGCCTT | CGCCGGAATACCCCTAGTAT |
| 31 | scaffold6117 | CAAAAGTGGTTGTGCATTGG | GCTCTTGGAATGGCGTCTAC |
| 32 | scaffold548 | CAAATTTGTTAAAGACGAAGAGGA | TCAAAATGGCAGAGGGGTAG |
| 33 | scaffold14784 | CAACTCGAGGCAACACTCAA | CACCATTCAACACATTCATAGCA |
| 34 | scaffold39309 | CAATCGAGCTCCAAAATCAT | TCCTTTCCACAAAAACGACA |
| 35 | scaffold47497 | CAATGGTCTGACCGTCAATG | GAGCACTGTGAAACCAAGCA |
| 36 | scaffold6506 | CAGAGGGAAACATGTGGAGA | TCAACGCATCTTTGTCAACAC |
| 37 | scaffold19684 | CAGCGTTCCACTAACGAACA | GAGGACATAAGTCTGGGGCA |
| 38 | scaffold23551 | CAGGGGATGTATCATTTGCC | GCCGCGTTATAAGGAGTCAA |
| 39 | scaffold62750 | CATACCCCCAAGCGAGAAG | TTCCAACACCGCTTCTTACC |
| 40 | scaffold15539 | CATTAAATGAAACCTTTTTGGG | TGTGTCGCACTCACAATCAA |
| 41 | scaffold36667 | CATTCGTACACTCTTGTTCTTCG | TTGCGTCTTTGTTGGATCTC |
| 42 | scaffold5692 | CCAATAGGAAATTTTTACCCAAA | CCATCCTGAACGAAGAGTCC |
| 43 | scaffold12457 | CCAATCCACTCGTCTTGAGAA | CGTTTCCATGTCACAGGTTG |
| 44 | scaffold25473 | CCACAAAGTGCTGTGTTTGC | AACCAAAATAACTCAGATCATAAAACA |
| 45 | scaffold5874 | CCAGACATCTCTGAGTCCCC | CCGTAAATCCCGTACCACTG |
| 46 | scaffold23430 | CCAGGATCTTTGGAAGGGAT | CAAAACCCCAAAGCTGAAAA |
| 47 | scaffold322 | CCAGTAGGATTGGACCTGACA | CTGGAACAACAACGCCTCTT |
| 48 | scaffold11909 | CCAGTTTGTAAGCACCATTCC | CGAAGATTCAATGCACGGTA |
| 49 | scaffold33721 | CCATGCATGTTGTTTCCTTTC | GCGTTACCATTCATCCAAAGA |
| 50 | scaffold26501 | CCCGTCAGCTCTCAAGAAAC | CCAGTTGCTTTGGGTTTTGT |
| 51 | scaffold4850 | CCCTCCCTTATCATAGGCTC | GCAAACGCAGATCACTGACA |
| 52 | scaffold24504 | CCGGGTTTTCGCTTATTCTA | TCCACTGCTCCTACTCCGAT |
| 53 | scaffold9792 | CCTCGGTGCAGTTTTGAAAT | AAAACAAAGCCAAAACCACG |
| 54 | scaffold1917 | CCTGAAAACGGAGGTTTGAA | GAAAACGTAGGGTTAGGGGC |
| 55 | scaffold61776 | CCTGAGTGTCCGGAGAATTG | GCTGAGCTTGGAGGTCAAAA |
| 56 | scaffold40892 | CCTGCTTGGAAGGACAAGAG | CCTGAGTTGCAAAAAGCTCC |
| 57 | scaffold1064 | CGACGTGGTTTCAAGACAGA | GGCAATCCATGATTTGCTTA |
| 58 | scaffold45643 | CGGATGTGCTCCAGAAAAGT | ATGGTCGGGGTCAGCTATTC |
| 59 | scaffold6003 | CGTACAGACCCTACCGGAGA | CCAAAGAAACAAGGGTTGGA |
| 60 | scaffold17373 | CGTAGGTTCCTTGTCACCCT | AGTGGATTGTGTATTTTGTTATTGTT |
| 61 | scaffold9470 | CTCGAGAAAATTCGAATGGC | GTTCCCTCTTTCTCTCCGCT |
| 62 | scaffold18122 | CTCTTCCCACCGGTATTCCT | CGAGTTCACCGAGCACAATA |
| 63 | scaffold8962 | CTGGAGCCGAGGTAATTGAG | CGCTTCAAATGTCAAGACCA |
| 64 | scaffold20896 | CTGTCATCACTGTGCCTGCT | GAACCCCAGTTAAAAGACGG |
| 65 | scaffold7918 | CTGTTGTGCAGTGTCCGATT | TGTAGTGAAAATGCAACCCG |
| 66 | scaffold15190 | CTTCTCATTTTCCCTCTAGCAA | GGAGAGAAGCTACCTATCGACTTTT |
| 67 | scaffold3004 | CTTCTCCAAATCCTGGTGGA | AAGCATCCAAACCCATCAAC |
| 68 | scaffold41213 | GAACAGACGAAACGACAGGC | AGCACCAAAAGCTATCCCAG |
| 69 | scaffold20827 | GAAGAACGATGACCCCGTTA | TAGGGATAGAAATTGGGGGC |
| 70 | scaffold15203 | GAAGGTCCTTACATGGTGAAGC | AAACACAGTGCTGATGCAGG |
| 71 | scaffold17872 | GAGCCTTTGCACACGAAAAC | GGCGTCTAATAGCATGTACAAGTG |
| 72 | scaffold15128 | GCAACGTCAAGGAAAAGAGC | CACAAGTGCCGTGTAGGAGA |
| 73 | scaffold50471 | GCACTTAACTCCCCACCGTA | GCAAAGACGCAATCTTTCCT |
| 74 | scaffold41331 | GCAGCAAATAGGTTATCAGCC | GCACGCAATTGCATAGTCAG |
| 75 | scaffold25281 | GCAGGGATTCGAAATAGCAG | AGTAGGAGGGGAGGTGTCGT |
| 76 | scaffold4534 | GCGGAGGCTTAGCTGTAATG | CCAACTGAGCCAAGAACCTT |
| 77 | scaffold30340 | GGACATAGGATCGCCTTGAG | GACGCCTCCTGCAACTCTAC |
| 78 | scaffold38682 | GGATTGCAGAGGACGAGGTA | GCGCAATTGCTGTTAAAAAGA |
| 79 | scaffold4509 | GGCGTTTTGAGAGACGAAAG | ACTTGATCCCACCTTCATGC |
| 80 | scaffold59674 | GGGAACACAAGGAAACGAGA | GCTGCTTTTCCACCTAGTGC |
| 81 | scaffold10362 | GGGCATTTCTTTTCGTGGTA | CTCACGGACTTTGCTGACAA |
| 82 | scaffold32319 | GGTGATCAACCATTGCAACA | GACTCCACCGAAGGTACCAA |
| 83 | scaffold13305 | GTCGAGACTTCCACCGGATA | TGCCCCCACACGATTTATAC |
| 84 | scaffold10303 | GTGACCGAAGGCAATTAGGA | GAACGAGCTAGACGCTGGTC |
| 85 | scaffold4542 | GTGCCTTATTTGCCACCACT | GATGAGATTTTGACCGGGAA |
| 86 | scaffold1463 | GTGCGACAAATGCAATGACT | CAAACCTCTCTTTGCGCTTC |
| 87 | scaffold6134 | TAAAGAGACCCTTTGGCCCT | CGAAACCGTTTGAAGAGGAG |
| 88 | scaffold3145 | TAGCATGTTCGAACCGTCAG | AAATAACTGGACGCCCAAAA |
| 89 | scaffold42898 | TAGCTCCCATGAGAAGGGAA | AGATCGTACAAGACAAATGATCG |
| 90 | scaffold27548 | TCAAATCTTTAGCCTTTTGAACTTT | TAAATCAATGCAGACGCTGG |
| 91 | scaffold25277 | TCAACAAACCAGTAACCACGTT | CAACACGAGCCAGAGATGAA |
| 92 | scaffold38176 | TCAACTCCAGAACGCATGAC | ACAAGCTAAGTATGATTTTTCGTTTA |
| 93 | scaffold17331 | TCATATGCGTTTCCACAAAA | GCCGTCAGCTTGTTCTTCTT |
| 94 | scaffold5306 | TCATCTCCACACCTACGCAA | TGCCATATTAGCCATTTCTTTG |
| 95 | scaffold15734 | TCCATATCCAACAACATTCTGC | AAAAGAAACCCGCACAAAAA |
| 96 | scaffold25867 | TCCGTTCCGAAGACAAAATC | GGGTATACGCAAAACGATGG |
| 97 | scaffold33738 | TCGAAGAGCAGATTCCTAACG | CGAGTTCCCAAACCTAGCTC |
| 98 | scaffold609 | TCGGTGGTGACTGTTTCATC | CCATGCTTCTTTTTGTTGGG |
| 99 | scaffold48742 | TCTAACTTTGGATGGCAGCA | AATTATATCCCGCCCGTAGG |
| 100 | scaffold61570 | TCTCGAAGAAAACGGCCTAA | TTTTTCAGACTAGTGGGCTTCA |
| 101 | scaffold2099 | TCTCTACTTTCTCGGGTGGG | GCGTGTTCTCCTCCCTCTAC |
| 102 | scaffold20494 | TGAACCGTGACAGAGAGTGC | CCTCACCCAAAAGCCAAGTA |
| 103 | scaffold41092 | TGACGCTATGTGCCAACACT | AGAGACTTTACGTTCGGGCA |
| 104 | scaffold17456 | TGAGGAGGATCCCAAGAATG | TGGTTCTGCAGACAACGAAC |
| 105 | scaffold22364 | TGAGGTGTGTTGAGTTGCGT | TTTGGTCAGTGCAAAAGTTCA |
| 106 | scaffold7673 | TGATACGTGCATAACACGGG | GAGTGGTAAGGCACGAGAGC |
| 107 | scaffold36694 | TGATGCTGGTACTCCCACTG | TCCGGATGAATGAAAACACA |
| 108 | scaffold31641 | TGCAAATCAAACACAAAGCC | ACAACGTTGGGAAGTTCAGG |
| 109 | scaffold44837 | TGCACGTGCCAATATAAATCA | ATGAGAGATTGCACACCCAT |
| 110 | scaffold51005 | TGCGTGAGCTTTCATGATTC | CATGCCAAACAGCAGGTAGA |
| 111 | scaffold602 | TGCTCAAAGAGCCCAACTTT | CGAAGGATCAACTCTCCTCG |
| 112 | scaffold13578 | TGCTGGGATGACAATGATGT | TCTACAGCCTCGCACAAATG |
| 113 | scaffold3459 | TGCTTTCTCGTGGATGAACA | TGGAAAATTAAACGAAGCCAA |
| 114 | scaffold8857 | TGGAACGAAGACACCATTCA | TGCTCATGAAAGAAGTGCAGA |
| 115 | scaffold30303 | TGGAATCAATACAGAAAGTAATTCACA | CCTTTCCCGGAACTTTTATTTT |
| 116 | scaffold4340 | TGGAATTGTCTCCCCAAATC | CGAACTGAACCCGAATGAGT |
| 117 | scaffold8984 | TGGCGTGATGTAAAGCTGAG | GCTCACCTCTCCTTTGTTGC |
| 118 | scaffold47708 | TGGGATGCCAACACATTTTA | AACTTGGGACTATCCCGGAC |
| 119 | scaffold26011 | TGTCACGGCATCTACAGACC | GGAAGAGATTGTTGGGTTGAG |
| 120 | scaffold28148 | TTAACCAGACGAAGCCGAAC | GCCACATCGTGTCTTAGCAA |
| 121 | scaffold22309 | TTACTCACCGTTTTCTCGCC | CACGTTCGCATTGCTAAAAA |
| 122 | scaffold25947 | TTACTCCAGTGGACCCCTCA | ATGGCTATTCCAGGCCTCTT |
| 123 | scaffold7492 | TTCAGAGATTTGCGTGGATG | AAGACCCTCGAACTTACGCA |
| 124 | scaffold11085 | TTCATTGTCCAACGGTAGGG | TTCGTGACGATATGAACCCA |
| 125 | scaffold30443 | TTGAAACGGAACACAATGGA | CAGGCTAGGGAATTCCAACA |
| 126 | scaffold19642 | TTGAGCTGTGGGTACAAAGTG | TCCAATGACAATCAATCCACTC |
| 127 | scaffold41717 | TTGGCAAAGAACCCATTCTC | CGAAGCTCTTCCCCTTCTTT |
| 128 | scaffold120 | TTGTTGGTTGATGTTCCGAG | AGCACACGACGAACTCCTCT |
| 129 | scaffold29913 | TTTTCCACATTATTTTTCTATTGGTT | AAGCAGTTCCAGCTTCCAAA |
| 130 | scaffold49641 | TGATGGTGAGGGTGAGAGTG | CGCTTTTACTTACACGCACG |
| 131 | scaffold24097 | AAACCCACACATCATCTTCCA | AGCATCTTTGCTCTTGGCAT |
| 132 | scaffold28326 | AAAGCCCACATTGTTTTTGC | CGATTTTGACCCCAACAACT |
| 133 | scaffold475 | AAATGTAGTGGCTGCAAGGC | CACGCTTACAAGAGCATCCA |
| 134 | scaffold29198 | AAATTTCAACTGGACGCTTTTT | GGAGACAAGTTGGCTTCTGG |
| 135 | scaffold38482 | ACAAAGACACACCGTCATGC | CAAGAAGCATCATGAACTTGAA |
| 136 | scaffold12255 | ACCCATTGCTGATACCCAAA | TTTCCTTTTTGTCCTCACCG |
| 137 | scaffold670 | ACCGATAATCATGCCATCGT | GCTCATTCACTGATGGGGTT |
| 138 | scaffold14356 | ACCTTAAACCCGGGTCATTT | TGGATCCGGATAGTTTGATTG |
| 139 | scaffold14415 | ACGAATACAACCTTGGCCTG | ACACCGAGAAAATCAGCCAC |
| 140 | scaffold3880 | ACGGTCCCTCCCATCTTAAC | TCTCGCTCCTGCTTTCATTT |
| 141 | scaffold32130 | AGACCCCCAGAAGTATCGCT | TGAGGGAGCCTTCTGAAAAA |
| 142 | scaffold49598 | AGAGGATTTGCGTGAGTTGA | TGGTCATGTCCATGTCAAGAA |
| 143 | scaffold27207 | AGCCCAGTGTCCATCAAGAC | CTTAGACGGAAGGGACGTTG |
| 144 | scaffold8206 | AGCGGTGGAAAGCTACTTCA | CAACACCGTCGATCCCTATC |
| 145 | scaffold8655 | AGGAGACCGAGGTTTGGTTT | GCTGCCACCAAAATTAAGGA |
| 146 | scaffold10891 | AGGCGAGCACTTTCACTGAT | TCTGGGGGAGGTTCTACTGA |
| 147 | scaffold3263 | ATACACCCACGCACACACAC | CGGCCCAGATAAAGACAAAA |
| 148 | scaffold12606 | ATCGATTCGCTTGCAACTCT | GATGCTAGAGCGACCTGCTT |
| 149 | scaffold15976 | ATGAAAACCACAATCACGCA | TTTCGATGGCACAAAAATTG |
| 150 | scaffold28011 | ATGGCGTACCGGTCTAGTTG | CCTCGGGACATCGTTTACTT |
| 151 | scaffold8045 | CAAAAGCTTGATGCCACAAA | TGATTGCAAAAGTGGGACAA |
| 152 | scaffold11728 | CAAATGCGATGACAAAATCG | TGTGTGGTCCCTTAGCTCCT |
| 153 | scaffold393 | CAACAATGGAGGAGCTGGAT | ACATCAACCAACGATGACGA |
| 154 | scaffold37351 | CAACTGATGTTGCTGCTCGT | GCCAAAGAATGTGAATGCCT |
| 155 | scaffold60279 | CACCAAACCGAGATCATGTG | TTGATGCTGTTATTGGAAGCC |
| 156 | scaffold6112 | CACTGGAATCTGCAATGGTG | GATCGGTGGTGACAGATTCA |
| 157 | scaffold63505 | CAGTGTGTGTCCCAATACCG | TGCTCCTGATTTCCAGTTCA |
| 158 | scaffold10267 | CATGCACCGGTGGAGATAAT | TTGATCCGTTTGAAGGAACC |
| 159 | scaffold13076 | CATGGCATATGACCAATGGA | AAGAAAATAATATACGCCCACAAA |
| 160 | scaffold22606 | CCAAAAGCTCACTCCAAAGC | GCCGTCGAGTATTTCATGGT |
| 161 | scaffold21720 | CCAAGCACCGTTTCTTCTTC | CTCCCTTCAATCCAAACGAA |
| 162 | scaffold44512 | CCCATTTCGAAGGAGATCAA | CCTGCAAAGCATTGGATTTT |
| 163 | scaffold24311 | CCGTGGTAAAACACATCGAA | TGTGCTTACCACAAACCGAA |
| 164 | scaffold387 | CCTGCTTTCCCATCAACTGT | AACACAGCACTCGAAACGTG |
| 165 | scaffold16421 | CCTTCCAAAGTGAATGGATCA | TTTGCTCAGCCTTTCTTTGA |
| 166 | scaffold34474 | CCTTTTACAAAGGCAATTGAGA | AAAACCCTGAACACACCGAG |
| 167 | scaffold16935 | CGAACTATTTCCACGGATTGA | CCAGCTAAAACTTTATGTGGGTTT |
| 168 | scaffold15284 | CGCTCCACCTATTCCCATTA | TACCCGCCGTAACTTTTGTC |
| 169 | scaffold42764 | CGGAATCGCTTGCTCTTAAA | GGCCTAGATGAAAGTGTGGC |
| 170 | scaffold51658 | CGGATCCATATCGCTGATTC | TGTTTTCAGAGCGTGTCCAG |
| 171 | scaffold4078 | CGGCAAAAACAAGACATCGT | CCACAAATTAAGCCTTATGCG |
| 172 | scaffold59607 | CGTGGACATAACGGACAATG | CTTGAAGCTCTCCAGCAAGAA |
| 173 | scaffold1623 | CTCCAATTCCCCATCAGCTA | CATCAAGTGCATCTCCAACC |
| 174 | scaffold11334 | CTCTTCTGGTCCACCAGCTC | AACTTTTAACCCACAGAATTCCA |
| 175 | scaffold12750 | CTGGCTGGGACCCTTTTAGT | TGGTCGGAAAGTTACGGTTC |
| 176 | scaffold56471 | CTGTCCGTGGGTACGTTTCT | GTTTGAAAGGCGAAAAACCA |
| 177 | scaffold6032 | GAATGGATCGAGTCGCTTCT | GGCAGAAATTAATCAAGGTGC |
| 178 | scaffold6244 | GACCAGGTGAGTCCTGCTTC | TCTGAAGCTTCTGGCAATCA |
| 179 | scaffold4225 | GATGCCTTCTCAAGGAGCAC | GCCTGAAGAGTTTGGGAACA |
| 180 | scaffold32373 | GCATCTGATCTTTCCGCTTC | GGTTTTGTTTTGATTGGGACA |
| 181 | scaffold11888 | GCATGAGATTCGTGCAACAT | GTAGGGCGGGTTTACCCTA |
| 182 | scaffold12492 | GCCAAGTTGACCAGAGCCTA | GTGAGCTTGCAATCATGGAG |
| 183 | scaffold1942 | GCCTGGGGATGTTTGATATG | GGCTTGGCTAATGAGCAGAC |
| 184 | scaffold6905 | GCGGAATAAGTTTGTTAGTGCTC | AGTTTTGGCCACCAAGTAAA |
| 185 | scaffold17198 | GCGTTTTCTTGTAGGGCAAG | TGTTTAACATCTCCTCCCGC |
| 186 | scaffold20268 | GCTCCCTACACCAAAACCAA | GCTCATGGGAGTGGATAAGC |
| 187 | scaffold4094 | GGATGTGCAACGTGAGTTGT | CCCCGTTTTCATGTCTATTG |
| 188 | scaffold4662 | GGCGTCGCTAACTTTGGTAG | GTGGAAAGCAACCCACTGAT |
| 189 | scaffold45034 | GGTACTCGAACCGTTCCAAA | GACGAATTTACCCGTGTGTG |
| 190 | scaffold27681 | GTGGCTCACGACAGAACAGA | TTCCAAATCCACCTCACCAT |
| 191 | scaffold14143 | TCCATGTGTTCATAAATTGAAGTG | TCATGTCATCCTCCGAATCA |
| 192 | scaffold3141 | TCCTCTGATGAAACGCAGTG | CACCCCATCATCTTCCTTGT |
| 193 | scaffold63075 | TCTACCAAGATGGCAAGGGT | TGGTGGCATACGACCTACAA |
| 194 | scaffold13496 | TCTGCCAAATTGACCAGAGA | TGTAAATGGAACAAGCAAGCA |
| 195 | scaffold44578 | TCTGCCATTGTTCTCACAGC | CGACCCTTCATGTTCGATTT |
| 196 | scaffold9869 | TGAACAAGCCATCTGTGAGC | TGCTTGTTGAGTTTCCCACT |
| 197 | scaffold13830 | TGAGTCTCCACATCCACGAG | GCCAGGCCTATTCATGTGTT |
| 198 | scaffold785 | TGCATTTGTTTATTTAGTTGTAAGAGG | ATACTTCCGCGTTTGTGACC |
| 199 | scaffold40523 | TGCCATTTATTTGCGTCTGA | CCAACGTTCCTAATCTAGCCC |
| 200 | scaffold62792 | TGCTTAAAGGTCTTCCCCCT | TTTTCTGGATTCTGGAACGC |
| 201 | scaffold56677 | TGCTTTATTGCTCGTCGTTG | CTCCTCGTCTGGAGTTTGCT |
| 202 | scaffold20102 | TGGGTCCACGTGTTGTAAGA | CAGAGGTGATGACTCGTTGC |
| 203 | scaffold45589 | TGGTTTCGGCATAGGAGAAG | CCTGGTTCACAAACGGTCTT |
| 204 | scaffold9413 | TTCCGACGGGGTTTTCTAGT | GCATAACATCTTTGTGCCCC |
| 205 | scaffold25790 | TTCCTTGTGCAGATTGTTGC | CACGGTCCACTACAAAAGCA |
| 206 | scaffold11649 | TTGAGGCCCATCAGATTTTC | AAAAGGAGGAGAGATGAGGGA |
| 207 | scaffold30871 | TTGATTTGGATCGTTGATTGA | TTCTTTGGCATTGCTTGTGA |
| 208 | scaffold16082 | TTTGTGTGTTTTTATTTGACGGA | TGCCTCAAACACGTTTAACTCT |
| 209 | scaffold2241 | CGTACACATCACCCTGAGACC | TGCCCCCAATCTTCCTAAA |
| 210 | scaffold49473 | AAAATCGGGGGTTATCTTGC | TTGGCCAAAGTTTCTAGCGT |
| 211 | scaffold9468 | AACATACGACCTCGAGACGC | ACACCACCACCAAGTGTCAA |
| 212 | scaffold16253 | AACTCGTCCTTTCACGGTTG | GTAACCTGCACCACCATCCT |
| 213 | scaffold45862 | ACAAAAGACAAATGGTGCGA | TTTTCTTTGGATTTCACATGCT |
| 214 | scaffold5462 | ACTTCCAATGGGGGTTCTTC | GGCGTTCTTTGCTATACCGT |
| 215 | scaffold63505 | AGGACATGCAGTCATCATCAA | TTAGGTAGGGCCAGCTCAAA |
| 216 | scaffold2177 | AGGCGAATCTTGAAAGGACA | CCTGTGAATGGAAAAACCCA |
| 217 | scaffold47852 | AGGTAGACGTCACTCGCCAT | ATTCAATCAAGGGGTTTCCC |
| 218 | scaffold28042 | AGTCCGGTAAGTCGGTTCCT | GAGAATAACCATATAAAGTCACAACAA |
| 219 | scaffold16356 | ATGGACTGTGTTTTTGGTCG | ATGAAACCAAGGCAAAATGC |
| 220 | scaffold32275 | ATTCCAACAAAAACGATGCC | TAAGGAGATCGAAGGCTGGA |
| 221 | scaffold41881 | CACGACTCCACTAACTCGCA | AGTCAATGGTCGACGGAATC |
| 222 | scaffold24249 | CAGAACCTCCTTGCGTATCC | ATGCTCGTCAGGCAACTCTT |
| 223 | scaffold13199 | CATGGGATGCAAGGAGAGAT | TTGAGCCAAGCGTGTGTATC |
| 224 | scaffold6178 | CCCAAGTTCGCTTAATGGTT | GTTCTGGGGCAAATGACTGT |
| 225 | scaffold52966 | CGGTACATGAAACATTGGCA | CCCAATTCAAGTACGACGGT |
| 226 | scaffold3201 | GACCGTCGCAGGTACATACA | ATCAACTCCTACCACCGTGC |
| 227 | scaffold7068 | GCCCCATCCATATACCATTG | ATATGGAGGCGTGGCATTAC |
| 228 | scaffold18633 | GCTCAAGAGGAGTCGTCGTC | GCCAACTATCAGCCTTCTCG |
| 229 | scaffold14232 | GCTCAGACGTCTCTGAACCC | ACTGTGAACTGGAATTGGGG |
| 230 | scaffold5214 | GGACTGGAGTTCACTTGGGA | CTTGTTCTTGCGTAGCCACA |
| 231 | scaffold20419 | GTGGGCTTCACCTTTGGTTA | TCAAAACCATGCTGATCGAA |
| 232 | scaffold28389 | GTGTTGACATGGCTTTGGAA | TCACACGTAGACGAAGTGAACA |
| 233 | scaffold9289 | TAATCTGACGTGGCGCAATA | GATCCATTTGCGGTCCTAGA |
| 234 | scaffold3770 | TAGAGGACGTGGACCCTCTG | CATCCAAACTTACTTACCACCCA |
| 235 | scaffold34329 | TCAAATTCTGTTGTTCAAAATATCG | CGATCCCCGTAGTAAAAGATT |
| 236 | scaffold1880 | TCAGACATCTCTAAGCCCCC | GTGGCAAAGGTGAGGGTCTA |
| 237 | scaffold29555 | TCGTTGGTCTTGCCTTTCTT | CGTATTTCTTGAGTGCAAACG |
| 238 | scaffold33448 | TCTCCTTGCATCGGCTAAGT | TTTTGGGCTGTTTAGTTCCG |
| 239 | scaffold10238 | TGAACGTAACCGCGAGTTTT | GACCCCGATGAGAAAACAGA |
| 240 | scaffold29 | TGCTGCTGCTACCATTGAAC | TGCAGGTAAACGGTGAAAGA |
| 241 | scaffold5382 | TGTGCCCTGCAATCTCATAA | TTTCGAAACATGATCCGTCT |
| 242 | scaffold33901 | TTAGCATGCTGAGCGAACAT | ATCTCTTGGTCCTCCGTCCT |
| 243 | scaffold24088 | TTCAAACTTTGGAAGGCTCC | GCAATGCGTGGAACTATCCT |
| 244 | scaffold54153 | TTCGTGAAACAGTTGAGAACTTG | TTTCAACGAAGGAAGGCTGT |
| 245 | scaffold15194 | TTGGTTGGTATTCTACGACAAA | TAGTTCGATGGGCTTTTTGG |
| 246 | scaffold15339 | TTGGTTTGGCTTCTTCTTCC | AGCACGGTTCAGTTTTCCAA |
| 247 | scaffold24998 | TTTCAAATTAGACCGGTTCG | TTTTAATGGATTTACGTATCACACAA |
| 248 | scaffold12318 | TTTGATTCGGGTAGGACCAG | TTTGTCAACGTCTATGCGCT |
| 249 | scaffold15776 | TTTGGAATATTATCATTTGAGTTGGA | TGGCCAGATAACGTTTTTCC |
| 250 | scaffold17712 | ACAACCCAAACCCAACCATA | GCGTTGTTGGTCCACAATTT |
| 251 | scaffold25617 | ACGTAACAATTTTCTGCCGC | CATGCAAACACATCAAAGGG |
| 252 | scaffold1845 | ATGCATGAGGATGTTGCTGA | CTTCCCGGGTCATCTCCTAT |
| 253 | scaffold10380 | ATGCGGACATTAGCATGACA | TTGGGGATATGTCACCGATT |
| 254 | scaffold23040 | CAACCCCAAATCAAACAACC | TGGTTTCTGCGTAATGGTGA |
| 255 | scaffold47211 | CCATTTCTGATACCTAGCCCA | TCAGCATGCGACTAGACGTT |
| 256 | scaffold2318 | CCCAATAACTGCGTCACTCC | AAGAGCTAGGCTTCGCCTGT |
| 257 | scaffold16811 | CCTCCAACCTCATCTACCCA | TCGAGGAAAGATGTTGTAAGCA |
| 258 | scaffold5326 | CCTCTCCATCTGCCATTTTC | TTCTCCTCGCAAACTATCAGG |
| 259 | scaffold18545 | CGACATGAGCTGGTTCTTGA | ATTCGGTTTGTCTGCAAAGG |
| 260 | scaffold2520 | CTTGGAAAAAGGTTGGGACA | CGGCATCTAAATCTGGTTCC |
| 261 | scaffold2146 | GCATCAACTGTTCCACCCTT | AGACGAAGGGGATCACAAAC |
| 262 | scaffold53264 | GCCTTATCAGCATGTGGCTT | AGAGGCTTACCATGCTTCCA |
| 263 | scaffold37599 | GCGTAGGTCGTTGACAGACA | GCTCCCATGGTAGTGTTGGT |
| 264 | scaffold25439 | GCTGAAGGGTGTCAAGTGGT | TTGCTGCTGCTTCTATTGTGA |
| 265 | scaffold18680 | GGTCTAGGCGAAGTTTAGGC | CGGTAACTCGGTCCTAACCA |
| 266 | scaffold5109 | GTCGAGACATAAGCATGCGA | GCCAAGAATGATTTGCCAGT |
| 267 | scaffold15967 | GTTCTTAGGCTTGTGCCTCG | TGTTTATGATTTTGACCAGGGA |
| 268 | scaffold7178 | TCAAAACCCTTCTCAATTCTCA | GCCAAGAAAGAAAAATCCGA |
| 269 | scaffold29704 | TGACTCCTCGGTCTTCGTCT | TTGAGCTTGGTTTAGATCGTGA |
| 270 | scaffold7687 | TGAGGGGATTGCTAGTGTCC | TTCTGTGAGTGCTACGGTCCT |
| 271 | scaffold13333 | TGATGTGAGATCTTGATGGGTC | TCAACCCCATGTAACCACAA |
| 272 | scaffold13036 | TGTCATCAAACATCTCTGCCA | ACACTCGATGCTGACATTGC |
| 273 | scaffold7045 | TTAAAAGCGATCGTGGAAAAA | AATCTAATGGCGATTGGCAG |
| 274 | scaffold4163 | TTTGAAACGGAGGACATCAA | CCATGTGATCGCCATTGTTA |
| 275 | scaffold25007 | TTTGGTAGGTTTGCTCAGGG | AGCATCCAAAGAGAAAGCCA |
| 276 | scaffold28095 | TTTTGTTTTGAAAATTGCGG | CCTTGTTGGTTTAGGTTTTGGA |
| 277 | scaffold20113 | ACGTTACACAAATGGCCACA | GTCCAAGACCATCCATTGCT |
| 278 | scaffold61833 | AGAAGAATCCTCCCCTTCCA | TATTATGGCTTCGTGTGGCA |
| 279 | scaffold2924 | CAACCATGGATGATGATGGA | CCAGGAAGGAGAAAACCCTT |
| 280 | scaffold13882 | CACACTTTTCCGCGTTATCA | TATGTCATTGACCAGTGCGG |
| 281 | scaffold33721 | CGGAACCTGATTTTGATGCT | ACCAGACGCAGAAGAGAGGA |
| 282 | scaffold28775 | CTGCACAAATAACTGGTCCATC | TTCATCCCTCCCACACTAGG |
| 283 | scaffold7541 | GAGGTTTCAGCTTCAGGCAC | CAACAGAGCCGAGAATCACA |
| 284 | scaffold33708 | GAGTTCGGACACATCAGGGT | GTCATTGAAATGGCATGCAG |
| 285 | scaffold8365 | GCCCTTCTCCTTTAAGCCTC | GATACGGCGAAAAAGAGCTG |
| 286 | scaffold36368 | TCAACGCTTCAACCATTCTTT | CGTCGTCTAAATCGATGTGTAA |
| 287 | scaffold38233 | TGCGTCACAACCCTTTCATA | TGCGCGGATCTTAAGCTAGT |
| 288 | scaffold12883 | TTGTGGAGAGTCCATTGCAG | TGTGTTGATTCTTGATGGGC |
| 289 | scaffold5204 | AAAAAGCGAGGGTGGAAAAT | GAGAAACCCACCACGAAAGA |
| 290 | scaffold31050 | AAAACCCTTCCATCAGGACC | GAAGAGACGTGGGGTCGTAG |
| 291 | scaffold47064 | AAACCGTCAACATCGAGGAC | TCCGGGTGGAATCAATATGT |
| 292 | scaffold21101 | AAACGCTTCTTTGTTCCAGC | CCGGCGGTAAAATTGACTAA |
| 293 | scaffold3763 | AAAGGGAAACACAGATTGCG | CGGCTCTCTGACCAGTTTTC |
| 294 | scaffold52269 | AACCCATTGCAATTTTCAGC | TCCAGGTCATTGCTAAGGAAA |
| 295 | scaffold15765 | AACCGTAAATCGCCTTTGTG | TTTAAGCAGTATTGGCCGTC |
| 296 | scaffold13871 | AACGTCGACTCTGTGTGTGG | GTGGGGGAGAATTGTAACGA |
| 297 | scaffold28484 | AACTCCCTGCAAAGGTGAGA | TTGGCGGCAACTAAAAGAAG |
| 298 | scaffold22767 | AACTTTCCGCAAATGTTGGA | TGGCCAAACTCGTAAGAATG |
| 299 | C18419817 | AAGAATCAGCGGAAGAGCAA | CAAGGCCTAATCATCCGAAA |
| 300 | scaffold1422 | AAGAGGCGCATCAAAGGATA | GCTTTGGCCAGTTTGAAATC |
| 301 | scaffold1518 | AAGGTTCCACCGAGAATTGA | AGGAGGAGACGAGGGAGAAG |
| 302 | scaffold35208 | AATCTGGAGCCGTGAAGATG | CGATCGTAAGTCCTACCGGA |
| 303 | scaffold60051 | ACAACAAGCCTTATCGGTGG | ACTAGCATGCCTGTCCCACT |
| 304 | scaffold55516 | ACCACCGATGATCTTTACGC | TGGATAAACATTTGGATTTTGA |
| 305 | scaffold3377 | ACCATGGCCTCTTGTATTGC | CGGAGTGTTCCCAATCAACT |
| 306 | scaffold3511 | ACGAGCCTCGATGAGGTAGA | CCTCTCAACGAAGACCAATCA |
| 307 | scaffold20922 | ACTCCAACACTCGCGGTACT | TCATCTTCCATTCCTCACCC |
| 308 | scaffold10194 | AGATCAACGAGGAGGGTGTG | GGGAAACGAAACGACAAAAA |
| 309 | scaffold18495 | AGCAACAAGAGGGGGACTTT | GCTGTTTGTTTGAACGGGAT |
| 310 | scaffold28572 | AGCACATTTAAACCGAACCG | TGCTTGGACTTGTCAGCATC |
| 311 | scaffold25977 | AGCATCATCGACCCTGAAAC | GCAACCCTAAGTCCCCATTT |
| 312 | scaffold21539 | AGCTGAACCAAGTAGCTGCC | GAGAGAGGTGTGGTGGTGGT |
| 313 | scaffold6470 | AGCTTTGACCCCATTACACG | CACGCTCCTCGTTGATGTAA |
| 314 | scaffold4835 | AGGCTCTCTCAGACACGGAG | CGGTATAAATCAAAAGGGTTGC |
| 315 | scaffold9800 | ATATTTGCCCAACCATCGAA | TGGAATCGAGATCAGGAAATG |
| 316 | scaffold23186 | ATCCTTGTCTTGTGGTTCCG | CAATGCTGAATCCAAACCCT |
| 317 | scaffold32263 | ATCGAAAGGGGGATCATAGG | AATTGGAAGCATCAACGACC |
| 318 | scaffold40254 | ATGATGGGCATATAGCTGGC | TTCATCTTCATCCTCTTACGCA |
| 319 | scaffold19605 | ATTATCCCTTTCCCCGTCAC | CTGATCACCAATGAGAGCCA |
| 320 | scaffold57459 | CAAAACGTGGCTAACAACCA | TTTCGTCGGTTTAGGTTTCG |
| 321 | scaffold1712 | CAAACACATATTTTCACAAATTTCAT | GTGAGAATTTGGCACTACGCT |
| 322 | scaffold20912 | CAACCAAACCACACATCCAA | ACCATGAGAACTGTTTCGGG |
| 323 | scaffold19860 | CAACGTTGTTGATGATCTTTGAA | TCGACACTGGCAAATGGATA |
| 324 | scaffold788 | CAAGGACCGGCACGTATATT | GCGAGTGATCTACCGAGAGG |
| 325 | scaffold4316 | CAATTGAACCCCCAAATGTC | TTCTTGGTTTTAGCCGCTTG |
| 326 | scaffold16908 | CACAAAGCAAAGCAACCAAA | CAGAAAGCCAAGAAGAACCG |
| 327 | scaffold7388 | CACAGTCCGTCGGAAGTTTT | TAAGGCTTAGAATCGCCGAA |
| 328 | scaffold3213 | CACCTCCCGCATACGTTACT | ATAATGTGGTGGATCCCGTC |
| 329 | scaffold26138 | CAGATCGAAGGTGCTTGACA | CTTAACCTGCGGGATTTTGA |
| 330 | scaffold20702 | CAGCCACTTCGTTGTTTGAA | TGGAGGAGGAGCAGAGAGAG |
| 331 | scaffold13985 | CAGGTCAAGAAGCCCAAAAC | TGAGGATTGCCTTTCCACTT |
| 332 | scaffold8127 | CATCTGTTGCCATTTGAGGA | ACAGCGACCAATGCTTCTCT |
| 333 | scaffold21742 | CATGCGGAAGCATTTGAGTA | CCGATCGAGCTCATGATAAA |
| 334 | scaffold38558 | CCAAACAAGCAAAGACCGTT | GCTTTTGGAGAGGATGGTGA |
| 335 | scaffold9785 | CCAAACTCACTTCGGTCTGA | TCAGATCAGGCCGTCTTCTT |
| 336 | scaffold52920 | CCATTAATCTTCGGGCTTCA | CAGAGACGACCATCTCACGA |
| 337 | scaffold15131 | CCCAATCAAGAGAGAAAAATTAAAA | GCTCAAGTCCCCTTCTCTCTC |
| 338 | scaffold10446 | CCCCAAAATAAGATCAACCC | TCAACTCTTGCACCGATTGT |
| 339 | scaffold10463 | CCCTATTTGCGTGCGTCTAT | GAAACCACCCATCAAGGAAA |
| 340 | scaffold16020 | CCGGAATAACCCCTATAACCA | TATATGATTTGTTGCGGCGA |
| 341 | scaffold42483 | CCGGGACAACAACCTATCAA | GGAAGTGGATCAGCTCTGGA |
| 342 | scaffold7679 | CCGGTTTTAGCAAACCGATA | TGATCTTCACTCGTGTTCCG |
| 343 | scaffold157 | CCGTATCTAATATTCCCGCC | GAAGGAAGGAGAAGAGCCGT |
| 344 | scaffold22709 | CCGTTGCGTTGATGACTAAA | AGGCGATCAATGGAAAGATG |
| 345 | scaffold40170 | CCTTCTCAAAAACATCCCCA | TTAAAGGACGCGTGTTTGTG |
| 346 | scaffold47821 | CGATCTCTGGCGTTCTTAGG | GCTGCAAAATCCCTAATTGC |
| 347 | scaffold3215 | CGATGGGAAAAATAATCATTGG | TCTGCTGGGAGTTTTGGACT |
| 348 | scaffold17986 | CGCACATTCTCAAAGTCCCT | ACTGGTCCCCACGTAGACAG |
| 349 | scaffold1339 | CGCCATTGATAGGGACATTT | GAAGGCGAAATTGAAATGGA |
| 350 | scaffold17181 | CGGATTCATCAACCTCGTCT | CTCACCGCTTCAAAATCCAT |
| 351 | scaffold24098 | CGGATTTGCAGTGGAGAGAT | TTGCCTGTTTTGCATCTTCTT |
| 352 | scaffold13358 | CGGTTCCATTTGAAACCTGT | TCCTCTGATCCACTCCATCC |
| 353 | scaffold20276 | CGGTTTGGAAACTTGGTTTG | AAGCTTCTTCTCCGTCCTCC |
| 354 | scaffold61042 | CGTCGAACAAGATGGCACTA | GCCGTAAGGAACACGTTAGC |
| 355 | scaffold32787 | CGTCGCTCTCCGAGGTATTA | ATGAATCAAATTTCCGCTGG |
| 356 | scaffold8597 | CGTTCCCATTCTCGTCCTTA | GGAGATTTCGAATGATCGGA |
| 357 | scaffold2862 | CTCATTCTTCTCCGCCGTTA | CAGAACTTTTCGACGCAACA |
| 358 | scaffold1858 | CTCGTGGTTCAGGACTCCAT | GTGGCGAGTTTTGATTTGGT |
| 359 | scaffold15317 | CTCTCAGGTCCACCACCATT | GCAAGAGCAGGTAAGTTTTGG |
| 360 | scaffold24814 | CTCTCCGATCGTGTCCGTAT | GTCCAACACCGTCTTCCATT |
| 361 | scaffold11606 | CTGATGAGGACCAACGGTTT | GCAAGAGCCAACAAACAACA |
| 362 | scaffold17331 | CTGTAAGAGGCTGGACTCGG | TTCAAGCGCCACTTTTTCTT |
| 363 | scaffold2160 | CTTCCAAAACGTCGAGCTTC | CGCGTAGAAACCCGTTAAAA |
| 364 | scaffold7370 | CTTCCTCACACCCTTGAAGC | AGCATTGCGTGAGAGGATTT |
| 365 | scaffold14151 | CTTGGGGAAAACTCCTGTGA | CTTGGCTTCCAGCTTCTGAC |
| 366 | scaffold12209 | CTTGGTTCCTCTGGTGGGTA | CAGATACTCCGGCCTCACAT |
| 367 | scaffold4533 | GAAACTGGTGCGGAATGAAT | TCTTTGGTGATGCCACGATA |
| 368 | scaffold31756 | GAAAGACCACCAGAAAACGC | AAAGCATTTGCAGCTCCAGT |
| 369 | scaffold18893 | GACGTTGCTGTTGAACGAGA | GCTCCGTCTATGACTCCTGC |
| 370 | scaffold56094 | GAGGCGATTCGTCGTTCTAA | ATTGGCCATTTCAGTGAGGT |
| 371 | scaffold639 | GAGTCCCACCGATCAAGAAA | GATGAGAGAGATCAGGGAAGGA |
| 372 | scaffold60249 | GCAACACTTGCTGTTTTGGA | GCTGAGGTAGGAAGGGAAGG |
| 373 | scaffold343 | GCAAGTCTGGAAGGCAAAAG | GCATGAGACACTCCTACCCC |
| 374 | scaffold17114 | GCAGACACACCTCGATCTCA | CTCAAAAACCCCTAAACGCA |
| 375 | scaffold29640 | GCCGTTTTCATGTTTCACCT | CTGCCATAACCCTAGCGAAG |
| 376 | scaffold48488 | GCGGGATGAGAAAAGTTCAA | CTCTTCTTCCTCCACATCGC |
| 377 | scaffold11066 | GCTTCTCCACCTATCATGGC | CATCTCTAATGGCACGAGCA |
| 378 | scaffold6039 | GCTTGAATATCGCAAGCCTC | GCCGACGACTAGTTCCTGAG |
| 379 | scaffold11401 | GGAAGAAGTGACTGTGGGGA | CGGTAAGTCTGTCGTTTTCCTC |
| 380 | scaffold47333 | GGACATATGACTCCTGAACGC | TTTTGGATTGAGAAAACTAAAACA |
| 381 | scaffold335 | GGAGACACGCCTATCTCACC | AGCTGTAATGGCTTGTTGGG |
| 382 | scaffold10339 | GGCGAGGAGGTTGTTGATTA | CTTCGACGGAGATTTTCGAG |
| 383 | scaffold60845 | GGTATGAGGTCAAAGCCCAA | AAACAGAACCAACGGACGAC |
| 384 | scaffold8422 | GGTTACATCTGGTGGGGTTG | ACCTGCAAACACACAAACCA |
| 385 | scaffold42043 | GTATGAAGGACAACGCGACA | TTAGCAGACTTTAGCGCCGT |
| 386 | scaffold27981 | GTATTCTGGCCCCACAGAAA | AGAGGAGCTAAAGCAAGGGG |
| 387 | scaffold1714 | GTCTCGGTCGTTGGAATGAT | CAGAGGGAAAGAGATCCGTG |
| 388 | scaffold59540 | GTGATTGTGCGTGTTATCCG | CCTCCATCAAGCTTCTCTGC |
| 389 | scaffold15988 | GTTAACGCGCTAGAGGGATG | GGAGAGCGCAAATACACACA |
| 390 | scaffold10077 | GTTGAAGCTCTGAAAACGGC | CGACTGCAAATTGAAAGCAA |
| 391 | scaffold1081 | GTTGTGGTCTGCCAAAAGGT | CGCAGGAAACTGTAAGAGCC |
| 392 | scaffold37565 | TACGGCCGGATTGTAGAAAG | TTCGACACTTAGCGAGAGCA |
| 393 | scaffold18695 | TAGAAATCAGAGCGAAGCCC | TTCATCAGGGATCTCCAAGG |
| 394 | scaffold38108 | TAGTGCCAACCAAGAGGAGG | CGATTCTCTCAATGCGAACA |
| 395 | scaffold57908 | TATAACTGCAATCCCGCACA | ACTTTACGCTTTTCCTCGCA |
| 396 | scaffold27392 | TATGACGTGGCATGGTTGTT | TTCGCATCACCCATACTTGA |
| 397 | scaffold14140 | TATGGCATTTGTTCCTGTGG | TTTCACTCGGGTAAAATCGC |
| 398 | scaffold15412 | TCAGAAGTGACGAAACTGCG | TTGGCAGAGGAGAGAAGGAA |
| 399 | scaffold2809 | TCCAACTAAGTCGTGGAGGC | TGCCTAATATATGTGTGTGTGTGTG |
| 400 | scaffold31641 | TCCGGGAGAAGAAAACAAAA | TTGTCGTCGACTTCTTCGTG |
| 401 | scaffold5856 | TCCGGGAGCTAGAGATCTGA | TCCTGCAAAACCAAGCATAA |
| 402 | scaffold3371 | TCCTCTCCTCCTCCTCCTTC | TGGTTACCGTCAAAACCCAT |
| 403 | scaffold24265 | TCGAAAATTCGTCATCCTCC | TGCTCATCAGGAGGAACAAA |
| 404 | scaffold15947 | TCGCCGCATAATTTAGTTCC | GAAAATGGGGTAATTGCGAA |
| 405 | scaffold12597 | TCTCAACCTCTTCCTGCGTT | CAGAACAGACTTTGCATCGC |
| 406 | scaffold58846 | TCTCCTTCCTCTTGCCTTGA | CCTTCCTGTTCCACCAACAT |
| 407 | scaffold38444 | TCTTCTCCCAAAAGGAAGCA | CACAATTCCCGTTTCCACAT |
| 408 | scaffold23505 | TCTTTCGTCACAAGAATGCG | GCAAGTTCCTCCATTCCAAA |
| 409 | scaffold25784 | TGAAAGAACCACAAAAGATTGAAA | AACAGCCCTAGGTGAATCCAT |
| 410 | scaffold1477 | TGAAGCAGAAGCTGAAGCAA | TGGCTTCGCAAAACAACTAA |
| 411 | scaffold45700 | TGAAGTGGGCAGACAGAGTG | CCCTAGCAAAAGGTCCACAA |
| 412 | scaffold1203 | TGAATCAAAAGCCCAAAATCA | CGTTGTTCTTTGTTTCAGAGAGA |
| 413 | scaffold53014 | TGAATCCAGTGTTGGTGGAA | ACCGGATGCCGTACATAGAG |
| 414 | scaffold124 | TGAATCTTTCACTTTAAACTTGCC | CCTCCACTGGCTGCTTTTTA |
| 415 | scaffold20827 | TGCATTGTCTGCTCTCTTGG | TGAGCAAGTTCCTCCACCTT |
| 416 | scaffold11771 | TGCCTACTGCCTCTGATCCT | CGCTGAAACACGAAGAGTCA |
| 417 | scaffold13402 | TGCTGATTACCAGCCCTCTT | ACCACCTCCCTGTCAAACAA |
| 418 | scaffold7304 | TGGAACCACTTCTATGCCAA | GTAATCCCGGGTTCAACAGA |
| 419 | scaffold49002 | TGGATCCGTAACATTGAGCA | TAGGAAGACGCAAGGGAAGA |
| 420 | scaffold8147 | TGGCTTCTCGGTGGAGTATC | CGAGTCAGCGTCTAGGGAAG |
| 421 | scaffold9958 | TGGGCGAGTGTGGATAGATT | TGGTGAAACATAGTTTCTTTGGG |
| 422 | scaffold5811 | TGGGGATTCGATTCTTCAGT | TTAATGTTTTTCCCCGACGA |
| 423 | scaffold14755 | TGGTCCGATGATGAGTCAAA | TGATCACAAGAGTTAATGGGATG |
| 424 | scaffold11612 | TGTACGTGCACCACGTTTTT | CTTCGATTACTCGGTGGCAT |
| 425 | scaffold3205 | TGTCCGTGGTGAATACAGGA | CCCCAAACACACACACAAGA |
| 426 | scaffold7679 | TGTGATCATCTCCCCAAACA | TGAACAAGACCTTCAGGTGG |
| 427 | scaffold27147 | TGTGGAGAAGGGAAACGAAG | TTGAGAGTTTCAGCATCAAAGC |
| 428 | scaffold38361 | TTAAAGAAAGGCAAATGCGG | CAAGGCGAATCAGTCAGACA |
| 429 | scaffold3606 | TTAGACGAACAAACCCGACC | ATCTCGGAAACCACTCATCG |
| 430 | scaffold17728 | TTGCTATTTCGCTATTCCAGTG | CTCGCTATACGGAACCCAAA |
| 431 | scaffold13627 | TTGTCAAGTGCCAACAGATG | ACCGACTACACCACCCAGAC |
| 432 | scaffold22831 | TTTCGGTTTAACTCCGGTTG | TTTCTGTGCGGGTTTGAAGT |
| 433 | scaffold42556 | TTTGATCATGTCCGGTGCTA | ATGGTGGGTAGATCAGACCG |
| 434 | scaffold15497 | TTTGCCACCAAAACACTCAA | CAAACCAGCACGAATCAATG |
| 435 | scaffold2710 | TTTGGAAGTTGTTCCTTGTCTC | TCCTAGCTTCTGGGGCTGTA |
| 436 | scaffold24088 | TTTGGTTGGATCGTTGTTCA | TCCAAGGCAAAGAACGAAAC |
| 437 | scaffold21408 | TTTGTTGGTTTCATGCCAAA | TCGGTCATCTGCAATGGTAA |
| 438 | scaffold13221 | TTTTCCCTTCGTCTCCATTG | CGTTGTTCCTGTGTCTCCAA |
| 439 | scaffold11286 | TTTTGGATGAAGCCCAAGAC | GCTAATCAGATCTCTCGCGG |
| 440 | scaffold12485 | TTTTTCTCGCTTCAATTCCG | GGGAGACAAAGAGCGATGAC |
| 441 | scaffold21110 | TGTCGACAGAGGATGAGGAA | AAACCTATGCAATCTCAACACG |
| 442 | scaffold3644 | TAGCTTAGGGCCGCTTTTCT | TTTTTGGTCAAATGCAAACG |
| 443 | scaffold50184 | TGACCGAGACACTCCATCAA | GCGGTTAACAACTTGGGAAA |
| 444 | scaffold2675 | AACCCCTAGCCGTCAAGATT | CGAGACACCTCACCTTAGCC |
| 445 | scaffold22567 | CAAACAGGCCCGTCTCTAAG | AGGTGGAGCTGCATTTGATT |
| 446 | scaffold23518 | AAAAACCACAATCTCCGCTG | GTGCAAGTGGACTCAAAGCA |
| 447 | scaffold3635 | AAAAGCAGCAGACTAGCCCA | AGCAATTGCGTCATCATCAG |
| 448 | scaffold9482 | AACGGACAAGGTTGCTTCTG | AGCCTTTGGTTTCTACGGGT |
| 449 | scaffold59817 | AACTACGTTCCCCCATTTCC | CTCCCATGGCTTCTTGATGT |
| 450 | scaffold7219 | AAGAGGCACAAGAACCGTGT | TTCGATTCACGGATGGAAAT |
| 451 | scaffold24235 | AAGCTCAGCCACGTCCTAAA | GCAAAGATTGGTGGGTGAGT |
| 452 | scaffold2245 | AATTAAAAGAAAACATCGACAAAAA | CCTCGATATCCCTCCTGTGA |
| 453 | scaffold13125 | ACAGTTCGCCTGATGAGCTT | AACACAGAGCTGAAACGCCT |
| 454 | scaffold1132 | ACGCTCTCTCTTCGGTTACG | CACCAAAGGAGAACCGAATC |
| 455 | scaffold23778 | ACTTGTGCGTGCATTGACTC | TCTCAGCTTCGGCGATACTT |
| 456 | scaffold37061 | AGAAGAAAGCTCTCAAGCCG | GAAGAAGGGAAGGAGCGAGT |
| 457 | scaffold3082 | AGCGGAAGTAGCCATAAGCA | TGATAAAGATCTCCTCTCCCCA |
| 458 | scaffold16291 | AGGAACCGTCTTTTTCGCTT | GAGCTGCCGCCATATAGTTC |
| 459 | scaffold46224 | AGGGACACTTCACTCGGTTG | AAAAACACGAACACGAGATGT |
| 460 | scaffold6817 | ATATTTCCCTCGTTCGCTCC | AAAGCAACCACCCAGAATTG |
| 461 | scaffold6048 | ATCAACCGCCTACACTCCAC | CCGTTGCAGATCCTCCTCTA |
| 462 | scaffold1392 | ATCGTCTGAGCTCGAATCGT | CGAAATTTTATAAGCCCGCA |
| 463 | scaffold11869 | ATCGTCTTCTTCGTCGTCGT | ACAGCTAGTTTTGGGCTGGA |
| 464 | scaffold14885 | ATCTCTACATGGACACCGGC | ACACGGTCTCGAACTGGAAG |
| 465 | scaffold17018 | ATGGAACCACCTTTGAGCAG | GGGCTGCAAAGAGAGAGAAA |
| 466 | scaffold419 | ATGGCCCAAGAGACATCAAG | TCGGGAAGAGAAGAGAACCA |
| 467 | scaffold61158 | ATTCGTTGACCCTTCCCTGT | TTTCTTTGCGAGTTTGGCTT |
| 468 | scaffold27478 | ATTTGCTTGGATCCGTGAAG | CCGAGCAAACAGAGACAACA |
| 469 | scaffold50376 | CAACCAGTGAGATAACTTGTTTAGC | CAACCACCATGAACAACCAA |
| 470 | scaffold10039 | CAATCGATGCGCAAGTTAAA | TGAAATAACGTGAACCCCGT |
| 471 | scaffold19059 | CAATTCACCCCCAAAAGAAA | TTTAGTTCCGAAGTGCGTGA |
| 472 | scaffold56821 | CACGTCATCATCACCCAAAG | GAAATCGGAAAGCTAGCGAA |
| 473 | scaffold19141 | CACTCCCACCACCACCAC | AACCTTGCCTCCCCAACTAT |
| 474 | scaffold13533 | CACTCGTGGACGAGACAAAA | GTACCCGGAAAGAAAGGAGG |
| 475 | scaffold7147 | CACTTGTGTCCGAAAATCCC | CAGATCGCCACGTAGCTGTA |
| 476 | scaffold11244 | CCAAAATTTGCATGAAAGCC | CAGCTTGTGCTTGTCTTCTTG |
| 477 | scaffold61519 | CCAAACGGTCATTTCGTCTT | CAGGAGGAAGGAATCGATGA |
| 478 | scaffold6894 | CCAACATAGCCACGAATCCT | CGTTTCTCCCAGCAGCTTTA |
| 479 | scaffold14131 | CCATTGTTGGTCGTGTTGAG | TTTGCTTTCGATCTCAGGCT |
| 480 | scaffold24765 | CCCACCAGCTTTTCGAATTT | TGGAGATGAGCTCGATGATG |
| 481 | scaffold50429 | CCCACCCTGAAAAGATAGCA | GACACCCCTCAAATCAAACG |
| 482 | scaffold37036 | CCCTTAAAGCCTGTCGAGGT | ATTCGATCCACTGTTCGAGG |
| 483 | scaffold26184 | CCTAGAGCGAAGCTCTGCAA | TTGTTAGGGATCTCGATGGC |
| 484 | scaffold45004 | CGAATCATTGATCCCATTCC | GGACAAGAACCCACCAAGAA |
| 485 | scaffold11606 | CGAGAGAGATCCATTGCCTC | CTCCTGTCATTTCCTGCACA |
| 486 | scaffold5913 | CGAGTCGGCATTAAAACCAT | AAAGAGAGCTGCTTTTCCCC |
| 487 | scaffold20346 | CGGATGATTACTACGCCGTT | TTCTTGATCTCCTCTGGCGT |
| 488 | scaffold9352 | CGGCTTACAAGTCTCTTCCG | GCACACCAGAAAACCCCTAA |
| 489 | scaffold1127 | CGGCTTGACAGAATTGGACT | TCCTATTCCACACCAAAGCC |
| 490 | scaffold52575 | CGGGGAATGAAATTTACCCT | TGGAAATTGGGATGAAATCTG |
| 491 | scaffold669 | CGGTCCAGTTTCCAAAACAT | ATCGACGCACAATCCAAAAT |
| 492 | scaffold53056 | CGTATGGCGCGTGAATAGTA | GGAGAAAACTCTCTGGCTGG |
| 493 | scaffold9116 | CGTGACACATTAGTCAAGCCA | GCAGCTGCTTCTTGTACGG |
| 494 | scaffold1421 | CGTTTCGAGTGTCTCACCAA | TCGAGGCTAGGTTTTGCAGT |
| 495 | scaffold17419 | CTAATAAACCCCTCCTCCGC | ATTTTGGTTTTTGCTGGTGG |
| 496 | scaffold13002 | CTAGGCTGAGAAAACCGACG | GGATCACAAAGCCTAGCCAA |
| 497 | scaffold5924 | CTCATGGCTGCAAGTGTCTC | GTGCCTCGTTGAGTTCCTGT |
| 498 | scaffold18963 | CTCCTCCGCATCTATTCGAC | AGAAGCCTCGAGTGGCAGTA |
| 499 | scaffold9774 | CTGAATAAACCAAACCCGCA | ACAAAGAGAGGCGATGGAAA |
| 500 | scaffold21727 | CTGGGAGAAAAGATCAAGCG | GAATCCAGCAAAGGAATCCA |
| 501 | scaffold59860 | GAAAACCTTGGGGAATCGTT | CAACCCCATTGTGAAAACCT |
| 502 | scaffold17758 | GAAAATCCTGCAAACCCTGA | CTCTCCATATCTCCGGACCA |
| 503 | scaffold24936 | GAGATTGGAAGAGCGCAAAC | ACCCCAAGCCTGGTTAGACT |
| 504 | scaffold1201 | GAGCTCAAGCTCTGCGTCTT | AACTTGGGCCTGGTTCTTTT |
| 505 | scaffold6440 | GAGGAGAGGGTGTGATCGAG | AAAGCCATTGCAGCATTTCT |
| 506 | scaffold6272 | GAGGCAATTTTGTCTATCCCA | CGACGATGGATAAGAGGGAA |
| 507 | scaffold45700 | GATGCTGATGGTGAGCGTAA | TGAGAGCAGTCCATTGCTTG |
| 508 | scaffold26074 | GATTCCAGCGAGGACGATTA | CTTCCTTCTCGACCAACTCG |
| 509 | scaffold5 | GCAAGAATCTCTCATTCCCG | AAAAACCCAAAAAGGACGAA |
| 510 | scaffold893 | GCAAGATGATGGGGATGACT | CATGCCACAAATGTCTCCAG |
| 511 | scaffold14212 | GCAAGGGGGAGAGAGAAAAC | AACGGTGGCGTATCGTAAAG |
| 512 | scaffold45004 | GCACCTTGGGATTGGTTAGA | GCCACATTTTAGACCTTGCC |
| 513 | scaffold10238 | GCACGTTCTCTTCGTTCCTC | GCGTGAGGCTCATCAGTGTA |
| 514 | scaffold38160 | GCCCTAAACATGGCATTGTC | GGGTTTCTTCAAACACCAGAA |
| 515 | scaffold2218 | GCCTCATCACCACAGTTCATT | GGGTGGATTTTGTGAAGGTG |
| 516 | scaffold46621 | GCGTAAGCGTTTGAGAGGTC | AGAGGAAGAAACGATTCGCA |
| 517 | scaffold1421 | GCTTCCTTCCTTCCTCTCGT | AGGAGCCATGACTTTGATGC |
| 518 | scaffold6486 | GCTTTCTCAGAGGTGCGTTC | CACTTCCGTAGGAGGTGCAT |
| 519 | scaffold7066 | GGAAAATGAAAGACGGTTTCC | AGAGAGACGGCCCAAACCTA |
| 520 | scaffold959 | GGAGAGAGAGCAAACATCGC | AGGACCTGGAAACATCGAGA |
| 521 | scaffold33939 | GGAGAGAGCGTCTTTGGTTG | TTAAAGCCACTTAAGGCCCA |
| 522 | scaffold17769 | GGAGTGGGGATCACGTTAGA | CTTTCGTGTCCTGGTGGAAT |
| 523 | scaffold60283 | GGATCCCAATTGGTTCTTCA | TGGATTTCGTTTTATGCTTTCA |
| 524 | scaffold41944 | GGGAATGGGAGAAGAGAAGG | TCAATTTTGACTTCGAGCCC |
| 525 | scaffold7897 | GGGCCTGCTTTTGTCTCTTT | AAATGCCTACGTTTGTTTCCA |
| 526 | scaffold9141 | GGGTTCCTGAGTTTAGGACAGA | TAAAGTGGGTATGGCCTCGT |
| 527 | scaffold3967 | GTGCAAGAGGTATTCGCCAG | CATTACGTCCAGCATTCCCT |
| 528 | scaffold28816 | GTGCCGAGGCATGATTTATT | CAGATTAGCATTGCGGTGAA |
| 529 | scaffold19982 | GTGGTTGGAAATATCGACGC | TGAAGCTCACAAATCGCAAG |
| 530 | scaffold10580 | TACCGTTGACCAAATACGCA | AGGGGAAAACGAGAAAGCTC |
| 531 | scaffold43458 | TACGCCGCCTCAAAGATAAC | ACATTCGTTTCCAAACTCCG |
| 532 | scaffold4145 | TATAAAAGGCGAAGACGCCA | GAGGAGACTAACTCATCAAAAGGC |
| 533 | scaffold5148 | TATCTTCTTCCTCGCCGAAA | AAGAAATGCCAAAGCTCGAA |
| 534 | scaffold12074 | TCAAACCTTCGCACGGTACT | TTTTGCGGAAGAAACAAAAGA |
| 535 | scaffold6711 | TCCAACCCCATGGAATCTTA | AGAGAACGTCCCTCTACGCA |
| 536 | scaffold12008 | TCCACGCTTCGTCTTTCTCT | AAGAAATGGGGGAACAGAGG |
| 537 | scaffold32915 | TCCTCAGCCACCAAGGTTAC | CGATGAAAAGCTTTAAACGGA |
| 538 | scaffold30255 | TCTCTGCCTAACAGTCCCCA | GAACCTTCAAGTCGAGACGC |
| 539 | scaffold10390 | TGAGGAGATGAACGAACGGT | TTGCAACATTTTTGGAGTGAA |
| 540 | scaffold10556 | TGCAGCTCTGAACACAAAGG | ACAGACCCAAAACGCTCAAC |
| 541 | scaffold52751 | TGCATCATTGCAGTTCTTGA | CGCTAAGTCTAACCCGACCA |
| 542 | scaffold2382 | TGCGAAGTATGCTTTGTTGC | ATCCACGCCGTTTTTACAAG |
| 543 | scaffold940 | TGGATTGGTACAGACGACGA | AAAGCCAAACAAAAAGCCTG |
| 544 | scaffold47235 | TGGGGGTGGATACAGGTAAA | CAAAAAGGACAGACCCGAGA |
| 545 | scaffold39782 | TGGGTCCCAGTTACAAGAGG | GATCGACGGCCATTATGATT |
| 546 | scaffold2207 | TGTTGCTCACGTCTTGAAGG | AACACCAACCTCAGCTGGTC |
| 547 | scaffold6508 | TTAATTCCTTTTCGCCATCG | TTTTCTATTTTCCCCCGACC |
| 548 | scaffold40573 | TTCACAAAATTAATCCCCGC | TCTTCCTGAGCGCCATATCT |
| 549 | scaffold15044 | TTCACTGGAGATACGCATGG | GATTCCCCCACTATTCGTCA |
| 550 | scaffold9809 | TTCCCAATCTGCATCTCCTC | AACGATTTCGCTGAAGCCTA |
| 551 | scaffold43712 | TTGACCAAGCACTCATCTTGA | GCTCTGCCCGTTGAGAAATA |
| 552 | scaffold16490 | TTTCAACCGTCCGATCTTTC | CAACCTAAGAGAACGGCTCG |
| 553 | scaffold6828 | TTTGGTAAGCTGCTAAGCTCG | CAAAGAACTGGGTCTGCCTC |
| 554 | scaffold30044 | TTTTACCCCAACACGTTGCT | AACGAAGCATCATCGATTCA |
| 555 | scaffold2331 | CCGAATCTTCCATTTACCGA | CCATGTCCATCTTCGCTTTT |
| 556 | scaffold2426 | TAGCTAGACGCGGACCCTAA | TTGCACCACCGTTACCTTTT |
| 557 | scaffold1468 | CAATGCTCCGCCTTCAAA | TTTGAGGTTTCTCTCCAGGC |
| 558 | scaffold16291 | TTGCACGTACGTCTTTGAGG | CTGCAACGAGGATGAAAACA |
| 559 | scaffold195 | AAAAACACTTAGTTGTGGTGGGA | TTTTTAATGCAGCCCGAAAC |
| 560 | scaffold13074 | AACTAGGGTTTCCTAGCCGC | CGGGTTAATCTCCAGTTCAAA |
| 561 | scaffold7739 | AACTGATATCCGAACGCCAC | TTCATCATCATCGGACCAAA |
| 562 | scaffold15880 | ACACAGCCTTCCTAACCGTG | AAGACGATTTCACCCACGTC |
| 563 | scaffold13693 | ACGACGTTTGTTGCGTTGTA | GGGTGGGCATCTTTTGACTA |
| 564 | scaffold28218 | ACGCCAAAAGTTCACGTTCT | CAACCCACTGCTCAAACAAA |
| 565 | scaffold47080 | ACTCCACATTCTCCGACGAC | CCCCAACTAGTGGCGTTTTA |
| 566 | scaffold22377 | ACTTTGCTCCGACAGGTCAC | CTTCTTCGTGTTTCGCACAA |
| 567 | scaffold20811 | AGAAGGAGAAAGCAGGAGGG | CGCAATCCAGACTCAAACAA |
| 568 | scaffold9521 | AGCACCGCCGATGACTATAC | TGAAGCGAGAGGACGAATCT |
| 569 | scaffold12680 | AGCAGGAACAGCTCGAACAT | CTGGTGTCCCAACTGTTGAA |
| 570 | scaffold9176 | AGCCTGCATCCAATGTTTGT | AGGAGGAGGATGATCGGAGT |
| 571 | scaffold37773 | AGCGCTTCAGTTTCGTGATT | AAAATCCGAAATACCCGACC |
| 572 | scaffold3618 | AGGGAATTACACCGTCATAGAA | GGCCCAATGCTAGAGAGAGA |
| 573 | scaffold773 | ATCCCTGGAGACACGAAATG | AAATGTCTGCTCCTCCTCCA |
| 574 | scaffold5007 | ATCTTCCTCCACGTTCATGC | TGAGGAGTGTGTGTGTGTGTG |
| 575 | scaffold1068 | ATTGGCGGTAAACCACTGAG | CCGTTTTCTCTGCTTCCAAC |
| 576 | scaffold63922 | CAAAAACTAATCCAAAAACCACAA | AGCATCTTGCCGTTTCCTTA |
| 577 | scaffold1869 | CAATTCCACAAGCGTCTCCT | CCGGCGATACTCTTTGTTGT |
| 578 | scaffold18618 | CAGACTCTTTGACCGCCATT | GCTCCGGTCAGATCCATAAA |
| 579 | scaffold17856 | CAGGTCACTACAAGTGGGCA | TCCCTATCCATGAGACGAGC |
| 580 | scaffold3267 | CAGTCAATACTCGCCGAACA | TGGCCAAGATTGTGTTTCTG |
| 581 | scaffold24371 | CATCTGCATCCTCCTTGGTT | TTGTACCAGTTGTACCTTCGTGA |
| 582 | scaffold37003 | CATTTTGGCAGGGATCATTT | TTCTTCTGTGTTCGCCCATT |
| 583 | scaffold40135 | CCAAAACTTAAACCCAGGAGG | TAGAGAGAGTCGGCCACGAT |
| 584 | scaffold35034 | CCAAATGGTCACGCCTACTT | TCTCGGCTGATCTCTTCCTC |
| 585 | scaffold40920 | CCACGTCCCTAAGGATCAAG | CTTGCCGGAGAGAGAGCTAA |
| 586 | scaffold12712 | CCATCCCACCAACTCACATC | TTGAAGATTCAAACCTTTGGTG |
| 587 | scaffold7700 | CCATCTCGTCAGAGCTTTCC | GGTAGTTCGGAGCTGAATCG |
| 588 | scaffold49505 | CCGGAGACATCCACTGTTTT | CCTCCACCAACCATGATTTC |
| 589 | scaffold5233 | CCTTAGAAACAGAGCCGATGA | TGGACACAAGTATACCCACCC |
| 590 | scaffold10748 | CCTTCTCATACCTCTCCCCC | CCGTGAACGTCATTGATCTG |
| 591 | scaffold63554 | CGAAGGGTGCAAGAGAAGAG | CGAGATACAAATGGTCCGGT |
| 592 | scaffold11260 | CGAGAGAATCGGAGGTCAAG | TCATCGTCCACAACTCCAGA |
| 593 | scaffold10921 | CGAGTTCAGACATTGAAACCAA | GCAGAAACTGCTTCCTTGCT |
| 594 | scaffold14044 | CGATCCTCCCAACAACATTT | CTTGGCAAGGGACCTATCAG |
| 595 | scaffold3912 | CGTCGTCGTCTGCTCAATTA | TCATCAGCTTCGTCGTCAAC |
| 596 | scaffold5047 | CGTTTCTGCGAGTGAGTTTG | GGAAACCCTGAAAGTCCTCC |
| 597 | scaffold11925 | CTAGTGTTCGCCGAAGTGGT | AAAAGGTGTCATGGAGTGCC |
| 598 | scaffold21910 | CTATCTTCCCTCGTTGCTCC | CGCAAAACCAACAATTGAGA |
| 599 | scaffold4225 | CTCGGTTTTGTTAGGGACCA | CGTACCTGATTGCCACCTTC |
| 600 | scaffold33948 | CTGAAGCTTCCGGGTCTATG | GCCGTGGATTTATTCGCTTA |
| 601 | scaffold12073 | CTGTGATGCTCACATCGGTC | AGCTGTATGTCTCCCGTGCT |
| 602 | scaffold22798 | CTTCGGTTTCAACCGTCATT | ACAACACTCCCTGCAAAACC |
| 603 | scaffold14838 | GAAAAATGCTCGAAAACATTCAT | AGTGCGATAGCTTGGGAATG |
| 604 | scaffold6173 | GAACTTCGCGGCAGTTTATC | GATCGGGTGGCTCATTCTTA |
| 605 | scaffold11956 | GAATGTTTCCTCTGCTTGGC | TCAAAATCAGGAGAATCGGG |
| 606 | scaffold2657 | GACAGTCATTTTCCGTCGGT | GAGAGAGAGAGGGAGGGAGG |
| 607 | scaffold5823 | GACTCTGCCATTCCGTCAAT | CTGGTTCACGAAGTCTGGTG |
| 608 | scaffold997 | GAGCTTTTTGAACTCACGCC | TGTTTCTGGTAGATTCCCCG |
| 609 | scaffold47859 | GAGTGAAAGGATTCGGTCCC | TGGGTTTCTGGGAACTTACG |
| 610 | scaffold32391 | GATCGCAGTGGATTGTTTCA | GCAACGCTGAATGATGAAGA |
| 611 | scaffold6648 | GATCTTCCCTGACGAGAACG | CATTCAGAGCCTTACGAGCC |
| 612 | scaffold22209 | GCAACCAGATTTCGTTTCGT | CGATTGGTGTTCCCTCACTT |
| 613 | scaffold7315 | GCCGTTTCTCTTCTCACCTG | CAAAGCCAGTCGAACCTCTC |
| 614 | scaffold18962 | GCCTGCTTCTCTTCAACACC | ACTGCTGCTTCCTGCTGATT |
| 615 | scaffold8156 | GCGAAGCTTCCTTTGTCATC | ATCTGAGACGGAGCGTGAGT |
| 616 | scaffold2604 | GCTAGAATCGCCTGATCGAC | GCGCGGATGTTTGTTAATGT |
| 617 | scaffold827 | GCTTCATCTTCGGCATCATT | AGAAGTCTGAGGAGGTGGCA |
| 618 | scaffold21632 | GGAAGAAACGCTTGTACCCA | CGTGGTTCGTGGTAAGAGGT |
| 619 | scaffold17589 | GGACATGTTGTCACCAGACG | ACGATGGCAGCACTTAGGAT |
| 620 | scaffold6901 | GGCGAGAGTGTAGACGAAGG | GTGTGTTGCCCTTTGTTCCT |
| 621 | scaffold16441 | GGCGGCGCTGAATAAAAA | TCTTCCTGGTTTGCCACTTT |
| 622 | scaffold8814 | GGTGAGAGCTTTGGTTACGC | CAAGAACCGCTTACCTTTCG |
| 623 | scaffold5580 | GTATTGCATTCACGCCCTTT | ATCCTCCCTCTCTGGGAAAA |
| 624 | scaffold5425 | GTCCGTCGTTTTGAGTGGTT | TATCCAAAACGGCGTCTCTC |
| 625 | scaffold9590 | GTGAGCTTGCAAATCGATGA | GCCATCGAACTTCTCTGAGG |
| 626 | scaffold22739 | GTGCACGTGGAGCATGTAAC | ACAACAGCTTCCGCTTCAAT |
| 627 | scaffold30104 | GTGTTTTTGCTTCGTCGTCA | CCCCTTTTCGATTCCTATCC |
| 628 | scaffold2057 | TATATAAACGCGCATGCCAA | ACCGGTAAAAGGGGATTCAG |
| 629 | scaffold17322 | TCAGAATCTAATAAGGAAAATGCAA | GCATGTGGGCTCTTTTTCTG |
| 630 | scaffold21043 | TCAGATTCCGTTTCGAGCTT | CACAGATCACGATCGAGGAA |
| 631 | scaffold42394 | TCCCACACTCTCCAGTCTCC | CCCAAGTGAAGCTGTCGAAT |
| 632 | scaffold52127 | TCCCAGTATATCGTCGCCTC | CACATTTCGCACCAATATTAAGAA |
| 633 | scaffold6648 | TCCCCTCTTCTTCATCCCTT | AAACCAAAACAGAGGGTTGG |
| 634 | scaffold22180 | TCCCTCAACATACCCATTGA | TGTCCATGACCATTGCTTGT |
| 635 | scaffold30337 | TCGAATGCCATATCACAGGA | ACCACCATAGCCAAAACCAA |
| 636 | scaffold26088 | TCGAATGGTTGTTTGTTGTTG | GCGAGACGTATTGTTTGGGT |
| 637 | scaffold32549 | TCGATCTAGACGACCAGGCT | AAACTCGACCCCGCTTTAAT |
| 638 | scaffold12874 | TCTCTCTTCTCTCACGTTCTTCA | GAGGTGATGGCTGAAACACA |
| 639 | scaffold47700 | TCTTCCTCCTCCCTCCATTT | ACGTAAACCTGAGGAGCGAA |
| 640 | scaffold44547 | TGACAAGTTCGCTCCTGATG | CACAACCTGCAAACATCCAC |
| 641 | scaffold30407 | TGATGAGTCAAGGGGGAAAG | TTCCTCCCATAAAGATCCCC |
| 642 | scaffold42379 | TGCCAATTCGCAAATAACAA | CCCTAAAAGTCCGGAAGGAG |
| 643 | scaffold7769 | TGCGATATTAATTTGGGGGA | CATGTCCAGATGCGATTTTG |
| 644 | scaffold967 | TGGAAATAGGCATATTACATTGG | GAAGAGCAGGAAGTTTTGGC |
| 645 | scaffold64091 | TGGATCCAAACCTGATACGG | CTTGATATTGCCGATGGTGA |
| 646 | scaffold4722 | TGTTGATGGATCAGCATCGT | GAGATCTGCACACAGCTGGA |
| 647 | C18426353 | TTCAAGGAGACGCTACAGCA | CTTGAAATCCCGCTGATGTT |
| 648 | scaffold18344 | TTCACAAGGCTTCTCAGCAA | CTGATCTTGGAATGCCGTTT |
| 649 | scaffold1947 | TTCCAAAGGAATCGGGATAG | TGACTCTGCTCCGGATTTTC |
| 650 | scaffold48612 | TTCGAATCCGCCACTTAATC | CGCGAATTTCTAGGGTTTTG |
| 651 | scaffold29058 | TTCGCTCCTATGCCCATATC | AGGCAAGCACTTCTGACCAT |
| 652 | scaffold14184 | TTCTCCTTGCAAATTCCGAC | TTCTTCTCCAGCGTCCTTGT |
| 653 | scaffold49160 | TTCTCTGCGTTTTCAGGTGAT | CCTAAGAGCCGTGACATGGT |
| 654 | scaffold16039 | TTCTGGACGATCTGTTGCTG | CGCCAGATCGATCCTTCTTA |
| 655 | scaffold40413 | TTGAAGTCGTGAGAGTGGGA | CAAAAGGTTTCTCTCCTGCG |
| 656 | scaffold9796 | TTTCATGCGTGGATTGAGAA | ACTCATCCGGTTTGAGAAGC |
| 657 | scaffold6333 | TTTCTTTCTCAGTCGCGCTT | GTTGGATGACGACATGATGC |
| 658 | scaffold21825 | TTTCTTTTTCTCCAAGTCGCA | TAATCGGTCCGGTCCATAAC |
| 659 | scaffold10683 | TTTGAAACGTAAAACCCCAGA | GGGGTGTTATTCAATGGTGG |
| 660 | scaffold10174 | TTTGTGGGCTTTTTACTGGG | TTGAACATCGGATCGAATCA |
| 661 | scaffold19258 | CCTGGAAGAAAGGAGAGGGT | CGGTGCAAAGAAGCAAATCT |
| 662 | scaffold4206 | AAAACAAAATCAGTCTATATCGAAAGT | AGAGGGCTTTGGCAACAAT |
| 663 | scaffold2407 | AAAACATTGGGGCAAATCAA | GCGAGACGTATTGTTTGGGT |
| 664 | scaffold4220 | AAAACCCCACTTTTGACGTG | CCGTCGTTATCATCCTCGAT |
| 665 | scaffold7082 | AAAGCAATCCCCACAAATCA | CACGCAAGATTTGGCTTCTT |
| 666 | scaffold14164 | AAATCCAAACCCACAACCAA | CCCATCACAGAGGCAAGAGT |
| 667 | scaffold20706 | AACACACCGAAGCTGTTCTT | TGCGGACAACGAAACTAACA |
| 668 | scaffold8882 | AACGTAAAACGAAGTCGCGT | TCTTCTACGAGGGGATTCCA |
| 669 | scaffold29426 | AACTTGATGTGAAAACCGGC | GGTGGCATAAGCATTGGTCT |
| 670 | scaffold13471 | AAGTCTAAGCATTGACGGTCG | CAAAGCTAATGGGAGAGCCA |
| 671 | scaffold7958 | AAGTTTAGAGTTTAGGGTTTAAGGTTT | TTGATTTTGCAAAGAGAAATACAAA |
| 672 | scaffold354 | AAGTTTCGGTTAAAGCCGGT | CCCTCACTGCTCAACTCCTC |
| 673 | scaffold27217 | AATAGAAACCACACCGCACC | CATGAAAATTCCAATGGGGT |
| 674 | scaffold23238 | AATGCCGCGTTAATCTCATC | CGGAGTCGCCACATATCTTT |
| 675 | scaffold7128 | ACAACCACCACCCACTTCTC | TGCTTGACAGCTTTGCTCTG |
| 676 | scaffold29145 | ACAACGTCGCTTAAATTGGG | CGAGGAGAGCGAAAGAGAGA |
| 677 | scaffold12597 | ACACTGTGGGATGGATGGAT | CCGGTCTGTTTTTCGGTTTA |
| 678 | scaffold19852 | ACATCTCACGTGGGAAAAGC | GCCATGAGAGGGTGAGAGAG |
| 679 | scaffold9474 | ACATGGTCATGATGGTCTGAA | GGCCGCATTTTAGGCTATTT |
| 680 | scaffold10574 | ACCAATTAGCATCTCGCCAT | CTTTCATTTAACACCCCCGA |
| 681 | scaffold18036 | ACCAGCCCATTAGCCTTCTT | CAAGTGGCTCTTGATGTCCA |
| 682 | scaffold917 | ACCCAAACCACTGCTGCTAC | GGGTCACACTTTGCTCGTTT |
| 683 | scaffold9872 | ACGATTCCAATCACAAAGGC | GTCGTCGTTGCTCTCCTTCT |
| 684 | scaffold13251 | ACGTCAGAGGCGTGAAGAGT | CCAGTTCCAGCAAAACCCTA |
| 685 | scaffold63333 | ACGTTACTCGGATCGCTGAC | TTCTCGATCGGAGATTTTCAA |
| 686 | scaffold19894 | ACTCTTGCTCGTCCATTGCT | GAGGCGCCGTACATTAGAAG |
| 687 | scaffold7889 | ACTGGTGCCCATTGTTCTTC | AGACAGGAAGGAGGTGGTTG |
| 688 | scaffold19245 | AGAAGTGACCAGCGGAGGTA | CCAATTGGGTTCCATCTCAT |
| 689 | scaffold1811 | AGAATTCGCAATGGATCTGG | AAGATTGATTTGGCGTCGTC |
| 690 | scaffold39351 | AGCCACCCAAGTTCCTTTCT | TTTAAGCTTTGCGACGAAGG |
| 691 | scaffold18334 | AGCTCACGAATCTGCTCCAT | CCGAAACCAAGATAGGTGGA |
| 692 | scaffold20705 | AGGCACACTTGTCCTTCGTT | GATGTGGTGGAGGAGCTGAT |
| 693 | scaffold11771 | AGGCCACTTTTTGTCACCAG | CATCATCTTTGGGCTACGCT |
| 694 | scaffold12754 | AGGTTTCTGAATTGGTTGCG | GCGCCTGTGCTAAGAGAATC |
| 695 | scaffold32139 | AGTCCGTTCTCATGCTTGCT | GCTTGAGATCTCGGTGAAGG |
| 696 | scaffold19775 | AGTGGGTGAACCAAGGACAA | AAGCAAGTTTCAGCCAAAGC |
| 697 | scaffold17359 | ATCAACACGTGTCACCTCCA | CACGAGAATGCCACTGAGAA |
| 698 | scaffold32 | ATCCTCCTCGAACACCTTCA | AGGGTTCCCATTAATTTCGG |
| 699 | scaffold443 | ATCGGCTTTCTATTTCCGCT | GATGTGTCGTTTTGGTCGTG |
| 700 | scaffold773 | ATCGTCTGCGAGTCTCCTTG | TGATTATGCAAACTCTCATTGATT |
| 701 | scaffold52698 | ATGCATCTTAGCTGGCACAT | TTTCTCGCCAGAATCGAACT |
| 702 | scaffold8457 | CAAATCGGCTTCCACAAAAT | GGCGATGTTACTGGTTTTGC |
| 703 | scaffold44361 | CAACGAAGATGCTTGTGGTG | GCTTGATCATCATTACGCGA |
| 704 | scaffold30588 | CAATTGGGCTGGATTCAAGT | GTGAGGCTTGAAGTTCTCCG |
| 705 | scaffold4137 | CACACATGGTGGCTTTCTTG | GATTTCCCGACAAAGTTCCA |
| 706 | scaffold37448 | CACCACTCCGCTTCTTCTTC | GGGCATGGTGTTCGATCTAT |
| 707 | scaffold22027 | CACCGTTTTCAACGGAAACT | TTGTTCTTCGAGGGTTGTCC |
| 708 | scaffold4141 | CACTTGAAACCTCCATGGGT | TCGTGCGATAACAGTGAGAAA |
| 709 | scaffold3954 | CACTTGGAGTGTGCAGCTTT | AGATTCGAGTCCCCATCTCA |
| 710 | scaffold3610 | CAGAGATCGGGATTTGATCG | AAAGCTGGGAGAGCTGTTGA |
| 711 | scaffold40045 | CATTACCCCCGAGAAGATCA | CGCATAGTTACCAATTACCACC |
| 712 | scaffold22459 | CCAACTCCGGTCAATCATCT | GCCCCTTTCTGTGTGACATT |
| 713 | scaffold5837 | CCACACAACTTTGGAAGCAA | GTGTTCAAGAGCTTGGGAGC |
| 714 | scaffold12761 | CCCCTGAAAAAGTGAAAAGG | ACTTGCATGCTTAAAACAGGA |
| 715 | scaffold39817 | CCCTAAGCTAAAACCTCGCC | ACATGTGCCATTTTGGGATT |
| 716 | scaffold10009 | CCCTTCACTCGTTTCGTTTC | TTCCTCCACCTTGACGAAAG |
| 717 | scaffold27926 | CCTCTCTCTCGCTCCATCAG | AGCAACAAATCCAGATCGCT |
| 718 | scaffold43197 | CCTGCTTCAGCACAGATCAA | AGAACTCGGAAGCCCTGTTT |
| 719 | scaffold17525 | CGAAACTCCGCATTTCCTTA | CTCAATTTCAATGGCGTCAA |
| 720 | scaffold17210 | CGAGCTCGCAGATTCTCTCT | ACGCGAGGGTAAAAGAGTCA |
| 721 | scaffold23189 | CGATCAGTACCAGCCGAAAT | TCTCTCAGCTCCTCAACCGT |
| 722 | scaffold23986 | CGCAGCTGCTTCTTCTTTTT | AGAATCCATTACCAGCGTCG |
| 723 | scaffold17198 | CGCCTAGCCTCTCAAGTCAC | TTCAGAGACCTTGGATTCGG |
| 724 | scaffold2507 | CGGGGAAAGAAAGATCAACA | AATGCTAGGAGCTGCATCGT |
| 725 | scaffold1845 | CGTCCTTCAACCTTTTTGGA | CCATGTTTGGTCTGGATATGG |
| 726 | scaffold60692 | CGTCTTTTCTCCTTCTCCCC | ACCGTTTATGCCGTTGAAAG |
| 727 | scaffold18376 | CTACGGAGACACGCAGATGA | CCTCGGAAAATGTGCCTAAA |
| 728 | scaffold3552 | CTCACGAGCTATTGATCGCA | CACGAATGATGAGCCAAGAA |
| 729 | scaffold25181 | CTGCCTGAGCAAAGAATGGT | ACACTCAAGCTGGCTGTTCA |
| 730 | scaffold48322 | CTGTTTCTGAGAGACCTGGGA | ATGATCCGGTTTCGATCAAG |
| 731 | scaffold22831 | CTTCAAACCCGCACAGAAAC | TTGAACTCCGCTCTTCTCGT |
| 732 | scaffold59694 | CTTGGTTCTGGTTCATCGGT | TGACAAGTCAAAGAAGGCGA |
| 733 | scaffold20926 | GAAACAAAAGGGAGTTCGAGA | TTGCGTCATAAGTCTCTGGC |
| 734 | scaffold24504 | GACTGCAAGTGGTGGTTTGA | ATATCCCACCCTCTTCCCAC |
| 735 | scaffold4542 | GAGGAGCATAGAGACCACGC | AGATGGGAAGCAGAGTCGAA |
| 736 | scaffold41947 | GAGGGTAGCAAGTCTCGTCG | GGGGGATAACAAACTGAGCA |
| 737 | scaffold26252 | GATGGAATGGAATGGAATGG | TGTTTTGTCGTTCTCCTCCC |
| 738 | scaffold33526 | GCAAAGTCTCGCAGTCTCCT | TTTTCATCCCTCGTGTCCTC |
| 739 | scaffold20027 | GCAAGATAAAACAAGCATTGTGA | GACAGCTTCTGCAGTACCCC |
| 740 | scaffold2426 | GCACAGCGTAAGATTGGTGA | ATGAGGGTGGTGGAGTTGTC |
| 741 | scaffold11699 | GCGAGAAGATCAACAAGGGA | CCCGTCCCATCATTTCTGTA |
| 742 | scaffold6115 | GCGTCGTTTCGTTCTTCATT | TAGAGACGCAGCTTCCCATT |
| 743 | scaffold25663 | GCTCCCCTTTATGTGGTTGA | AGAAACCTGCTTGTTCCCTG |
| 744 | scaffold23063 | GCTTCAACTGGTTCAGGCTC | CAGCAACATACCAGCGAAAA |
| 745 | scaffold27514 | GCTTTGCTTATGGCAGGTTT | GCAATTTGCAACTCGAGAGA |
| 746 | scaffold5967 | GGAACAAATGGATCGTTGCT | TTTTGGGTCATACAAGTGGG |
| 747 | scaffold33129 | GGAACCATGTCGTCGTCTCT | CAACGACAACGAAAACGAGA |
| 748 | scaffold28053 | GGACAAAAGTGTGTCATGCG | AAGGTAAGCCGTTCGGTTCT |
| 749 | scaffold36127 | GGAGAATAGCAGGCAGCAAC | TTGACCCAAGCATGCAAATA |
| 750 | scaffold2453 | GGAGTTTCGGAAAGAGGAGG | CACCCAATGGATTTGCAGTA |
| 751 | scaffold35208 | GGCATGTAACGTGTTTGCAC | TTCCAATTCAACGAAGCAGTT |
| 752 | scaffold12730 | GGGAACTTAATTATGGGGGC | TGGGTACCGTTCAACCATTT |
| 753 | scaffold20276 | GGGCTTTGTGTGATTGTGTG | CGCCGTTTTCTTGTTGAACT |
| 754 | scaffold4206 | GGGGCCCAACTTTTACCTTA | TATTTTGTTTGCAGCCGTTG |
| 755 | scaffold14184 | GGTCATCCACATTGGTTAGAAA | GAGGAGTGTCCAGGACCGTA |
| 756 | scaffold59607 | GGTTTGCAGATCCGATTCAT | CATGTCGAGAAACGAAAGCA |
| 757 | scaffold43497 | GTCTCCTGCAGCTGACAATG | CGATGGACAGACCCCTAAAA |
| 758 | scaffold56856 | TAAACACAACCCCCAGAAGC | AGCCTGCGGAGATTAGTCAA |
| 759 | scaffold7954 | TAAATTGCGCTGCCCTTAAA | TCATTGACCCTTTTCCGAAC |
| 760 | scaffold2881 | TACCTCCCGACCGAGTAGAA | GCAAATCAACTGCTCCAACA |
| 761 | scaffold21567 | TACTTTGGCTATGGCGGTGT | TACAGAAATGGGTCCTTGGG |
| 762 | scaffold46806 | TAGCCCTACTGGCCCCTATT | CGCACTCCGGTTTCTCTAAA |
| 763 | scaffold197 | TATGCGGGACGGAATTTTTA | TGGGATTCTTTTCATTCTCCC |
| 764 | scaffold35115 | TCAAATCTTTCGCCTCTGCT | TCTCATCACACCAATCGGAA |
| 765 | scaffold6141 | TCAACGAGCTGCATGATTTC | AACCTGATTCACTGTATTCCTGG |
| 766 | scaffold2588 | TCAGTTCGGAGTCTGTCCCT | CTTCATGGATGCAGGTGTTG |
| 767 | scaffold908 | TCCAAATCACAAAGCTTCACC | CCAGAGATGCAAAACAAAGGA |
| 768 | scaffold62456 | TCCGAAAACCCAAGTTCATC | GGAATCCTTTGTCTCTTGCG |
| 769 | scaffold25426 | TCCGATCTTCCGACCACTAC | ATCACATGACAGCGTAACGG |
| 770 | scaffold44145 | TCCGGTAATTCCGAAACGTA | CAACCGTCGGATCAATCTTT |
| 771 | scaffold32533 | TCCTCATAACGTCGGCTCAT | AAGCAAAAAGACGACGAGGA |
| 772 | scaffold13627 | TCGACGAGTCCTCGTTCTTT | TACCCAGAACGAAAACCAGC |
| 773 | scaffold3392 | TCGTAACCGTTTCCTCTGCT | TTTCCGTTGGTCTGATCTCC |
| 774 | scaffold52185 | TCTCGGTTTTGCGTCTTTCT | TTTCAATACAGCCCCCAAAG |
| 775 | scaffold20031 | TCTCTCTGTTCCGGTGCTTT | CCGGAGACTCTGATGCATTT |
| 776 | scaffold37191 | TCTGGAGGATCGATGAGCTT | AAACGCCTCTTGTGAACCAC |
| 777 | scaffold17930 | TCTTGGGTTCAGAAGGGAAA | ATAACACACGGACACCACCA |
| 778 | scaffold6524 | TCTTTCGGAGGGAGAACCTT | AAGAGGGTTCAGGGATTTCTTC |
| 779 | scaffold36398 | TGAAAAGGAATCCAAAGTGG | TAAACGCCAGGTTCTGCTTT |
| 780 | scaffold7600 | TGAAGAGTGTATGGAGAACAACG | AAACCCAAATCAATTTCAATTTTC |
| 781 | scaffold33977 | TGACATCCTCAGGAAATCCAC | AATTCCAAAATGTCCCCACA |
| 782 | scaffold37228 | TGACGAGCTTGCACTTTGAG | GGCTGGCGTATGGATTTTTA |
| 783 | scaffold2103 | TGACTGTGCTCCTCCTTCCT | CCTTGACTTGAATCATCCATTG |
| 784 | scaffold10462 | TGCCATCAAAGGAGTGTTCA | TCATCTGCTCAACATGAGGC |
| 785 | scaffold50371 | TGGAAAACAAGAAGATGAGACC | TCCACACCGTTAAAACGACA |
| 786 | scaffold31711 | TGGACATGGGGTCCAATAAT | CGAGAGGCGAGATAAACGAC |
| 787 | scaffold18935 | TGTGTGGAACAGTGTTTGGAA | TCTCTGGCTCTCTTTCGCTC |
| 788 | scaffold15531 | TTAAGGTTAATGCCCGGATG | TGCTGCTTCTTCTCTGTGGA |
| 789 | scaffold17359 | TTAGAGGCATAGGGGACGTG | ACGTTTGGAACACGTGTGAA |
| 790 | scaffold3076 | TTCAGAATTGGAAAAGATGAACAA | AGGAAGAAAACCTCGTTGGC |
| 791 | scaffold2601 | TTCCCAGTCCAAGAGCACAT | TGTTTTGTTTGGGAGTGACG |
| 792 | scaffold85 | TTCCTCCATGATCAACGACA | GAGGCGACTTCTATTGCGAG |
| 793 | scaffold61147 | TTGCCAGACTGTCTCACCAG | GTGCCCCATCAAACACTTCT |
| 794 | scaffold32448 | TTGCCGGCTATGATCTCTCT | AAAAGGGCTGTTCATGATGG |
| 795 | C18567409 | TTGGATCTTGGCAATCACAA | TGTGTTGACAAAGGCGCTAC |
| 796 | scaffold5266 | TTGGGGTAACCGGTATGAAA | AAGCTTCTCAGGCTGATTCG |
| 797 | scaffold23505 | TTGTGATATTTATTCGCCGATG | GCTGAATGCGATTGTTTTGA |
| 798 | scaffold50194 | TTGTTCCCTCTCTCCCTCCT | CCTGTAATCTCCCCCTCACA |
| 799 | scaffold38149 | TTGTTGCTCTTTTGAATGCG | AAGATAGGCGGCCAGTTTTT |
| 800 | scaffold54558 | TTTAGAGGCAATTCCGTTGG | TCTTCTCCGATTGGTTTTGC |
| 801 | scaffold53997 | CCACGTCGTCGTCTCTCTCT | CCAAATCAAATTGGGGCTTA |
| 802 | scaffold14467 | GAAGGAGAATCTGGGAAGGG | CCACTAAAGGCCCGTGTAAA |
| 803 | scaffold42975 | AACATGGCAGTGATGAGCAG | GGAGAGTTTCATCAACCCCA |
| 804 | scaffold50608 | AACTTTCTCTCCTCCACGCA | TTTCCGCCGATAAAAAGAGA |
| 805 | scaffold35966 | AATCTGGAGTTCCAGCGAGA | GGGATACAAGACCAGACCCA |
| 806 | scaffold12457 | ACATCTCTCCTCCCCCTCAT | TGGTTCTCGTGGACGTATCA |
| 807 | scaffold15051 | ACCTCGTCACCACTGTTCCT | GCTCATCTCCGACTCACCTC |
| 808 | scaffold11680 | ACTTGGGATACAATGCTCGC | CTGCAGAAGAAGGGGAACTG |
| 809 | scaffold2773 | AGCAGAAACGAAACCTGCAT | TCGTTAAGGTGTGGAGTGATTG |
| 810 | scaffold46495 | AGGATCCAGCAGTCCTCTCA | GACGAGTTACTTTCCGCTGC |
| 811 | scaffold25540 | AGTGTCCCTCAACTGCATCC | AAAGCTGAGTCACAGCCCAT |
| 812 | scaffold10946 | ATTGGGTCTGTGAACGAAGC | TTGGATAGTGGATGGTGTGG |
| 813 | scaffold30300 | ATTTAATGAGCGTGCGTGTG | TTATTCTTCCGGCGAAACAC |
| 814 | scaffold21452 | CAACAGTTGCCTCTCTTGGA | AGTGGGAGAGAATGAGGGGT |
| 815 | scaffold776 | CACACCTCACCCTCTCCATT | GGAACGTCTCTCTCTGTGGC |
| 816 | scaffold6508 | CAGACTCGTCGTCTCTGTCG | GTGGAGTCATCGAGGAGAGC |
| 817 | scaffold10471 | CCCGTTGAGTTTTGAGGTGT | ATCACCACCTTTGAGAACCG |
| 818 | scaffold22007 | CCGACTAAAGTCCCCTCCTC | TCAGAGTAAAAGCGCTCCGT |
| 819 | scaffold1323 | CCTCAGACAATGAAGCGACA | TACTGATCGGAACCTCGACC |
| 820 | scaffold2665 | CCTTACGCAAGACCAGAAGC | GACTCGAAGCTGTGGTGACA |
| 821 | scaffold7521 | CGAAGATCCGATTAAAGCCA | TGGTGCATCATTCCACTCAT |
| 822 | scaffold20031 | CGGTGTTGTGAGTCCTCCTC | TCGCAAAACAATCAACAATCA |
| 823 | scaffold2485 | CTCGTCTTTTTGGTCGTTCC | GATGGGGAGTGAGGAGAGGT |
| 824 | scaffold12922 | CTTGGTCCGTTGCTCTTTTC | AACTGATCGAAAGCTCGCAT |
| 825 | scaffold27953 | GAGAGGAGGGGAAAAAGTGG | GGAACTTGTTCCCAAAACGA |
| 826 | scaffold36396 | GATGAAGCAGCTAGGTTGGC | GAGAAAAGAAGGAGGTGGCA |
| 827 | scaffold6476 | GCCTTGGATGAAGACAGAGG | GCGGTGAAGGGTGTAACTGT |
| 828 | scaffold12540 | GCTGCTCATCTTCGTTTTCC | ACAGTAGGCGAGAGAGAGCG |
| 829 | scaffold11681 | GCTGTGTTCCGTGCCTAAAT | CTAGCCAAGTGAAAGCCCAG |
| 830 | scaffold33834 | GGATGGAAAATGCAAGCTGT | GAGAGACACGACGACGATGA |
| 831 | scaffold18548 | GGCAAAAGAAGAGACAGCGA | AGTGGGGGACCATAAACCTC |
| 832 | scaffold25705 | GGTTATGCACACTTTATCATCAGG | GGGTTGTTCTGGGTGAGAGA |
| 833 | scaffold30705 | TAGGGAGGAGAGGGACACCT | TTAGCAGCGCACTTCTTCAA |
| 834 | scaffold6720 | TCTCTTGCTATTGTGTAGATTTGTTG | GATGACCGGAATTGAGAGGA |
| 835 | scaffold12608 | TGAAGAGAGAGCCAAAGACGA | TAACCAACCCGACCCAGATA |
| 836 | scaffold575 | TGACGGTTCATCCAGTGAAA | CGCAGCAAAAACAACAAATG |
| 837 | scaffold23044 | TGAGTGTTGGCGTTACCAGA | AATTTAACTTCAAGGGCCTCA |
| 838 | scaffold21439 | TGCAGGGGTTTTGGTATTGT | TGGTTTGTTGTTGTGGTGCT |
| 839 | scaffold1044 | TGGATTCACCCATTTTTGGT | ACGACCAATCAAAACGAAGC |
| 840 | scaffold12251 | TGGATTGTCAACGCTCTCTG | GGGGTTTTGGTAAGTGGGAT |
| 841 | scaffold5246 | TGTCATACCATTCTTACGATTTTGA | GATGCCACACTGTCAACCAC |
| 842 | scaffold10993 | TGTCTTTGAGCCGAAGGATT | GTTCATGCCCTGGAGACAAT |
| 843 | scaffold689 | TGTTTTCCTTCTTTGGCTTGA | GGCTTTGAAATCACCCTTCA |
| 844 | scaffold2873 | TTCGAGCCCACCATATTTTC | GGAAGATGAAGCGGACGTTA |
| 845 | scaffold32967 | TTTGCCAATAAGGACCCAAG | TCGTTTTAACCGGAGGAGAA |
| 846 | scaffold24504 | GCGTCTTACTCTGTTTCGCC | GTAGGTTTCTTCGGTGTGGC |
| 847 | scaffold11310 | ATTAAGCCCGTGAAACAACG | TCGGGAGAGAGGATGAGAGA |
| 848 | scaffold20620 | CACATCTTATTGACACGCGG | GAGGAGAGCGAGAGAGACGA |
| 849 | scaffold27457 | CGAATAGAGTCGTTGGAGGC | AACTCCATGAAAATGGTCCAA |
| 850 | scaffold14871 | CGCTTATGTCGCTTACCTTTG | CGACTATTATAATGGGCCGGT |
| 851 | scaffold10208 | CGTCGACCCATTAACTTCGT | TATAAGACGATTCGCCGCTC |
| 852 | scaffold14808 | GGGAAGGAGTTGCGTAATGA | GACGAGTTCACACACACGCT |
| 853 | scaffold29133 | TGGTGGGTCTGGTTTCTTTC | AATGAAAAAGCGAGTTGGGA |
| 854 | scaffold6308 | CATCTGAGGCGTCTGTGCTA | AGAGAGAGAGAGCGTGGTCG |
| 855 | scaffold3173 | AAACTGAACCCGTGGAACTG | CACTTCCAAAAACGTGGGTT |
| 856 | scaffold23048 | AACCGTTACAGACACCGCTC | AGGAAGCTTTATCCCCCAGA |
| 857 | scaffold7884 | AAGCTCCATTTCAGTCGGTG | ACCGCGGTTAACCCTAAAGT |
| 858 | scaffold11988 | ACCTCCAAAAACCCTCGTCT | GGGAGAAAACCCACATTTGA |
| 859 | scaffold21570 | AGGAGATGGGAGGATCTGGT | AAGCGAGTGCTCTTCTCAGC |
| 860 | scaffold1000 | ATTGTAAGCGGCAAAACCTG | GACAAAGCGAATACGGTGGT |
| 861 | scaffold25463 | CAACAGCGAGCATACCAAGA | TTTGTCACTCGCCATCTCTG |
| 862 | scaffold9444 | CCGATTGACTGTTCATGGAAT | TTCTGAAACCTGATTTAACCGAA |
| 863 | scaffold13348 | CGTCGCACTCTTCACCACTA | GTCTTCAGCCATTGTCCCAT |
| 864 | scaffold43653 | CTCATGGAACTCGACACCCT | GCTCCAGTGCCTGAGATTTT |
| 865 | scaffold23795 | CTGAAAGCTTGATTGGAGGC | GGTGATTATGTGGATTAATCGGT |
| 866 | scaffold1554 | GAGCTTCCCTACCACCAACA | CTCACGATCCACCTTCCTGT |
| 867 | scaffold14434 | TCCACCACAGGTCGTTGTTA | ACAACACAAACGCAAAACCA |
| 868 | scaffold50787 | TGCAGGCTCTCTCATCTCAA | CAAGCAACAAACGCTAGCAA |
| 869 | scaffold39118 | TGCCAATGGGATAAAACACA | AGCACCAGAGCGATCATCTT |
| 870 | scaffold17160 | TGCTCGTCGCTAAACCTTCT | CAAATGACCCCTAAATCGAAA |
| 871 | scaffold2154 | TTACTACGACAGTGCCACGC | TTGCAAATGTGTACAAACCACA |
| 872 | scaffold3952 | TTCTCGGTGGAGTATCGGTC | ACACACAGTCGCTCTTGTGG |
| 873 | scaffold33019 | TCTCACATGTACTGACCCAAAA | GCGACTGCGGATATGAATCT |
| 874 | scaffold44347 | AGAGCCTATCGACCTCCCAT | CAGCTAACGGAACAACCGAT |
| 875 | scaffold651 | CCACACGTCAAGCGTAGAGA | GAACCAGCGACAGAGAGGTC |
| 876 | scaffold2324 | CTCCGTCGAAGTTTGAATCC | AGGAGACGCGAAATGAAAGA |
| 877 | scaffold1481 | GACCAACCTTTGAAGAACCC | TTGGAGGCACACTTCTTTGA |
| 878 | scaffold36129 | GGCTCATCTTGGGAATATCG | GAACATGAGCTTGTGGTTGC |
| 879 | scaffold38528 | TGAATGAAGAGCATAACTGCAT | AAAGCCCTTTATTCACCATCTG |
| 880 | scaffold10593 | TGCCAATTATCCACACTTGC | TAGAATAATGGGGTCGCTGG |
| 881 | scaffold5891 | TGGGAATATCGATCACTGAACA | ATGATCGCACATTGTTGGAA |
| 882 | scaffold26376 | GCTGTATCATCTTGGTCAGCC | CATGAACCGAGAAAGAGGGA |
| 883 | scaffold5123 | AAAAAGTAGAGTCGTATCTGATTTCCA | TTCTTGCAATAATATTTGGTTGAGA |
| 884 | scaffold29081 | AAAACTGAATACGGAAAATATGAACA | TCAGTCCACTTTCGCTTTGA |
| 885 | scaffold23242 | AAAGCCAACACAGAGGGAAA | TCTGAGTATTCTCCCGCACC |
| 886 | scaffold58844 | AACACACAACCATTTGGGGT | CAGCGGGTCCACATCTTTAT |
| 887 | scaffold2058 | AACACTTTGGCGTGACATGA | GTCCAAACACATGGACCAAA |
| 888 | scaffold38115 | AACATACTTCCCCTCGGGAT | GGGCGAAGTGGTTAAAGTGA |
| 889 | scaffold6952 | AACCAACATTAAGTTTGTGTCAATTA | TCGAGTCATTACAGGTCTGGAG |
| 890 | scaffold42634 | AACGCAGGAGGCTTCCTATT | TCAATCACTCTTGGTGCTGC |
| 891 | scaffold15531 | AACGCTGGAAAACCACTTTG | TCTCTACGGCAGGTACGCTT |
| 892 | scaffold7351 | AACTTATGCGGAGAGCTTGC | GGAAGTTTCTGCGAGTTCCA |
| 893 | scaffold16432 | AAGAATCCTTTGAGCCCGTT | TACCAAAGACCCGTTCAAGC |
| 894 | scaffold35709 | AAGACGCCGAACAAGTGAGT | ATGGTCGCCGTTGAATAGAC |
| 895 | scaffold25568 | AAGATATTTCTCCTCTTTTAAGCCC | AAGATACGTCAAACAAACACAAAA |
| 896 | scaffold10976 | AAGCTCGATGAACGAACAAAA | AACGCTTGATTTTTCGAGGA |
| 897 | scaffold18550 | AAGGCAGAGTCCTCGTCTGA | CTGGCAGCCTTGGTAGTAGC |
| 898 | scaffold24272 | AAGTGCCACTGTTTAGATGCAA | TCAACTTCTTGGACAGGCAA |
| 899 | scaffold6142 | AATCAGAAGCGGATACGTGG | TTTTGATTTGTGTGTGGTTTACA |
| 900 | scaffold30880 | AATCTGCGTTGTTCAGTTGC | GCGGACGGATATCGTTGTAT |
| 901 | scaffold4304 | AATCTTTGGCCCTTCCCTAA | TGCAAGACTTGGCTCAACAC |
| 902 | scaffold42423 | AATGACGATCAACATTGCGA | ATACAATGCTGGAGAAGGCG |
| 903 | scaffold29091 | AATTAAATAAGCCAACTGCACAA | CGAGTAAGTGGTCGAATGTGAA |
| 904 | scaffold18930 | AATTAAGATAATAAATACATGGCGTCT | TCTTTGTCCAGCAAAAAGGAA |
| 905 | scaffold18961 | ACACCAAATTTGTTAGGCGG | TGGCGCAACTCAATGAAATA |
| 906 | C18548040 | ACCATACCGAAATTCCCACA | CACCACCTCCACCTACACCT |
| 907 | scaffold22862 | ACCCACACAACAAAAGCCTC | TTTGCAAAAGCCCATGTGA |
| 908 | scaffold6174 | ACCGGAAAATCGAAAATGGT | TCAGACACTGTTTTGGCTCG |
| 909 | scaffold37702 | ACCGTCCCATGGACTGTAAC | GGGATCTCTGCTCTACACGG |
| 910 | C18543684 | ACGCCTTCTTCTTCCGGTAT | TAACCCGATGATCGCAGACT |
| 911 | scaffold24724 | ACGCGCAAAGAAATTTGAGT | TTGCCATATTGAACAGCCCT |
| 912 | scaffold6555 | ACGGAGTAGAGGCAGCAAGA | AGGAGCTGCCATGTTGTTCT |
| 913 | scaffold8220 | ACGGTTGCTGGGACATTTAG | GTGAGGGTCCAAGATGCAGT |
| 914 | scaffold15758 | AGACATCGTTAAGCCGTTGG | GGAAAGTCACACAGCGACAA |
| 915 | scaffold9141 | AGATCGTGGGTCTGGTGTTC | CGTCGTTGATCGTGTCTCAA |
| 916 | scaffold64217 | AGCCGGTTGAACGAACTATG | GTTCGCACATGAGGGACTTT |
| 917 | scaffold33031 | AGCTGGAAAAGAAACCCCAT | CCACACTTCAAATGCGTGC |
| 918 | scaffold2743 | AGGCCACTGAAAAGAAGGTG | CGATTCGGATCAGCCTAAAA |
| 919 | scaffold25473 | AGGCTGTCAAGAAAAGCAGG | GCCTCCCCATCTCTCTCTCT |
| 920 | scaffold15452 | AGGGTTGCGAACAGTGTACC | GCCTGATGAGCTTCTTTTCG |
| 921 | scaffold30790 | AGTCACTGAGGCCAAGAGGA | CGTAACCGATCATGACACCA |
| 922 | scaffold11323 | AGTTAGATCATTTTCACCATCAAAT | CTCTTTGTCTGCCACGTTTG |
| 923 | scaffold21246 | ATAACTAAGCCCCCGGAGAA | CAAATTACACATGTTGGCCAGT |
| 924 | scaffold2003 | ATACCAGCCGGGTGTAAATG | CCTATCCCTTGGCAGTTCAA |
| 925 | scaffold4961 | ATATTCCGGTCTGGTTTCCC | TCGCTTGAAGTTGATAGGTTTG |
| 926 | scaffold16592 | ATCCTCGGACAATCAAATCG | CCCAACCAATTATAAAAACATGC |
| 927 | scaffold38044 | ATCGCTAATGGTGTTGGGAC | CCTTGCTCCACTTCTTGAGG |
| 928 | scaffold64339 | ATGAACGTGCTTCCCATTTT | GGCTAAGGTTTTCTCCAGGG |
| 929 | scaffold33274 | ATGACACCAGCAGCAACAAA | CGCTTTTTGTGCCTCTTTTT |
| 930 | scaffold53032 | ATGCATGGTTGCTACGGACT | TGGCTTATTGCTGCACAGAG |
| 931 | scaffold2353 | ATGCCTGGGAGATTAACGTG | GGACAGCCTATCTGAGGCTTA |
| 932 | scaffold16119 | ATGCTTGTAGCAGCCAAGGT | CAAAGAAAAGCCCAACCTCA |
| 933 | scaffold40264 | ATTGCTTCCCTCAACACGAT | GAAAATGGACCGGGGATTAT |
| 934 | scaffold15043 | ATTGGCGAACCTACATTGCT | GTGGGTTTGTTGAGGAGGAA |
| 935 | scaffold2940 | CAAAAGAGTTATTATTGAATGTGGAGA | TGATGTCTCAAAAGCATCCA |
| 936 | scaffold904 | CAAAAGCACAAATGTTTGGTACA | AACAAATTCCCGAATCCACA |
| 937 | scaffold25465 | CAAATTTGGCAGTTTGCATAG | TTTTGCTTCTAATTGGGACGA |
| 938 | scaffold23759 | CAACACACACACACGCACAT | CCAGCAAAGCTCTATGCCTC |
| 939 | scaffold31117 | CAAGGTAACGGGAATGAGGA | AGTCACAAAGGGGCAAACAC |
| 940 | scaffold16606 | CACAGCTGTCTTGGTGTCGT | GAAGACGAGGCGATGAAGAC |
| 941 | scaffold8329 | CACAGGACTTGCAAAGTTGG | TCTTCTTCAGCCCAGAGAGC |
| 942 | scaffold4081 | CACGCCCTCATCCCTACTAA | CTGTTTGCGAGTCTTGGTTG |
| 943 | scaffold12896 | CACGTTCACACGCCTACACT | ATTTGCCAAGTCCATCTCGT |
| 944 | scaffold17453 | CACTCAAAACCAATCCCAAA | CAGACATCGAAAAAGACGAAGA |
| 945 | scaffold12740 | CACTCGCTTCTCTTGTTCTCG | CAGCACTGGATTTTGTTGGTT |
| 946 | scaffold21516 | CAGCCATCTCATTCCGTTTT | GTTTCGACCTTGAACTCCGA |
| 947 | scaffold16856 | CAGGCTCGAGACAATCCAAT | ATGTGAGGGGTGGTTGTGTT |
| 948 | scaffold14476 | CATGTACCTCAGCTTAAAGTGTTGA | CCTGGGAGTGATTCAAGGTC |
| 949 | scaffold12012 | CCAAAATCAATGGAGTCCCA | TCAAAGACACAGCCTTGGTG |
| 950 | scaffold50042 | CCAAAGGTGTCACGCATGTA | TAATTCGGCCTCTGTGTTCC |
| 951 | scaffold23162 | CCAACTATATTAGGCTGAATGCAA | AGGAACGTGAGGTTCAAAGC |
| 952 | scaffold9093 | CCAAGCTGACCTCCAACCTA | TCCTGTTCGAATTCGTTTCC |
| 953 | scaffold3965 | CCAAGCTGATTGGTTCAGAGA | AAAGTCGTCATCCAAGTCGC |
| 954 | scaffold20191 | CCATCTTTTACGCCCTCTCA | CCCGAAAGAGACAGTTTCCA |
| 955 | scaffold10831 | CCATGAAGTTTGTAACGCTCA | ACTTATATGCCAAGGCACGC |
| 956 | scaffold15891 | CCCAATGGGGTCTGTTTTAG | GAACCATGTTGGGTCCTCTC |
| 957 | scaffold25037 | CCCTACCGTCTCTTCTTCCC | ACGATTCCAAGGCAGCTCTA |
| 958 | scaffold14217 | CCCTGCTTGAGATAAACCCA | CATGATGATGCCATACGCTC |
| 959 | scaffold40743 | CCGAACCGAATCTGAACCTA | CTGAACCGAACCCAAACCTA |
| 960 | scaffold50086 | CCGAAGTTTCATGTGCGTTA | CGAAGCAGGAGAGGATGAAG |
| 961 | scaffold689 | CCGACTTGACAGGTCCGTAA | TTGTCCATGCTTTAGTTATTTCTGA |
| 962 | scaffold6812 | CCGAGTATGAAGCACGATGA | ATCCTTCTGCCAGTCTGCAC |
| 963 | scaffold5653 | CCTCGATGAGACGCAATTTT | CATTCAAGGTTCAGCTCCTG |
| 964 | scaffold12285 | CCTTTGTCTAAAATAGGAAAAGACCA | AAGCCAAGCCAGAACAATTT |
| 965 | scaffold555 | CGACTGTACGTTCGGTTCAA | CTTTCTTTTGCTGCCGTTGT |
| 966 | scaffold3063 | CGAGATCAATAGGTAGCGGC | ACGTGGTCTCAGATTTTGGG |
| 967 | scaffold42915 | CGAGATTCTTGACTGCCTGA | TCACCATGGGAGTTTTAGGG |
| 968 | scaffold56697 | CGAGTTGGAGCCTACTTTGG | ACAAGCAGTGCTGCAAACAA |
| 969 | scaffold17387 | CGCAAAAATACAATCCAACC | TTACACCGTTGCTTTGGACA |
| 970 | scaffold31182 | CGCAACTCATACGATTCGGT | CCTAAGAGCATGATTATTGGGG |
| 971 | scaffold10015 | CGCATCTTTGAACAAGCAAA | TATGCACAATCATTGACGCC |
| 972 | scaffold26630 | CGGAACATCACCCAGTCTTT | TTATCTTCCCAAGCCACCTG |
| 973 | scaffold49411 | CGGACTTCACTTTCTCCCAA | TGGACTGAGCTTTTCCATCA |
| 974 | scaffold8419 | CGGAGCAAAACACGAAGAAC | TCCCGATTCATTACAACATCA |
| 975 | scaffold6820 | CGGGACATGGGTGATATGAT | TTGGTTTAAGCCCAGAAACG |
| 976 | scaffold4777 | CGTGTGGATCCTCATCATCA | CTTTCCACCAGCTTCCAGAG |
| 977 | scaffold14234 | CGTGTTCAACGGAGTCAGAA | ACAACTAAGGTACGGGCGG |
| 978 | C18344031 | CGTTTGCTTCTTGCTCGAAT | TTTTACGCTAAATCGCAAAATTA |
| 979 | scaffold19001 | CGTTTTCGAACATGGGTTCT | TGGTATCCGTCCGTCTTTTT |
| 980 | scaffold18190 | CTCCATTTTGATTTCCTGGC | TTAAGGTCAGTAACGGCCCA |
| 981 | scaffold26820 | CTCCTTCTCCGTGACACCAT | TCGTTTTGCTGACTTCTGGA |
| 982 | scaffold1577 | CTCTGTCTTCTTGCTGCCCT | GGATGGTGCTTTGTCTCGAT |
| 983 | scaffold15223 | CTGATTTCATTTCTGCTGCG | TGGTTTTTGATTTGGTAAAGCAT |
| 984 | scaffold4923 | CTGCAACCGATTTGGAGTTT | CGATCTGATTGATGTGTGGC |
| 985 | scaffold2674 | CTGCCACATGCTGACCTAGA | TGGCGAAAGGTAGCAAAAGT |
| 986 | scaffold11771 | CTGTGACGTTGTGATTTGGG | TGTTGAATTTTTCTTTAATGGGTT |
| 987 | scaffold728 | CTTCATCGACCCACCTTGTT | CAAGTTCAAAGGCAAGACCG |
| 988 | scaffold13204 | CTTCCATTTTGCAAAGAGTGTT | GGTTCTAAACGGTGAAAGCAG |
| 989 | scaffold2822 | CTTGTGACATGCGAAGAATCA | GGAGTGGGGAAATTTTTGGT |
| 990 | scaffold22894 | CTTTTGACACCAACCAAGCA | TCATTTGTCTCATTTATTCATTCCA |
| 991 | scaffold27468 | CTTTTGCTGCAGCTGTTTTG | CAAGAAAACGGGAAACAATGA |
| 992 | scaffold10312 | GAAAGCTTCATCCCGAACTG | AATGAAAGATCCAGCCATGC |
| 993 | scaffold14115 | GAACAACTCCTGACGCACAA | GAAAGATGTCATTGCGCCTT |
| 994 | scaffold138 | GAAGCCTTCAGAAATTGCCA | ATGCGCCATACCGATCTTAC |
| 995 | scaffold21433 | GAATCCCCTATTTTTGGGGT | CGACAATGCTATTCTTTCTCCA |
| 996 | scaffold38917 | GAATGCTAATTGACGCACGA | GCTCTGATCATACGGACGCT |
| 997 | scaffold34169 | GACATGTGGCAGCTAACTTTG | ATCACCAAGACCCCTACATTT |
| 998 | scaffold4355 | GACGTCAAAGCTCATCGTCA | TGCATCATCTCCTGATCCTG |
| 999 | scaffold602 | GAGCAATGAGCGAGGCTAGT | AGCTGAAACAGATCAGAAGCG |
| 1000 | scaffold4104 | GAGGGGATTCATACAACGTCA | CATTGCCACAAGTGAAAATCTC |
| 1001 | scaffold9329 | GAGGTCGATTAGACCCACCA | GCCGTGTGTGTGAAGTGAAT |
| 1002 | scaffold9144 | GATCACATGCGTAAAGGAGGA | TCATGTCCTCGTAGCTGGTG |
| 1003 | scaffold44912 | GATCGGTTTCAACGAAGGAA | CAACACTTGGACGCAACACT |
| 1004 | scaffold60962 | GCACAGACGCAACAAGTGAT | GTCAAGTCAAGCCGAGCAAT |
| 1005 | scaffold7147 | GCATATGGGCAGAATATCCAA | TTCTATGTCAAGACCCTATGGAATC |
| 1006 | scaffold37294 | GCATGTTGGCAAAACAGAGA | TCACGGGGAAATTCTCAAAG |
| 1007 | scaffold28180 | GCCCTATTCCATTATGCAGC | CAAGCAATTGTGGAAGCTGA |
| 1008 | scaffold30849 | GCCTCCGATCAACAAGAAAA | ACCAGCATGGTCAAGTCGTT |
| 1009 | scaffold5322 | GCCTTTCTCATATCTTGCATGT | CAGAGTCAGTCTACGATCGGG |
| 1010 | scaffold29627 | GCGACTGGACCTTTCTGAAC | AATGACCCTTCGCAACAAAG |
| 1011 | scaffold22556 | GCGCCAGTTGTGAACAGATA | TAACTTTCGCTCTTCCACGC |
| 1012 | scaffold6283 | GCTCCACGCTTGTGTAGGTT | TGACAAAGGCAGAGAGACCC |
| 1013 | scaffold36334 | GCTCGTAAGACAAACGGCTC | AACTCTCCTCCTCGCTCACA |
| 1014 | scaffold17763 | GCTGAAAGCTGAATAATCCCA | CCGAATGAAAGCGTTAAACC |
| 1015 | scaffold19944 | GCTGCTTCAAGGAGGTTCAG | CCATTGGAACCTGGGAATTA |
| 1016 | scaffold2604 | GCTGGCAAATTCAATCTCGT | AATAATATCCCCTTTGGCCG |
| 1017 | scaffold14871 | GCTTAGGATTGGTTTGATTGAA | CCATTAACACCCGCTTCCTA |
| 1018 | scaffold64346 | GGAGAACCAAACATGGCCTA | CGCAAGATCACAAACGCTTA |
| 1019 | scaffold8021 | GGAGGAACGTTTCCAAATGA | TCCCCAGAAGTGGTCAAATC |
| 1020 | scaffold13221 | GGAGGGAGAGAGAGAGAGGG | TAAACCGGTATTGGGTCGGT |
| 1021 | scaffold49261 | GGATGATTCTTGTTTTGTGGG | AGCCGGAGCCTATAAGAAGC |
| 1022 | scaffold1669 | GGATTGGGTACAAGCCAAAA | GCATGGTGAGTCTATCCATACAA |
| 1023 | scaffold38044 | GGGAGAAGCTGCATCAAAGA | GGGGTGGAATAGGTCTGGTT |
| 1024 | scaffold64697 | GGGTCCCATGTTGGAGTATG | GGGAAGATAATCGTCGCAGA |
| 1025 | scaffold19626 | GGGTTGGTAGGGTTTGAGGT | GGTCGAATAGGCAACGAAAA |
| 1026 | scaffold8504 | GTAAACATGAGGAGGCGGAG | CCATTTCACCTTCTGCTGGT |
| 1027 | scaffold9805 | GTAAAGAGTATGCGGCGGAG | TGAAGACTCACCTTTTCATTACAGA |
| 1028 | scaffold7991 | GTCATTGCTATGGGAGGCAT | AATTGTAGGCTCCCGGAAAT |
| 1029 | scaffold77 | GTCCTTCTCTGCTTTGTCCG | TAATCAACGGGGTTAGTCGC |
| 1030 | scaffold17002 | GTGTAAGCGACGGGGATACT | GCTAAATTTCATCTCGTTCTCATC |
| 1031 | scaffold18519 | GTGTCTTCGTGCAGCTGTGT | TTGTTGAAGCATGAACCTTGA |
| 1032 | scaffold49424 | GTTTTAGCGTCCCAAACCCT | TGCGCAATTCCAAACATCTA |
| 1033 | scaffold21686 | TAACCGAACCGGTTTTTGAG | GAGAAGCTGAGATTGGTGTGC |
| 1034 | scaffold830 | TATGGACCACATGCCCCTAT | ACTAGGGGCGGATTCAAAAA |
| 1035 | scaffold31066 | TATTGGCAAAACTGAGGGCT | GATACGACCCGTGTTTGGAT |
| 1036 | scaffold61939 | TCAAACACTTGTCCGTGCTC | AACGTGGCCTTGAAGCTAAA |
| 1037 | scaffold435 | TCAACACTCTTCTCCGAGTTACA | TTGCATGACTTTTTGTATTTTCTAA |
| 1038 | scaffold2518 | TCACCACCGTATGTCACTCC | CCTCCTCGCTTGCCTATTTA |
| 1039 | scaffold3759 | TCACTTGGAAGAAGCCACAA | AGTTCATTTCAGCCGAGCAT |
| 1040 | scaffold23242 | TCAGCTGCCTCCTTTTGTTT | AGCCTCCAGAAGCATCACTT |
| 1041 | scaffold25972 | TCAGTTGCGTGTGCATGTTA | TGGTTATTGCATTATTCTGTTTTG |
| 1042 | scaffold10009 | TCCAGCTCAGCACATGTAAGA | CCAAAATGTGAAGACATGACC |
| 1043 | scaffold64234 | TCCATGACTCTCGTGCTGTC | TGTGTGACGAATTCACTGGG |
| 1044 | scaffold61499 | TCCATTTCAAGAAGTCGGATCT | CAACACTTGCACCCATTTTG |
| 1045 | scaffold13220 | TCCCTTTTGCTCATAGCCAC | CGCCGTTTTGAAGACAGAAT |
| 1046 | scaffold9448 | TCCTATTTTGTTTCCCTTGCAT | GACTATCCGGGTTCAACGAA |
| 1047 | scaffold58078 | TCGAAAAATTCGAAAATCCAA | GTATGCGGATCGTTTGACCT |
| 1048 | scaffold41195 | TCGAGCTTGATTTATGGCCT | CTGATGATGAGCACCGAAAA |
| 1049 | scaffold11972 | TCGGATCTTGTCTCCAGATTTT | ATGGTGCGCTAATGTTGTGA |
| 1050 | scaffold17178 | TCGGATTTGCTTCATCTTCC | ATTTGCAGAGAGAGCCCTTG |
| 1051 | scaffold4637 | TCGTAACGAAAAAGCAAAATAACA | AAGTTTCTTATTCGCCCGGT |
| 1052 | scaffold17629 | TCTCATCGCTCTCAGGTGTG | TCGGGAAAAGACAGAACAGC |
| 1053 | scaffold14659 | TCTCCACGTTGTCCATCAAA | TTTAATCAACGATGCTTGCG |
| 1054 | scaffold50274 | TCTCCCCTTCATTTGATTCG | TGTGAAAGCCTTGAACACACTT |
| 1055 | scaffold27128 | TCTCCGAGAACATCCCTGTC | CGCAGGGTTGTTGGAACTAT |
| 1056 | scaffold2601 | TCTCGTCTTTGTTAAATTTACTAGGTT | AAGGTGCACGTAAAGCTTGG |
| 1057 | scaffold8371 | TCTTGAATGGGAGATACGCA | CGCATGAAGGAAATAAGTTGC |
| 1058 | scaffold27452 | TGAAACCCGAAGTAACACCC | GATGGGAGATGCAGATGGTT |
| 1059 | scaffold48307 | TGACATCAACGCAGATCCAT | TCTCTCAACAAACACGCAGG |
| 1060 | scaffold36824 | TGACCAATCCCTTATTGTGGA | TGTTTTGATTAGCTTGTCGATTG |
| 1061 | scaffold21341 | TGACCAATTTTTATTTCCCCC | CTTGGTCTAAGGCATCCGAA |
| 1062 | scaffold12047 | TGATGGGCAAGTGTATTCCA | AGCTCGGATAAGGGGATGAT |
| 1063 | scaffold3129 | TGATGGGCTATCTCGGAAAC | AGGAGATTTGTCCTCCCCTG |
| 1064 | scaffold19067 | TGCAACATACTCTCCTTGCG | TCTGGGTACCACAAACGTGA |
| 1065 | scaffold13425 | TGCACAACTTTCCTACCCACT | TTTCGAACCGATTTTCTATGC |
| 1066 | scaffold56354 | TGCACGTTGACAAAAGATGG | GAGCGTCTTGAAGATTTGGC |
| 1067 | scaffold38868 | TGCCGTGATATGAAAAAGGA | ACAACTCGCGTTACTCGACA |
| 1068 | scaffold42555 | TGCGCATCCGTAGAATATGA | TCCATCCGGTTTTCTCTTTG |
| 1069 | scaffold830 | TGCTCTTCTCTGCTTAGCCC | AATCTGGTGCTCACAACCAA |
| 1070 | scaffold2643 | TGCTGTAACTGCGGTTTGAA | AGTCAATGGCCGTAGCTCCT |
| 1071 | scaffold43442 | TGGCATTTGAGTAGTGGCCT | AGTGGCCCACACTTCCATAG |
| 1072 | scaffold7226 | TGGCCGGTTAATCTTTTCTG | TTTTCCAGCGCCATAGATTC |
| 1073 | scaffold20636 | TGGCTCACGAACTTCAAACA | GCTGTTCATACCTCAACTCGG |
| 1074 | scaffold2407 | TGGTAAATGTCTCGGCTTCC | CGCAGGCTTCTGTATGTGAA |
| 1075 | scaffold7407 | TGGTGCCTTAATCTCTCACG | TCCAAACCCGAAATAATACCC |
| 1076 | scaffold33837 | TGTCACAATATTATCGCGTTGAC | AAAAATAACCAAGATACACTGCAAAA |
| 1077 | scaffold15408 | TGTGCTTTGCGTTTATCAAAA | AAATCCGAAATACCCGATCC |
| 1078 | scaffold1672 | TGTTTCCAAACAGGCTCTCC | AGATACGAGGCTTTGGGCTT |
| 1079 | scaffold12187 | TGTTTGAGGGTAGGGGATTG | GGATCTACGCCCACACAAAT |
| 1080 | scaffold9974 | TTAAATTGAGTCACTTGTCCACTAA | TCGCTTCCTAGGTGCAGAAT |
| 1081 | scaffold8828 | TTAGTGGACGTGACATGGGA | GATTTCGGTGACCTCTCCAC |
| 1082 | scaffold1163 | TTATCTTTTGCCGAGGATGC | TCACACAAGAAAGCAGCGAG |
| 1083 | scaffold249 | TTCAGGCTCTGGTAGAAACGA | TTGAACAGATGATCTCATAGGCA |
| 1084 | scaffold20926 | TTCCGATTGAGTATGTGAAATTAAA | GGATGAAAGGGAGTGCAAGA |
| 1085 | scaffold22352 | TTCGGAACGACTCAGACTCA | TCATTTTCTTTGTGGACATTCAA |
| 1086 | scaffold64033 | TTCTCCTTCGACTGTTCTGAAA | GCCGTTTCTTTGGTTAAAGC |
| 1087 | scaffold19034 | TTCTCGTTCCTGTTCCTGCT | ACAAAAGGGCCCATTAGGAC |
| 1088 | scaffold13502 | TTGCAAGTCCTCGTGCTTAG | GCAAGTCCTTGTGTTTGGGT |
| 1089 | scaffold8808 | TTGCCGGTAGAGATACTTTCG | TACATGAGTGCATGCGGATT |
| 1090 | scaffold19385 | TTGCTCATTCAGCACGAATC | GAAAACGTCCCTCCCTCTTT |
| 1091 | scaffold19038 | TTGGAGCGAGTTCTCATCCT | ATCCTCGTGCAATCTCGTCT |
| 1092 | scaffold1597 | TTGGTTGAGACTTTGCTTTTCA | AACAGTCCCATTGATCCTGC |
| 1093 | scaffold4624 | TTGTAAATTAACTGCATTCAACCA | CCGCATGGGAATACTTTTCT |
| 1094 | scaffold32807 | TTGTAGGAGGATTGGCTTGG | AAAGGGGAGAGAGGGTCGTA |
| 1095 | scaffold16377 | TTTACCTTTATGCGTTGCTCA | ACGTGTGTGTTTGCTGCTTC |
| 1096 | scaffold3414 | TTTCTAAACCAATTTAATACACCAAAC | TGATTTTATGGTTTTGGGGG |
| 1097 | scaffold22860 | TTTCTGACCATGATTTGCCA | AGGTGGGAGGAGTTTCGTTT |
| 1098 | scaffold4744 | TTTGGATCATCCTACTTTCAAAC | TGGACGTCAGACGAAAAACA |
| 1099 | scaffold6887 | TTTGGGATGTGTTGTTTGGA | TCACCGTTAATAATGTTTGAAGGA |
| 1100 | scaffold13942 | TTTTCCACAATGCTAGTTTCCA | TGTGTGTGCTTGTTGGTAATGA |
| 1101 | scaffold32032 | TTTTGAGTTCACAAGGGTTGC | ACCTGAGCCTGAAGTCCAAA |
| 1102 | scaffold2475 | TTTTTCATGGATCTGCAACG | GTTTCCACGGTCTTCTGAGC |
| 1103 | scaffold5437 | TTTTTGTCGGGTTCATAGGC | TTTGGATTTACCCCATGGAT |
| 1104 | scaffold10482 | CCCTCTTTGCAGAAATCCAA | TCCATGGTCCTTTCTTCACC |
| 1105 | scaffold21520 | AAAAGACCAGAAGCTGAACCTG | CGAACCGACCCAATGATTTA |
| 1106 | scaffold9985 | AAACAGGGATGCACAAGGTC | TTGCCGCTCATAGTTCTCCT |
| 1107 | scaffold29009 | AAAGCCTTTGCCTGTGAGAA | ATCTGCGAATAGCTTCCAGG |
| 1108 | scaffold36524 | AAAGGATGCTGGTGGTGTTC | CTGCCGATTCATCAGGTTTT |
| 1109 | scaffold44695 | AACATCCGTGGCTGAATCTC | TGATCATATTCATATCTCCCCAAA |
| 1110 | scaffold5712 | AACGCAGAATCAGGCAAAAC | TTAGGAGTGAGCTCGGGAAG |
| 1111 | scaffold8831 | AAGAAGCGCCTCCTGTTGTA | ATCGGCCCTTTGTTTCTCTT |
| 1112 | scaffold21268 | AATGCAGAACCCATGAATGTT | TGACTTTGCAGATTCCCCTT |
| 1113 | scaffold1377 | AATGGAATGTGCTGGCTTTT | CCCATCCATCCATTCACTCT |
| 1114 | scaffold6013 | ACAAGGTTAAAACGTTGCCG | GATTGCTCCAAACGAAGGTC |
| 1115 | scaffold49420 | ACAATGGTGGGAGTGGAGAA | GAACAAGTCGCATTGTTTATTGA |
| 1116 | scaffold3464 | ACAGGTGGACAGAGCCAGTT | CCAAATAAGACTCACAAAAGGACA |
| 1117 | scaffold26032 | ACATCAACCAAATTGTGCGA | CGGGACTTGCATGTTCTGTT |
| 1118 | scaffold39124 | ACCACTTCCATTGATTCCCA | TAAATCGGACATTCGTGCAG |
| 1119 | scaffold1597 | ACGTCTCTTGTGCAGAGCAA | AGCAAGAGAGATCAAGGGCA |
| 1120 | scaffold63992 | ACTATCCATGTGGGCCAAAC | TTCTCTTGCAAAGTTGGCTG |
| 1121 | scaffold19700 | ACTCATAGGGGCCACAAACA | ATCGCATCTGCTTCGTCTTT |
| 1122 | scaffold34920 | AGATGAACGAGCCTCACGAT | TGCCAATGCCAAATGAAAAT |
| 1123 | scaffold20078 | AGGTCGCTAGATTGCTTCAGA | TGCACCTCGTTACAAACAGC |
| 1124 | scaffold20345 | AGTACGAGGCCTTTTGGGAT | TGATCTCTGCCACACACCTC |
| 1125 | scaffold7569 | AGTGCGTTCTCCTCGTTGTT | CGCCAATTCGTTAGGGAGTA |
| 1126 | scaffold19576 | AGTTAGGCCCTACACCCCAC | AGCGAGTGAACAAAGTGCAG |
| 1127 | scaffold6801 | ATAAGTGAGCCTGCAGACCG | TCCACACGGACCTAACATCA |
| 1128 | scaffold6759 | ATACGGGGACCAAACACAAA | AGTCACCTCCCACCTGATTG |
| 1129 | scaffold4809 | ATGTCGACGAAATTCCAAGC | GAATTGGCGGATCAAAAGAA |
| 1130 | scaffold21716 | CAAAAATGCCCTCGGATATG | TGGATGCCCAGTTCAAGATT |
| 1131 | scaffold470 | CAACACACACAAAGAGACGACA | TTATTGCCCAGCATGTGTGT |
| 1132 | scaffold7121 | CAACTTTTCGAGAGGCGAAG | CACCTCTCCTCTTCAGCACC |
| 1133 | scaffold13825 | CAAGCTTTGGAGTTTTTGTGG | AAAAGAAGCTGGAGACTCCTCA |
| 1134 | scaffold26085 | CAAGTGCTCTACCCAAAAACG | TGTTGGTGAGTTTGGTGCAT |
| 1135 | scaffold37692 | CAATTTTCAATTTTGATCTTTTGA | GAGGATCAAGTTGCGAGAGG |
| 1136 | scaffold31904 | CACCAGGCTAATCGTCATCC | CTGCAGTAGCCACATGGTTG |
| 1137 | scaffold14184 | CACCATCGTTTCTGTCCCTT | TAACCACACCTTCCGTTTCC |
| 1138 | scaffold13281 | CACTACGGTGTACATCACGG | GAAGGGTTTCAACTCCACGA |
| 1139 | scaffold19412 | CAGGTGGTCCCTTGTCTCAT | CTTTGGAAGTGTGGGGAAGA |
| 1140 | scaffold20585 | CAGTCAGTGGGGAACAAGGT | TCGTCTAGCTCCAACCGAAT |
| 1141 | scaffold22344 | CATGAAGGTCTCCAGGTGGT | GAAACAAGACTCGCTCCCAC |
| 1142 | scaffold28180 | CATGAGAGGGTAGGATTAAAGATGA | ATGAGGTGTTAGGTGCCTCG |
| 1143 | scaffold4691 | CATGTTTGTTAAAATAACCCGTAAAA | GGGTTGATGCAAAGATGGTT |
| 1144 | scaffold8491 | CCAAACCCAACTCCAAACAC | CGTACGCCTTTGTGGTGTAA |
| 1145 | scaffold5829 | CCACATTTACGCACTTGCAG | TCCGTATAATGATTTAACAACCCA |
| 1146 | scaffold3092 | CCACTTCATATGTTCCGGCT | TTGTGCACTCTCGCAGAAAC |
| 1147 | scaffold12204 | CCAGATGGAGGAGGAATCAA | AGAAGACAGCCTTGCTGTGG |
| 1148 | scaffold1189 | CCATATGCATGCAACAAACC | CATGGTTGAACCTGGCAGTA |
| 1149 | scaffold134 | CCATTCGGTCAAATCGAACT | TCTATTGGGGTGAAAGTGGC |
| 1150 | scaffold42892 | CCCCAATGAAGAACTCCTGA | TCCCTGAATAAAAACCGCAA |
| 1151 | scaffold48811 | CCCGGTTTGTAGTCCACCTA | TCCAAAAACAGAGAATCCCG |
| 1152 | scaffold34157 | CGAACAAAAACAACAAAGGGA | CTGTGGGTGGAATGTTGATG |
| 1153 | scaffold30953 | CGAATCGAAACCCTCGAATA | TCGGATAAAAATATCCGCATTC |
| 1154 | scaffold33253 | CGGATTAACTCAAAAGGGCA | AGCCTTTATTCCGCCTTCAT |
| 1155 | scaffold14184 | CGGTAATTGTTTTTGTGGCA | CCCTGCCATTAGTGGAAACA |
| 1156 | scaffold37913 | CGTACTGTCTCTTGTGCCGA | ATATTGCGAGTCAGGGTTCG |
| 1157 | scaffold35580 | CGTTAGCAAAACAATTTGGGA | TGATCAAAATGTTTCGTCAAAAA |
| 1158 | scaffold50507 | CGTTATCAAGGCTGTCCGTT | CCTTCGTGCTCTTATAGCGG |
| 1159 | scaffold37926 | CTCCAAGGATCCATCTCAGAA | CCTTCAGGAACCGTTGAAGA |
| 1160 | scaffold14947 | CTCTAAGGCCGTGACAAAGG | CTCTTGTGCAAATGGAAGCA |
| 1161 | scaffold16493 | CTGTACGCGTTGTATGTCGC | TGCATTGATGATGTGCATTG |
| 1162 | scaffold13757 | CTTGCAATCAAGCTCGAACA | GCTCACATACTGCCGATCAA |
| 1163 | scaffold42521 | CTTGCAGTCCTCCAACATGA | CCCCCTTCTTTTGTTGTGAA |
| 1164 | scaffold50643 | CTTGTTTGGTTTCGGACCAG | TGATGTCAATCAAGACCCCA |
| 1165 | scaffold6172 | CTTTTCGGTTTCTTTGCAGC | CAACGGTCCAGGGTAGTTTG |
| 1166 | scaffold27297 | GAAGGAACAAGAGGATGGCA | TCATGTTGTCGAGAATCCCA |
| 1167 | scaffold52112 | GAATCACAAAATCACGCCCT | TCATCAGACCAATAAGAGTCCG |
| 1168 | scaffold54408 | GAATGAACCCCACCAACAAC | TGAGGATTTTACAAGTCCTTCCTT |
| 1169 | scaffold18298 | GAATGTGAAGGTTCTCTCTGTTGA | TTTCGGCGGTTCTTAAAGATT |
| 1170 | scaffold11585 | GACGCCTTAACCAAACCGTA | AAAATGACTTCATCGGCCTG |
| 1171 | scaffold3855 | GAGAACCCTCGCAACAAATG | GGATCAATCATCCACGCTCT |
| 1172 | scaffold18455 | GAGCAATGAATTCCCAAGGA | AGAGTTTCCCTTTCTCCCCA |
| 1173 | scaffold10868 | GATAAGAACGAGGGGAAGGC | ACAGGCGAGAGGTTTGAAGA |
| 1174 | scaffold24523 | GCAACACCACGAATCTTTGA | GGGTTTCTCCAGTGCTAAGTTT |
| 1175 | scaffold43473 | GCAGCCTACAAAAACGCAAT | TTTTTGCAGGATCCGATGAT |
| 1176 | scaffold12663 | GCCAGAAAAAGTAAGCGCAC | ACGCCAGAGCTACACGATTT |
| 1177 | scaffold41297 | GCGAAGAACACGGAGAAGAG | CTCCCAAATTTAGCGGATGA |
| 1178 | scaffold28634 | GCGAGAGTCAACAACAAAAGC | TGTTGAGTTGGTACGGTTCG |
| 1179 | scaffold8032 | GCTAGCTCATGCCTCTGCTT | GGCCTACAGATCCTCTCTGC |
| 1180 | scaffold9403 | GCTATGGACATCCAGCCCTA | ATCCCAGGCCTACCTTCAAC |
| 1181 | scaffold31165 | GCTTACAACACCTTGGGCTC | GGTTGCTTTTGGAGATGCTT |
| 1182 | scaffold13616 | GCTTAGAAGCATACAATGGGC | CACATATCGTTTTTCCACCTTG |
| 1183 | scaffold39124 | GGAGTCAACGCAGACAATGA | ACCTGTAATCCACCCCACAA |
| 1184 | scaffold12255 | GGCTCAAGAGCTGAGTTGGT | CAAAAGCTTCCGGAAACAAA |
| 1185 | scaffold51602 | GGCTCTATTCCAATTTCCCA | CGATGTCTACGAAAGCACCA |
| 1186 | scaffold17598 | GTAGACCCGAACCAAACCTG | CGGTTTCGAATTTGTCAGTT |
| 1187 | scaffold4210 | GTTAACACCATCCGGTTTGC | CTTTTGCTCATTTGTTGCCA |
| 1188 | scaffold21777 | GTTGGTTTGGTTTGACTCGG | TGGAAAGCTTAAATGTTGTTTTGA |
| 1189 | scaffold38769 | TATCGCTCATGTTGCTGCTC | GAATGAATGGAAACGGAACG |
| 1190 | scaffold37191 | TATGCAAACCCAAATGGTCC | CCGGTTTCAAGATTCAAGGA |
| 1191 | scaffold11943 | TCAAAATTAGATATTCAACGTAAGAAA | GGATTACCATGGTTAGGGCA |
| 1192 | scaffold3952 | TCAAAGCTCGAAACTTCGGT | TAACAAACCTCGCAGCATCA |
| 1193 | scaffold3197 | TCAAGCAAGAGTTCAATAACAGAAA | GCTGGCCTAACACAACTGAGA |
| 1194 | scaffold23148 | TCACATTCACTTGGTGGAGG | TGATAATGCAGACTGGAGAACAA |
| 1195 | scaffold8463 | TCCAATCTCTCAGTGGTCCTTT | CTCACAAACCGCACCATAAA |
| 1196 | scaffold27962 | TCCTCACCTTGTACAACCACC | CCACCGGAGGAGAAGAAACT |
| 1197 | scaffold32064 | TCCTGCACCACCAAATTACC | TGCATGGCTAGTCCCAATTA |
| 1198 | scaffold12717 | TCGGTTAGGTTTCAATTCGG | CTGGGTTGGACATGAGGACT |
| 1199 | scaffold4533 | TCGGTTTCTCGGGTTAGATG | GAAGAAAGCAAGCACCTACCA |
| 1200 | scaffold24374 | TCGTATAACCAGCCACCCTC | TGCATTTAAACTTGAGTGGTTGA |
| 1201 | scaffold14606 | TCTCAGTAAACAAACGTGCAAGA | TCCGTACGAGGTAGGTTTCG |
| 1202 | scaffold13705 | TCTCCTTTCAGCTTTGGACC | TGGTCGTCAGACATGTGAGTT |
| 1203 | scaffold22758 | TCTTGATGGTCACGTTTTGG | GCCTCCCACTAGAATCTCCC |
| 1204 | scaffold62150 | TGAACACTGGTGAGACGGTT | TGATGATCGGAAACACGAAA |
| 1205 | scaffold35225 | TGATGCAAGAACCTCCCACT | GTCGACGACGAGGAAGATGT |
| 1206 | scaffold27059 | TGATGTGTTGTGATTGGTGTTC | AACGTTGTTGGTTTGGTCTTG |
| 1207 | scaffold17820 | TGCCATGACTGCAAAGAGAC | ATTCAATGGTCCAACCCAAA |
| 1208 | scaffold50374 | TGCCTTTTCGATAATCACACC | GCATGCATCTTCATTCTCACA |
| 1209 | scaffold7439 | TGCTTTTCTGTTCCTTGCCT | TTTCATCAGCTTCATCCACTG |
| 1210 | scaffold11786 | TGGAAGATTGGCTCTTGGTC | CACCTCAAATCCATCCGAGT |
| 1211 | scaffold4159 | TGGTAAAAGGCGTACGTCAA | CGGAAAAACAGAATAACTTGTGAA |
| 1212 | scaffold10986 | TGGTGAGGTGTGTACTTAGCG | CACTTCCACGACACCTGCTA |
| 1213 | scaffold7411 | TGGTTTGTGGGTTTTGGTTT | TGGACCAAGAGAACTTCCAAA |
| 1214 | scaffold35079 | TGTCGGACCACATGTTTTTG | GATGCAAATGGAGAAGGGAA |
| 1215 | scaffold48117 | TGTCTTACCCTGCTTCACCC | AACGACGAAAGAAAAAGGCA |
| 1216 | scaffold9668 | TGTGGCGGTTTACTTTTTAAG | ATGAGGTGATGGTGGCAAAT |
| 1217 | scaffold34679 | TGTGTTCACTTTGACTCGCA | CCATTTTGTCACCATGGACTT |
| 1218 | scaffold55016 | TGTTGATAGAAGAGTTGCCAAAA | GCAAACCTGATTTTGTCCTCC |
| 1219 | scaffold11609 | TGTTTGCGGTGGATCTACAA | GAGCTCCATAATGTGAAAATGG |
| 1220 | scaffold37232 | TTAGCGAGGAGGTTGCTCAT | GAGTAATAGAATAGCCGTTTGTTGA |
| 1221 | scaffold6648 | TTCTTTCGTCTCTTTGGCCT | TACTTCTCCGAGCCACATCC |
| 1222 | scaffold6292 | TTGAATGGTTTCTTGGCTCTT | CACCCATGAACCCCTTGTTA |
| 1223 | scaffold893 | TTGCAGTTCAGCTTGGTTTG | GGTCACCAACATGGATTCCT |
| 1224 | scaffold370 | TTGCATATCGCTCATTTCCA | TGATTGCCGGTTTGGTATTT |
| 1225 | scaffold38031 | TTGGAAAATCATTTACACCAGC | TGGAGCTTCTGGGACTCTGT |
| 1226 | scaffold4636 | TTGGAGCTTTCCATGTCCTT | TATCAGCAGCATCAGCAGGA |
| 1227 | scaffold20775 | TTTCAAGAGGAGAAGCGGTC | GATCCTCGGTGAAAGCTCTG |
| 1228 | scaffold21383 | TTTTATCTATTTAATCAGCTTTTGCAT | TGGACCCAGTTCTTATTTTTGG |
| 1229 | scaffold15667 | TTTTCTTTGGGACCAACCTG | GGTTGGTGTTGTAACCGGAC |
| 1230 | scaffold51081 | TTTTGTGCAGCTTCTGATGG | TTCTGCTCGGTCTTACCCTC |
| 1231 | scaffold42094 | AAAGCGTGGGTCTCATCCTA | CATGTTTGGGTTGTCACAGG |
| 1232 | C18422763 | AAATGCTGACCGTATCTGGC | GCCTTTCTTTCTCACATCGG |
| 1233 | scaffold2381 | ACATCTCCCAATCATGCTCC | CAAGCAACAAGCACGTCAAT |
| 1234 | scaffold18232 | ACATGTACATAGGCCCCCAA | ACGCCAACAAAGGAATCAAC |
| 1235 | scaffold10092 | ACCAGCAAGTTGGGATGAAC | GGCTGTCTTCTCATTGATTCAG |
| 1236 | scaffold13813 | ACCCCTGTTAAAAGAACGCA | GTTCGCCGAAAAACACAAAT |
| 1237 | scaffold13072 | ACCGAAGACATTTTGAGGGA | TGCAACACCATTCCCAAGTA |
| 1238 | scaffold15044 | AGAAACCGCTCCATTAGCTG | ATACATGTCCTGCACCGTCA |
| 1239 | scaffold24088 | AGAAACGCAAATGGTCTTCG | CTGTATGAGCATGGCTTCCA |
| 1240 | scaffold30650 | AGCTCTTCATGAACCGCATT | GTTGGCAGAGATGGGGATAA |
| 1241 | scaffold6939 | ATCAGTGCCCAATGTTCTCC | AGGCGTGTTAAACGCTCATT |
| 1242 | scaffold32075 | ATGGGAAGCCCCTCTTTTTA | AACAAAACCACATTTGATTCCA |
| 1243 | scaffold33222 | ATTGCTTTGCACTAACCGGA | CTCAAGACCAAAAAGCTGGC |
| 1244 | scaffold9866 | ATTTCGATACAAGGCGGTGA | CAGGCACAGTCACATCCAAC |
| 1245 | scaffold19674 | ATTTGACGTGCGATCGTGTA | AAACTTTCGGTTTGGATTTCTG |
| 1246 | scaffold13348 | CAACCGTTGATTGGATTGTG | CAAACGTCTCTGCACCACAC |
| 1247 | scaffold14159 | CACCTTCTAAGGCTAGTCCCG | ACCTTCAGTCGGCTGAGCTA |
| 1248 | scaffold43960 | CACTTCGCAAGAGCAAGTCA | TCACATAGTTGGGAGCAACG |
| 1249 | scaffold21663 | CACTTGCATATTTGTCAATCCAA | AACCGAACGGATAACCGAAT |
| 1250 | scaffold6302 | CAGCAATTCCAAGTTGGTCA | TAGGGACCAACAGCATGGAT |
| 1251 | scaffold20173 | CATATGGACTTGGGTGAGCA | CGAATGGACAGAGTAGATAAAGCA |
| 1252 | scaffold589 | CATGAGAGACTATTGCAAGGACA | GAAAGACACCCGTCAAGGAG |
| 1253 | scaffold20557 | CCATGGAAAACCACTCAAGAA | CGACATGCGCACTTAAGAAG |
| 1254 | scaffold20615 | CCCTTCTGCAAGGAGTTCTG | TAAACCATTCCCTTGGCTTC |
| 1255 | scaffold2832 | CGAAAGCAATAGCCTATATCCG | GCATGCATGGTTACATTTTCA |
| 1256 | scaffold9810 | CGCAATCTCAACACATGGAC | TTGATTGTTGAACCGGAAAT |
| 1257 | scaffold26895 | CGTAAGACGGCAACTCATCA | TCACATCCACCTTGTGTGCT |
| 1258 | scaffold11177 | CGTGTTTTCCTTTCTTATTTGTG | TGTTTGACCGGTCGTTGTAA |
| 1259 | scaffold32114 | CTCTGTTCGCAACAAACACG | CTTATGCCCAAAACACACGA |
| 1260 | scaffold63700 | GAAACGTGCTTCGTCCTCTC | GTCGGTCATGGACCAAGTCT |
| 1261 | scaffold21329 | GAAATGGAGTAAATATGTTACGGCTAA | TGCAGAGTGAAGTGGTGAGG |
| 1262 | scaffold53919 | GACAGTGACAGGTTGAGCGA | GGTGTAGTCGACGAAAGGGA |
| 1263 | scaffold17207 | GCAATGAGTAAGGTTGTTCTCG | TGACCGTTAGATTCACCAGC |
| 1264 | scaffold8052 | GCCCCAAGGCTTTCTCTATT | TTCAACAGTATCCGCTGGTG |
| 1265 | scaffold48088 | GCGCATGAAAATGCGTAGTA | TGACCAGCAGATTGGATTGA |
| 1266 | scaffold38338 | GCGCCAACGGTACGTAAATA | GGGACTATCGTCAGGAGCAG |
| 1267 | scaffold17028 | GCTAACCGTTGGTTGATAACTTG | TCACCATGAGGAGGTGCATA |
| 1268 | scaffold38532 | GCTGCGATCAGGAAGAACTC | CCTGATTTAGACGGAGAGCG |
| 1269 | scaffold57661 | GGATGGGTCGTTGTACTCGT | AAATTTTGTTCAAAACCGGC |
| 1270 | scaffold36621 | GGATTTCCAAGATGCAGTCA | CGGTGGTTCACCATCAGAAT |
| 1271 | scaffold3906 | GGCCCAAAACCCTAGTATCC | AATTCCCTTAAGGATGGGGT |
| 1272 | scaffold8595 | GGCTGCTTCCTCTGGTAGTG | ACCCATCTGAACTTTTGCCA |
| 1273 | scaffold4921 | GGTATCACTTGTTCTCCTGCG | CTACACGCGAGTGGACTGAA |
| 1274 | scaffold17854 | GTAAAACCCCCGTTCAAGGT | GAGGAGCAGCATGTGGAGTT |
| 1275 | scaffold35147 | GTGATGGATCGGTTTCGAGT | ATTCATGTACCACGAACGCA |
| 1276 | scaffold15735 | GTGCCCGAAAAGATTCAAAA | GGCCCAGCCTTGTTACATTA |
| 1277 | scaffold24714 | GTGTCGTCGATGCTTCGTTA | GACCTAGTGGGACACCGAGA |
| 1278 | scaffold14071 | GTTCGCCGCTATGTGTATCA | GCCAGCTCCTTTGAATTGTC |
| 1279 | scaffold46971 | TAAGCCACAGTCCTCGACCT | AGACCGTTGCCAAGAGTAGC |
| 1280 | scaffold4188 | TAGGCAGGGTTTGCTGCTAT | TGGTCTTTTGATCCCCTTTG |
| 1281 | scaffold12476 | TCAAACCCCTAAAAGCTGGA | TCTTGGTCTTTTCCAGCCAC |
| 1282 | scaffold2258 | TCCCCTTTTCTTTCTTGGCT | ATGCTGAAACTCTCCCCCTT |
| 1283 | scaffold13328 | TCCGATGGCGGATATAGAAA | TCCCTAGGTTGTCTTTCCTTCA |
| 1284 | scaffold22990 | TCCGCTTGTAAACTTCCACC | GAACCGGAACTTGAACGAAA |
| 1285 | scaffold18215 | TCGGGGAAAGCCTCTAACTT | CCCTGGTCCCAACCTTTAAT |
| 1286 | scaffold19024 | TCTTTGGATCTTCGTCGTCA | ATATGTGTGCCATCCTGCAA |
| 1287 | scaffold187 | TGAAAACCAATCCATGACAAA | GAGGGTTCGGAGGAGACATT |
| 1288 | scaffold50794 | TGATTGCAGTTCTTGAGTTTCC | CATACCCTCTGGCCGTGTAT |
| 1289 | scaffold2757 | TGATTGGAAAAATCTGTAAAATCTCA | GCGCTTTCAAAAATGGACTC |
| 1290 | scaffold5167 | TGCATATGCTGCTCTACTTGTTT | ATCGTATCATCGGATTTGCC |
| 1291 | scaffold4758 | TGGTGATGGTGGAGAAACAC | CCCTCTGTTTCGGTTTTACAG |
| 1292 | scaffold53632 | TGTCTTCCAAAACCAGGCTC | GGACTTGCCTTGATCGTCAT |
| 1293 | scaffold12239 | TGTTGTAACCAAACCACCTCA | TTGTGAATAACCACAGATTATTTCG |
| 1294 | scaffold122 | TTGACTGTTTGAGTTTCGATTCA | TGCACAGAGATCTAAGACACATCT |
| 1295 | scaffold13025 | TTGAGCTTGAGATGCGTGTT | TTGGGGATTCCATGTTTGTT |
| 1296 | scaffold25982 | TTGTCAGAGTCTCCCTCGGT | CGGCTCCACACGTTTAAGTT |
| 1297 | scaffold685 | CTTGATGGACAAAACCAGGG | CGCAGTGTTATCCCCAACTT |
| 1298 | scaffold401 | AAAAAGCCAAAGACTTTCAAGAGA | ACTGAACACAATAGCGCCAG |
| 1299 | scaffold5305 | AAAACTGAAATTCATCAGGAACG | TGGTGAGCAGCACATTGATT |
| 1300 | scaffold23267 | AACCAAGTCGAAACGCAAAT | TTACCTGAAAACCTTGGCGA |
| 1301 | scaffold6339 | AAGAGTCCTTGGCGATACCA | CAAAGAATGACAACATGGCG |
| 1302 | scaffold22208 | AATTCTAACGCGTTTGTCGG | TGCACTCCTTATTCGGCTTT |
| 1303 | scaffold34012 | ACAGAGGATGAACTCACGGG | TCTTCGACTCCACATCCTCC |
| 1304 | scaffold17483 | ACCCCATTCAACAAGTGCTC | ATGCTTGCTCCTTTGTTGCT |
| 1305 | scaffold13344 | ACGAGTGACAAGATCCCACC | CCCGCTCGCTTACTCTATGT |
| 1306 | scaffold19952 | AGACCGGCTTTCTGGAATTT | CGTAAACCGTTGGAACAACA |
| 1307 | scaffold52947 | AGCAGGAGCAAACGTTCATC | TCCTTAAAAATGTTGGTAGTGCAA |
| 1308 | scaffold41294 | AGCCCATCGCAATCTACAAC | TGGACCATCGAAGAAGACAA |
| 1309 | scaffold10333 | AGTTTAGTGGTGGACGCCAG | CGTCATTGAAACCCCTGAGT |
| 1310 | scaffold29198 | ATCATTCGCAGTTCCTCCAA | CAAGAAATCATGCAGGCGTA |
| 1311 | scaffold45720 | ATTGAATGCAAAGGAGTGGG | TTCTTCATTGTCGACGCAAC |
| 1312 | scaffold7882 | ATTGACCCTGACTCGAACCC | ACCAATTCTGTGGGATCAGG |
| 1313 | scaffold15658 | CAATGTTTTACCCGAGCTGAA | GCATTCCGTTCTTAGGTTTGG |
| 1314 | scaffold620 | CAATTGCAGCAACAGTAGGC | CACCCAGACGCTGTAACTGT |
| 1315 | scaffold10795 | CAGCCTGACAAGATAAAAGCA | CGTTTGGAGAATTTCACTTGG |
| 1316 | scaffold6470 | CCGAACCTGACTGACCCTTA | ATCACTTTGTGGGTGGATGC |
| 1317 | scaffold15781 | CCGACTTAGATGCCCATGAT | CGTTTTCGAACATGGGTTAAA |
| 1318 | scaffold13803 | CCTCTGTCCGCATATTTGGT | CTCTTGGACATATCTCCGGC |
| 1319 | scaffold6692 | CGGCCTAGACACCTCTTCTG | GAAAACGGCGATTAGTTTCA |
| 1320 | scaffold10397 | CGGTTTGTTCCTTGACGAAT | CCATCAGCAACCTCTACTTGC |
| 1321 | scaffold1767 | CTCCACAATACCAAACGGCT | TGGATTTGGCCTAAACTTGG |
| 1322 | scaffold12751 | CTCTTCCCCTTCCCATTTTT | TTAGGCTCACAACCACCTCC |
| 1323 | scaffold962 | CTTTGTGCCGAGATCCTTCT | AAAGCAGTCAAGAAACCGGA |
| 1324 | scaffold882 | GAACCCAAGGAAACATGCAC | TCAGAGTGTCCTCTGCCTCA |
| 1325 | scaffold3128 | GAGAGTGATGCGTTAATGGC | AGGGTTCCGTTTCCTTTGAT |
| 1326 | scaffold18245 | GATTCACCCTTCAATGCCAC | TGGCGAGAGTAGCTTCCAAT |
| 1327 | scaffold16761 | GCAACAGCCAGTGTGAAAGA | CGTCTTTGGAACAACGGAAT |
| 1328 | scaffold1712 | GCATCGACAAGGAACACAAC | CACCCGGTATCATGTTTTCA |
| 1329 | scaffold13125 | GCCAACGAAACGGTATTGAG | TTTCCATAGTTCCCATCCCA |
| 1330 | scaffold46267 | GTCCCTGTCAAAACGACAAA | GGAGCTAGGGCTTCGTTTTT |
| 1331 | scaffold26663 | TAAGGTCGCCTCGAGAGAAA | TTGACCAAACACCAATCCAA |
| 1332 | scaffold22664 | TCAAGGACACACTTCTCAGGTG | TTTGCTTCTGTTTTTGTTTGGA |
| 1333 | scaffold42321 | TCCTCCTCTGTCATTTTCCG | TCGAATTCATTTTCCACGCT |
| 1334 | scaffold17960 | TGCAGATTGATGGGTTGAAG | CCCTAAGGTCGTTTTCATGC |
| 1335 | scaffold2710 | TGGCAATGTGGGAAGTGTAA | ACGTGATTCATTCTCGACCC |
| 1336 | scaffold7490 | TGGGTGAAATTGCCTCTTTT | CCTCCCTAAGCAAGCTGGTA |
| 1337 | scaffold48462 | TGGTTGTTATTCCGGTGGTT | TGTTGCTGAGGCTGTTAAGG |
| 1338 | scaffold1342 | TTGACGCAATAAACAAGAATACAA | GGCCTCATACGATAACGTGG |
| 1339 | scaffold57362 | TTGCAGCTTCCTATCTGGCT | TGTCGAATTCTGGTGGAACA |
| 1340 | scaffold50932 | TTTTCGTGAAGCTGATGCTG | CCAGCTTTCTTTAAAATCCCAA |
| 1341 | scaffold6862 | TTTTTGGTACATTACTTGCTTAGATGA | TGCATGTAGTCAATTTCGGC |
| 1342 | scaffold33448 | ATCGGAAAGACTTCACCCCT | CAATCATGTCCCCAAAGGAC |
| 1343 | scaffold11971 | CACAGGGATCGAACTCCATAA | CGGTTCCTTAAAATATGGGTGA |
| 1344 | scaffold8925 | CACTGCTCTACGCTCTCTCTTG | CCAAGTTGGGCCATACAGTT |
| 1345 | scaffold26581 | CAGACATTGCCTCTCCAACA | AGGTAGCTGGACATGGATGC |
| 1346 | scaffold489 | CCCCCACCTAGTGAAACTCA | CCTCTTCCTCACCAACCAAC |
| 1347 | scaffold23610 | CGGATTAATGGATTCCGAGT | AAGCAAGCTCTCCCACTGTC |
| 1348 | scaffold5360 | CGGTCAAACCGAACTACGAT | GCCTTTGCGGATATCACATT |
| 1349 | scaffold53389 | CGTGCGGCTGAGAGTTTATT | CCCAAAGGAAATGCTTGAGA |
| 1350 | scaffold1577 | CTGCATATGAAGAGCGGACA | CAAGCTCCTGGAGACGAAAC |
| 1351 | scaffold4945 | GAGCATGTTCGACGGTGTTA | TTGGGGGTATCTCAACAAAAA |
| 1352 | scaffold32554 | GAGCCTTCGTCTCTCTCCAA | CAGCCTCCTCTGTTGAATCC |
| 1353 | scaffold23267 | GTACGACGGGCTCATCATCT | TTCGAATCCAGTGATCCTCC |
| 1354 | scaffold6562 | TAAGCAATGGTGCGAAACAG | GCGAGGGAACTAGTGCTGAC |
| 1355 | scaffold7406 | TAAGGGACGGTTTCTTTCCA | TCATGACTGGTATATGCTTAAAAGTT |
| 1356 | scaffold7801 | TACGGCTTGAAAGCCTGAGT | GCAGGGAACCACGAAATAGA |
| 1357 | scaffold29080 | TCCTATCCAACATGTTCCTCC | TCATCGATCTGCTTCTTTGTATG |
| 1358 | scaffold1651 | TCTCCATAGCCTCTGCCACT | ATGTTATGCTCCTCTCGCGT |
| 1359 | scaffold36199 | TGGAGATCGTACCCCTGAAC | TGTTGAAGTCTTGGCTGACG |
| 1360 | scaffold27053 | TCTGATAACCCCAGTTCCTTTT | ATTGTTGTCGATCGTGGTCA |
| 1361 | scaffold16247 | GAAAGAGATTTGCAGTGGGC | AAACCCTCCAATCCCAATTC |
| 1362 | scaffold16773 | AAACGCCCAAGGCTTCTTAC | CGGTGTTGTGAAATGGTGTC |
| 1363 | scaffold18232 | TCAGACAGCCTTGTGACCTG | GGATTGAGCTGCTTCTGGAG |
| 1364 | scaffold21243 | ATAGCGATGCTTCACAAGGG | TTGGAAGTAAAGCTGGGGTG |
| 1365 | scaffold26331 | TGTTTTGATCCTCCTCCTCC | TTGGAGAAAAATGGGTTTGC |
| 1366 | scaffold26546 | TCCAGGAAGGTGTCAGATCC | CGAACGCGCTCTCTCTCTAC |
| 1367 | scaffold27692 | GGGAGAAACAACAGAGAGCG | GAGTCGTTCTCTCGGAGTCG |
| 1368 | scaffold31652 | CCAGAAAGAGACTGGCTTGG | TGGTGTTTTGAACAACTGGG |
| 1369 | scaffold3832 | CCGCGATTCAATTTACAACC | TCGTTTCGGTCCGTATCTTC |
| 1370 | scaffold42309 | TTGTTCTGGTTTTGAGCACG | GAGAATGAGATGGGTCCAGG |
| 1371 | scaffold42393 | GCAAATCTTTCGAAACAGCC | CGAGAGCCTTTCTCGATGAC |
| 1372 | scaffold4482 | TCCGAATAGGTGAGGTGGAG | GCCTGGCGAGAGATTAAGTG |
| 1373 | scaffold45406 | TGGTTGATGCTTCAGACGTG | TGCAAGCAGTGAAATGGAAG |
| 1374 | scaffold46974 | GCTTTGTCTTTTTCCCCCTC | TGTTGTCGTTTGCGTCTCTC |
| 1375 | scaffold47902 | CCTCTTCAGTCGAGGTCTGG | GCGATGATGATGGTGTTTTG |
| 1376 | scaffold53538 | TTGTTTCCCTTTTCCATTCC | ATGGCCAAAAAGTCAACGAC |
| 1377 | scaffold5459 | GGTTAGGATTCTTCACCTGTGG | ACTGGACGACCTTCACATCG |
| 1378 | scaffold5524 | CAAAAGGCAAAGACCGAGAG | CCACTGACCTTGGATGTGTG |
| 1379 | scaffold56189 | TAAGTGCCTGCATTTTGCAC | GCCATTACCGGCTTGTACTC |
| 1380 | scaffold6602 | AAGGCCAGTCCAACCAGAG | GATAACCGATCGGACATTCG |
| 1381 | scaffold9840 | CTCTTCCTCCATGAGCCTTG | AAATGTGCAAATCAGATACATGG |
| 1382 | scaffold10864 | TCACAGGAGACACCCCTACC | AACCAAAGCTGATCTCGCAC |
| 1383 | scaffold2153 | TACTTACCCCTCTGCATCGC | CACCTGCCAATTATGCACAC |
| 1384 | scaffold46224 | TTGGGTGGACCTAGTTTTCG | AGATGGAGGAAGCTCTCACG |
| 1385 | scaffold11085 | TCAAGAGGGTAATCAACGGC | GGTTCTTCCACTCCTCCTCC |
| 1386 | scaffold16438 | TGGGAAGTAAGTCAACATGGC | TTCTCGGTGGAGTATCGGTC |
| 1387 | scaffold7889 | GCTGGGGAGAGAGAGAGGAG | TAGGAGTGTGGCCCACTAGG |
| 1388 | scaffold11085 | TACGACCAAGACGGGAAGAC | CGGTGAACTCTTCATCTCTCC |
| 1389 | scaffold13164 | AAACCACGACTCCCTACACG | TGGTTGTTGCTCTCTGTTCG |
| 1390 | scaffold17119 | CTCCTTCTCTTTCACGCACC | AACACTGAACTCCCCACCAC |
| 1391 | scaffold1865 | TTTTGATCCGTTTCCGACTC | CGCAATCCACACAAACAGAC |
| 1392 | scaffold20522 | CCAGTTGAGTCAGAGCTCCC | ATTGCAGGGGTTGTATCAGC |
| 1393 | scaffold21076 | CTTCTGGCTTCAGATTTGGC | CATTCTGTTCACCGTCATGC |
| 1394 | scaffold25012 | CTTGTCCTCTTTCTGTCCCG | ATGAACGAAACCGGATTGAG |
| 1395 | scaffold26424 | TGCGTAGAGAATTGGAGAAAAG | TAGGGGGTGCTATTGGTTTG |
| 1396 | scaffold26630 | ACAAAAATGTCTCCGATGCC | TGCCTCGTTACTTGACATGC |
| 1397 | scaffold30303 | TCGTCTGCCATGTCTCTCTG | CAGCTGATATCGTATGGTGGG |
| 1398 | scaffold39628 | GTAACCACCAAGGCCTGTTC | ACAACTGATTGTCTTGGGGC |
| 1399 | scaffold4125 | GGGCGATTGTGTTATAGGATAAG | TGTCTTCGACCAAACTCGTG |
| 1400 | scaffold4953 | ACGCATTCTACGGTGTCTCC | ACTCATAGCCACAACTGCCC |
| 1401 | scaffold4953 | TTCTGCCGTTTTCTGGAATC | CACACATACGGGTCACAAGC |
| 1402 | scaffold5137 | TGCAAGCATACAGGTTCGAC | ACATTTGCTGAAAAGGTGCC |
| 1403 | scaffold6439 | TGGGATCTTCGTTCTTCATTG | AGAGAAGTGGAATGGGCTTG |
| 1404 | scaffold11142 | AAAGCGTCAAGCGATTATGG | TTGCAAATCCTGTAGAGGGG |
| 1405 | scaffold12201 | TGGCATGTTTTTACTCTGCG | TAAAGGGATACGCATGGAGG |
| 1406 | scaffold12898 | ACTTGTGTATGCCTGGAGGG | TCGGTGTGGCAAGTACAAAG |
| 1407 | scaffold14785 | CCACCAATAGGAAAGGCTTG | ACGTAGAAGATCCCCAGACG |
| 1408 | scaffold17759 | GAATCGGTTTTAGGGGCTTC | AATTGTAAGAATGAGAGATTTGAGC |
| 1409 | scaffold20498 | CCTATCGTTGAAGCCTCCAG | TTCTTGGTATTTTCTCGGCG |
| 1410 | scaffold20942 | CCTTTTGGTTGCATAAACCC | TGCTCCAGTCCTATGAACCC |
| 1411 | scaffold23173 | GGCTCTCTAATCCAATGAAAGG | TATTTCTTCTTGGGATGGCG |
| 1412 | scaffold27772 | TTTGCGTAAAGCTTACGTAGTTG | CGGCTGCATTATGATTTTTG |
| 1413 | scaffold34151 | TAGAACCCGGATTTTTGCAG | AAGGCTTGCTCTTCTTGTGG |
| 1414 | scaffold34308 | GACGAGTGGGCAGGTAGAAG | ATAAGTGGCATGATTTCCCG |
| 1415 | scaffold3728 | CGGGAGAAAATGTCGAGAAC | CCTTATTTGGCCTCTCTCCC |
| 1416 | scaffold43636 | CAAAGAGGCAACTCCCAGAG | TATTAGGGTTCAGAGGGCCG |
| 1417 | scaffold47207 | ACGCTAGGCAAAGAGCAGAG | ATGCTTAGGCTTGTTGGGTG |
| 1418 | scaffold54249 | TGCATTCAGCATATCTTGCAG | CGAAGTCTGTTGACCGAATG |
| 1419 | scaffold6862 | TATATGCAGGTCTATGGCGG | TCGAATTTTCCTAGTCTTTTTCG |
| 1420 | scaffold14107 | TATTTCATCCGACAGTGCCC | CATCGTCGGCCATAACTTTC |
| 1421 | scaffold18893 | CGGAAACAAGCATCTTCACC | TCTCTCGTCTGCCCTACCTC |
| 1422 | scaffold23100 | ACGCGGATTCAAATCAACTC | CGAGGCTCCGTTCTATTGAC |
| 1423 | scaffold41124 | TTCCAAAATCAGCCAGCTTC | AGATGGGCAACCAATCAATC |
| 1424 | scaffold5911 | TTGCCCATCGCAATATTTTC | TAGACTTTCCCACGTTTGGC |
| 1425 | scaffold62433 | TCATCTGTTGTCCTCTCTAGATGC | TCATGTCTGGATGATCTCGC |
| 1426 | scaffold6817 | TTATATGGAAACGGAAGCGG | GATTCCGTTTTGGAATCACG |
| 1427 | scaffold13111 | ACCATGAACAAAGCTCGGTC | AAATCTCTGCCATTGCAACC |
| 1428 | scaffold13651 | CAGCGATTCCATAGTGCAAC | CGGGTGGTAATCGTGATGTC |
| 1429 | scaffold16020 | TGGGAAGGATCTCAGGTTTG | TTGGCCAAACCGAAAAATAG |
| 1430 | scaffold27019 | AGGGAAGCAAACAGATGTGC | TCCGATTTAATATGCGGAGC |
| 1431 | scaffold30082 | TGCGTATTGGATTTGTGTTG | AGGAATCGAAAACGGATGTG |
| 1432 | scaffold33883 | TGTTTGACGAGTGAGTTGGC | TACAGGAGTGGGGATTGCTC |
| 1433 | scaffold8603 | AAAGATGCTAGCACAAGGGC | GGCCTTCCACTGTCAATTTC |
| 1434 | scaffold928 | GTTGCATGCCACAATCACTC | TTTCACCACGACAACACCTC |
| 1435 | scaffold12771 | TCTCGAAGTCGTTATGATGCC | CTTCGCTCAAAGCCAGAAAC |
| 1436 | scaffold27319 | TGTGGCATTTCGATTCTGAG | AACACATTTCTCGACCCTGG |
| 1437 | scaffold3700 | GCATGCTCACACGAGTTTTG | AACACACACCTTTTGCGATG |
| 1438 | scaffold40211 | GTAGGCTGCTCTCAACCGTC | AGAAACGACGTGACTGGACC |
| 1439 | scaffold4728 | GGTGCACACCAATGACTACG | CATGAAATGCTGAGAGAAAAGG |
| 1440 | scaffold9248 | TGTAACACCCATAATGAAGTCG | GAGAGCTTCTGGGTTCGTTG |
| 1441 | scaffold28171 | CGAGGAAGAGGAAGAAGACG | AAAGGAAAAATCTCAAACCCG |
| 1442 | scaffold1136 | AACCAAACAACAAGCCAGGT | GCAGGAATGCGAAACCTTTA |
| 1443 | scaffold36710 | GCAATGGCTGTTGTCTGAAA | TGGCATTATCCTACCACAAAAA |
| 1444 | scaffold23518 | CCCAAAGCCTCCTTCTCTCT | GAAAACGAAGAGGTGTCTATAAGAA |
| 1445 | scaffold27871 | GCAACGTAGCGGAGAAGAAC | AACACATTGCCCAAAGGAAG |
| 1446 | scaffold52406 | TCCCTCGAAGCAGTACCATC | ACTGTGCTCATGATCGAACC |
| 1447 | scaffold31784 | GGGTTGACAACAATCTTGGAG | GGCCCAAATACAATCCACAC |
| 1448 | scaffold8844 | CCTTCACCGTTTATGCAGGT | ACTTTTCAGCAGGGCTTTGA |
| 1449 | scaffold6273 | CTTTCCGACCAAACACCAGT | CCCAGTTCAGAGCCTTCTTG |
| 1450 | scaffold8844 | ATCCCATGCACCAGTAGCAG | TGAGACCCTTTTTCTGGATGA |
| 1451 | scaffold6225 | CGCACTAGTGACCTCGATGA | AGCTTCAAGTTCAACGGTTTG |
| 1452 | scaffold21319 | GGTGATCCTCCGTTTGAGAA | GCATTCTTCTGTCTCTCGCC |
| 1453 | scaffold27691 | CGCAACCATGTGAAGAAGAA | TCTACACTGAACCCGAAGGAA |
| 1454 | scaffold51659 | AAAAGCGAAATGAACCAACG | TCTTCGAGAGGAGAATAAGAGTTGA |
| 1455 | scaffold669 | GCATCTCGATTCAATCCTCC | TGCTCACACCCTCTTGATTTC |
| 1456 | scaffold4343 | CGGTACGGCGAATAGAAAAA | GTCCCCGATGAAACAGTGAT |
| 1457 | scaffold5152 | TCGCCACTTACTCTTTTGGG | TCGCACTCTACATGCCTCAC |
| 1458 | scaffold37931 | TCAACGACCAGGAGCTTATTTT | TTTGGTATTTGGGCTTCTCG |
| 1459 | scaffold49437 | ACATGCGGGATTTTGAAGAC | AGCATCATCGACCCTGAAAC |
| 1460 | scaffold4001 | GGGAAAGAGAGAAGACGGAGA | CTCTTCCCTTCAGCCTTTGA |
| 1461 | scaffold12012 | AACCGTCACAGATCCTGTCC | TGCTCTGCAAGATATTCCCC |
| 1462 | scaffold22174 | TAATCAAAGACCGCCGCTAT | CTGAGCTGACCTTGTAGCCC |
| 1463 | scaffold6435 | GGCAAAATCACTTCGCAAAT | CAGACCTCGAAAAAGGCTTG |
| 1464 | scaffold9711 | ACGAAGCGATTAACGGAGAA | ACACGCACCGCTTCTTACTT |
| 1465 | scaffold1329 | GCAGCAACAGCGATACCAAT | ATGTCTTTGCTGCGGTCTTC |
| 1466 | scaffold18865 | GATCTCCCTGTCGATTGCAT | GCTTTCCCAAACGATCAGAG |
| 1467 | scaffold503 | ACACCGGAGCTTAGCAAAAA | TACATCCCCATTCCCTACCA |
| 1468 | scaffold8664 | CCTATCCATCCAGCGACAGT | AAAATCTCCGACTCCCCAAT |
| 1469 | scaffold26515 | CCTTACTGGCTGCAGCTTTC | GTAACATCCCGGTTTGTGGT |
| 1470 | scaffold34248 | GCTTTTGACAGAGAGGACGG | ACGCAACAAGATGAGTGTGC |
| 1471 | scaffold11676 | TTGGTGAAGCGGTAGCCTAT | TGAAGTCACCCTCAACCCAT |
| 1472 | scaffold23237 | TTCAAGATTCGGAGACCAGAA | CTCCGAGCTTCCATCATCTC |
| 1473 | scaffold10172 | AGAAAGAACACCGACGCACT | TAGCCAGAGCAGTTTCAGCA |
| 1474 | scaffold1877 | ATTTGAAAGCTGGTCGAGGA | CACCGATCTCCTTTGCATTT |
| 1475 | scaffold10590 | CACATGGAGTGATGGCAAAC | CATTTCAACCACCAACATCG |
| 1476 | scaffold14526 | CCCATCGTGGATGCTCTAAT | TGCCACGACAAAAATCGTAA |
| 1477 | scaffold29976 | CCTGCGATGTGAAGAAGACA | GAACCAGTAGCTTCCCTCCC |
| 1478 | scaffold10251 | CTATTCAGCTCCAAGCCCAG | TCATCATTCCCTCCAACCTC |
| 1479 | scaffold2110 | CTCCTACCCCTCTAAACCGC | GCCAATTACCCACACTTGCT |
| 1480 | scaffold45567 | CTCTGGTTCTCGAAGCCTTG | CCTTGATCATGATTTGGGAT |
| 1481 | scaffold62827 | GAGGAAAGAGAGAGTGGGGC | CGCCCAAAACTTAGTCCAAA |
| 1482 | scaffold566 | GATCGGGTGGCTCATTTTTA | CTTTCGAAACTTCCCAGCAG |
| 1483 | scaffold46233 | GGCCGTGAGAATCTTCATGT | CTTGAGGCTTTGCTACCGTC |
| 1484 | scaffold4304 | GGGCGTATCAGGAAGAGTGA | ATGTGTGCCTGGGAGAAAAT |
| 1485 | scaffold14806 | TCCCCAAACTCGAACATCTC | CACCCCTCTATATCCACCCA |
| 1486 | scaffold14184 | TGAGATGGTCCCTGAGAACC | CCCTTTTCCCCTTCTATTGG |
| 1487 | scaffold4884 | AAGGCACCTCGAAAGTCAGA | GCAGCAGAGCTATGGTTTCC |
| 1488 | scaffold14551 | CCATTTGGTAAGCCCGTAAA | AGCATCATCGACCCTGAAAC |
| 1489 | scaffold18342 | CCCGACTCAATTGTGGACTT | AAAGCGCATTGCCTACACTT |
| 1490 | scaffold7345 | CGTTTGGGCTATTGGTGAAT | ATGCAACTGCAAGTCCACAG |
| 1491 | scaffold26359 | GAAACACAAACGCAGCAGAA | GACTCTGACCTTCCACGAGC |
| 1492 | scaffold2257 | GACGACACATAATGCAGAAACAA | CTCCTCCTCTGCTCTCTCCA |
| 1493 | scaffold39594 | GGAATGAGAAGAGCTCGGTG | GATCTCCGACGAAAACCAGA |
| 1494 | scaffold8659 | GTTTGACGCGGATAACCATC | AATCTTCGCATACCCATTCG |
| 1495 | scaffold13854 | TAAGGAGCCACCGAGAGAGA | AGAGCCGTATGTTCGTGGAC |
| 1496 | scaffold24088 | TCGTCAAGCACGTCAGAATC | ATTCGTCAACCACACCATCA |
| 1497 | scaffold33321 | TGGATTTTTATCGGTGGGAA | CTCCAACGAACTCCAACTCC |
| 1498 | scaffold5448 | TTGACACAACGTGGAGGGTA | TCGATCTTGAACCCAGCTCT |
| 1499 | scaffold30472 | TTGGGGTTTGGTTTTGTTTT | GCATGCTTTTGCTGTGTCTC |
| 1500 | scaffold6272 | AAAACTGGCATTTGGTTTGG | TGCTTTACGCTCTTGCTTCA |
| 1501 | scaffold11379 | AAAGGGTCCCTATTGGGCTA | TTATCCCTATGTTCGCAGCC |
| 1502 | scaffold15731 | AAGAAGCCAGCGACTGAGAC | GCACCAAAGCTTAAGCAACA |
| 1503 | scaffold140 | AAGACCAACCATGACTTGCC | AAAACGACTATCTCACGCCG |
| 1504 | scaffold7080 | AAGCTTCGAGGAGAAGACCC | AGGATGTTTCGTCGGTTACG |
| 1505 | scaffold37571 | AAGGAGGATCACGAGGAGGT | CATGGTAGCATCGAAAGCCT |
| 1506 | scaffold38186 | AAGGGAGAGTACAAGAAACACCA | CATGGTTAAAACAATGGCCC |
| 1507 | scaffold29081 | AATGTGAGGCTGTTTGGGAC | GGGTTGGATTTAAAGGGGAA |
| 1508 | scaffold6828 | AATTTAAAACTGGCCCGTGG | TCGTCGCAGAACTGAATGAC |
| 1509 | scaffold19213 | ACCCCCACGATGTTTGTTTA | TGAGATTGCTCAATTTGCTGA |
| 1510 | scaffold25997 | ACCCGAACTGCAGTAACACC | TGCCAAGTTTTGCCTCCTAT |
| 1511 | scaffold13369 | ACGGATGTGAGGAGATCAGG | TGATGCTGTCCAGGTACCAA |
| 1512 | scaffold342 | ACTGGTGTGTGTCGTCATGG | TGTATGGGGAGAAGAGGGAA |
| 1513 | scaffold3254 | AGACCGGGAAGTGACCTTTT | ATTGCGCCCTTATATACCCC |
| 1514 | scaffold9473 | AGATTCGACCCACGAACAAC | CGTTGCTCCACATGATAACG |
| 1515 | scaffold9774 | AGGAGTCAAGCCAGCGAGTA | AAACCCTAACGGCTTTTGGT |
| 1516 | scaffold45567 | ATCGCAGATCGCAATTTTCT | AGACCTGACCTGCTGTGACC |
| 1517 | scaffold1110 | ATGTCGTATGGCTTCGGTTC | GATAAAAAGAAAGCCGCCAA |
| 1518 | scaffold11191 | CAAGCTGCATCCATCCATTA | ATGTCATGGAGCCCTCGTAG |
| 1519 | scaffold9629 | CCAGGAATCTCAAGACCCAA | ACACACATGCCGTCTCTCAG |
| 1520 | scaffold15507 | CCATTGTCGAAGTACAACTGTG | TGTCCGCGATTTTAAGGTTC |
| 1521 | scaffold36132 | CCTCTCGCTCCTAAACCCTT | GAGAGGATTGACGCCATTGT |
| 1522 | scaffold21329 | CGAAGTTGCAGACAAGGTGA | ATGACGTGAGATCCTTGGGA |
| 1523 | scaffold1163 | CGATCACTTGGATTCGGTCT | CGCGATTTTACGGAGAAGAG |
| 1524 | scaffold30283 | CGATCTTCTCCTCTCTCTCCAA | TGTGACGATCAACCATTGCT |
| 1525 | scaffold19756 | CGGAGATGGAATCTCACCAT | CTCTCGCTTCCTTCCTTCAA |
| 1526 | scaffold3760 | CGTCCGAATTTGCTTTCTTC | CCAGAGGCGATTTCGAGTAG |
| 1527 | scaffold31312 | CGTCTCCGACTCCGATAAGA | AAGCCTCTACGCAAACGAAA |
| 1528 | scaffold6321 | CTCAGCATCAAGCAAAGCAG | AAGCCATCCTTGCTCCACTA |
| 1529 | scaffold6978 | GAAAACTTTGCTTTGGAGCG | CAAAAAGAAACTATGGTCACAACC |
| 1530 | scaffold13026 | GAATTTGACGGCGAAGAGAG | CCAAACGCAGATTCCTTCTC |
| 1531 | scaffold49669 | GACCATATCATCGGGCGTAT | GGAGGACGATCGATCTACGA |
| 1532 | scaffold19396 | GCAACTCAACAGTTGGGAAAA | TAGGCGCCAATGTTAATGCT |
| 1533 | scaffold3328 | GCAATCAAGCTAGAACAGCG | TAGCGTGAAGGAAGGAAGGA |
| 1534 | scaffold19770 | GCACCCTGTTGATGGATTCT | CACTGCAACGTCACCAACTT |
| 1535 | scaffold12381 | GCAGTGATGACCACTCTTTTCA | GTTCCATATCGCTGGCGTAG |
| 1536 | scaffold36540 | GCCAATGTTGTTTCGGTTG | CTCCTCCCCATAGAGGGCTA |
| 1537 | scaffold15480 | GCTCCGATGATCACGATTCT | CTTCATCCCCTCACCACACT |
| 1538 | scaffold10658 | GCTGGTGTAGATTCCGGTGT | ACTCTTTCTCCGCCGCTACT |
| 1539 | scaffold15164 | GGAAGGCTTGTCAACTGCAT | TTTTTGGACAACTGGTTCACAT |
| 1540 | scaffold124 | GGACTAAGGGGCATAAAACCA | TGCATGATATTCACACGGCT |
| 1541 | scaffold4425 | GGAGATGAGAAGCTTGGTGG | CTCTGGTCTTACGTCCTCGC |
| 1542 | scaffold64040 | GGCGAGAGAAAGAGAGAGATCA | AACGCAACTCAAAAGATGGG |
| 1543 | scaffold7907 | GGCGTTGATGTTGCTCAGTA | TACACACCATTCCCTCCCAT |
| 1544 | scaffold8206 | GGGACGCAAAATTTACGCTA | CTTGCCGTGACCCAATAAGT |
| 1545 | scaffold8229 | GGGCGTCTACTTCTCTGTCG | GCAGGGAAGCAGCAAAATAA |
| 1546 | scaffold44037 | GGGGAGAGAGAGAAGAGTGTGA | TGGTTTGGAGAAGACAGAGACA |
| 1547 | scaffold9794 | GGTTTTGTCTTTGCGCTGTT | GTCAGAGACCCAGAAACCCA |
| 1548 | scaffold18619 | TAACCTGCGGGATTTTGAAG | AGACCGGAAGCATCATCAAC |
| 1549 | scaffold1677 | TACCCCTCTGCATCTTCCAG | GGGGCTTGGTATACATTGGA |
| 1550 | scaffold40834 | TCACATCGTGTACCCATTCA | TCGTCGAAAAAGAGAGAGCC |
| 1551 | scaffold56189 | TCGATCTTGAACTTACCGGG | TACTTCTCCGCGTCTTCCTC |
| 1552 | scaffold20902 | TCGCAATACAGAGATGACGC | CGCGATATGCTGATTCAAGA |
| 1553 | scaffold33665 | TGAGAGGAAGAGAAAAGCCG | TTGTTAAAGAGACAAGGGGAAA |
| 1554 | scaffold28867 | TGCAAGTGAACATCTTTGCC | TCGGATTGTGGATCCCTTTA |
| 1555 | scaffold35265 | TGGATAAGTGTAGGACCGCA | TCCGTCGTCTTGTTCAATCA |
| 1556 | scaffold57712 | TGGGAGAAACATGGGAAGAG | CGCAGCTTGTTCTCTCAAAA |
| 1557 | scaffold43018 | TGTCACTCAATCGTCTCCCA | CGAAGAAGCTCCTCCTCAAA |
| 1558 | scaffold26152 | TGTCATCACCCCTGATTCAA | TGCTCATGGATGAATACGGA |
| 1559 | scaffold7353 | TTAAAGAGGGAGGCTGCTGA | TGATGTGCTTTGCACAGAAAG |
| 1560 | scaffold42320 | TTCATCACACACTCTCACCGT | TTGTCGGATGAGAAACTCGAT |
| 1561 | scaffold7238 | TTGTCAACTCGCCATCTCTG | CAACAGCGAGCATACCAAGA |
| 1562 | scaffold7106 | AAACGGATCCCTAATCACCC | TTTACTAAATCCGTCGGCCA |
| 1563 | scaffold11843 | AAATTGCCGAAACCCTAAGC | CGACGAAAACCCCTCTGTAA |
| 1564 | scaffold22701 | AACGGCGAACTGTATCTTCG | GTGGAGGGAGGGAAATTTGT |
| 1565 | scaffold4294 | AAGAGACGCCATGATTGACC | GGAACACGGATCCTAACCAA |
| 1566 | scaffold18830 | AAGCGGCACTCAACATAAGC | TAACCGTTTCTTGCTTGCCT |
| 1567 | scaffold12032 | AAGGATTCGGTTAGCTTTCCA | CCGTGTAAGACTTGCTTCCC |
| 1568 | scaffold8345 | AAGGCCAGCGTTGATTAAGA | CTCGAACCCAGTATTGCACA |
| 1569 | scaffold54845 | AAGTTCGCCACACCTTCTTG | GTCCCGAATCAGATCGACAT |
| 1570 | scaffold1493 | AATACAATACGACGGCGAGG | CTTGGAAGGGAGGGACTTTC |
| 1571 | scaffold13220 | AATACCGGAAACACCCTTGG | TTGTCCCTCTTCGCTCACTT |
| 1572 | scaffold27011 | AATAGCCACCAAACGCAATC | AGTGGTGGGTAGAGAGCGAA |
| 1573 | scaffold19424 | AATATGGTATCCGTGCCTGC | GCTGGTCCATTCCAAGATGT |
| 1574 | scaffold17181 | AATCGAGTTCTTTCTCGCCA | CGAGAGAGCAAACCAAGTCG |
| 1575 | scaffold18639 | ACCCCAAACTATCCCCAAAG | ACTCAAAACATGGCGGTTTC |
| 1576 | scaffold21894 | ACCTCTGCCTCCTCCAACTT | TTGGTGTGATTTTGCGTGTT |
| 1577 | scaffold11531 | ACCTTCGTACCAACACCCAA | CTCCTGGAACCCAACAAAAA |
| 1578 | scaffold40249 | ACCTTTTCGTCAGTCATCGC | TCTTGATCACCTTCTCGGCT |
| 1579 | scaffold17281 | ACGTCAGGTCTCGTGCTTCT | AAAGAAGGGGTGGATGTGGT |
| 1580 | scaffold19424 | ACGTGAGCTCCAATTCAACC | CTCCGCTTCTTCTCTCTCCA |
| 1581 | scaffold17710 | ACTATCGGCGGTAAGGGTTT | CAATCCTCCCAATCCTCTCA |
| 1582 | scaffold13180 | ACTATCTGGCCAACACCGTC | TTTTTGGTTTTCCTCAAGCA |
| 1583 | scaffold13985 | ACTCGTTGCTGCTGTGTGTC | GCGAAGCCAATTGAGAAAAG |
| 1584 | scaffold27067 | AGAAAACACCGTCCTTGTCG | TTCTCCATTTCCACCACACA |
| 1585 | scaffold25231 | AGAGGTGTGGATGAGTGAGGA | CCCTCAAATCCATCTCCAAA |
| 1586 | scaffold13201 | AGATTTCGAGTCGGCACCTA | AATTGAAGAACATGGCTGGC |
| 1587 | scaffold22160 | AGCCACCAAGTCCTCTTCAA | GCTTGTCTTGGGGTTTGGTA |
| 1588 | scaffold34135 | AGCCTCGTGTCCCTCCTAAT | CATCCACTCCATTTTCCACC |
| 1589 | scaffold5260 | AGCCTGAGAAATAGGCGACA | AGAAACGAGAGGAGCCAACA |
| 1590 | scaffold28180 | AGCTCGGAGGTGTTCGTAGA | CCAACAAACAACACACTCGC |
| 1591 | scaffold3251 | AGCTTCACTGCGAAGGAAAA | TCTTTCCCATTTTCACAGCC |
| 1592 | scaffold28408 | AGGACCCAGTCCTTGGAGTT | TCCAGAAATTGGCTTTGCTT |
| 1593 | scaffold17169 | AGGAGGGATCTGGAAGTCGT | TTGCTCTCTCACGTCACACC |
| 1594 | scaffold18548 | AGGGGAAGGGAAATGAGAAA | GCCTAGCGCTAAAGTGATCG |
| 1595 | scaffold36971 | AGGTGATCTCACCAACAGGC | GGGGTGGGTAGGTTGACTTT |
| 1596 | scaffold39743 | AGTTCAACACGTCCGACACA | AACGGAAGAGGTGAAGCAGA |
| 1597 | scaffold3200 | ATAGAGGACCGACACATGGC | CGCGGGTTTTGTTTTAACTT |
| 1598 | scaffold5479 | ATATGGCCCTGCAAACAGAG | CAGCTTATGGAATGCCCCT |
| 1599 | scaffold42639 | ATATTCCGAGCACGATTTGC | GCAGGATTCTCTTGGTGGAA |
| 1600 | scaffold10312 | ATCAAGACCTCGGCTGCTTA | CCGGCTTTTGCTCTTCTTCT |
| 1601 | scaffold30921 | ATCTCGAATCGCATTGATCC | GCCCTCGTGTCAAATCCTTA |
| 1602 | scaffold423 | ATTCAACATCGCTCACCTCC | GTGAAACGGCCTCATACCAT |
| 1603 | scaffold34252 | ATTCTTCGTGAAGACGGCAC | TGAAGCAAACAAATGGGAGA |
| 1604 | scaffold1244 | ATTGATGCAGGATGCGTACA | GACCCAGAAACCGCAAATTA |
| 1605 | scaffold12474 | ATTGATGTTGGCCTCTGCTC | CGCAGTATTGCTCTCCAACA |
| 1606 | scaffold17564 | ATTGGCTTGGTAGCTGTCGT | AATGGGATGCACAGGGATAG |
| 1607 | scaffold4943 | ATTTGGGGTTCTCATTCACG | TCAAGTCCCTCGTCTTGGAA |
| 1608 | scaffold12047 | CAATCTCCAAACCAGCCACT | AAGATTCCTGCGATCCTCCT |
| 1609 | scaffold30847 | CAATTTTTGGACATTTTTCAGGA | TACGCCACCTTTTACGTTCC |
| 1610 | scaffold10232 | CACACGACAACCGCTGTTAC | TTTTAGGATTGGAGGAGGCA |
| 1611 | scaffold8584 | CACGAGTCGTCCCCATACTT | CTCCTCTGATCGGCCATTTA |
| 1612 | scaffold20459 | CACGATTCTTTGCTTCTCTGC | ATCCTCACCCGATTGTCTTG |
| 1613 | scaffold15653 | CACTCAGAGGAGGAGGTTGC | GCCACGTGTAGGCATGTAGA |
| 1614 | scaffold908 | CACTCCTACCACCTCGCTTC | GATTCCTCCTGCAAGTTTGG |
| 1615 | scaffold1197 | CACTCTCCATTCCTCCTTGC | GGTGGCTGCTATTTTGAAGG |
| 1616 | scaffold10302 | CAGAGGTGAAGCAAGGGAAG | AATGGGCAAAGAAGAGGGAT |
| 1617 | scaffold31999 | CAGCTTCTGGTGATGCGTAA | CCAAGAAAACAAACGGCTCT |
| 1618 | scaffold16442 | CAGTGGGATTCCAACGGTAT | TCTTCGAGCCCATCTCAGTT |
| 1619 | scaffold28894 | CATCAACGATGGTGATGCTC | AAACCAAGTACGATCGGCAG |
| 1620 | scaffold4354 | CATCGTGAACGTGCTCATCT | AGGAAGTTCAGACCAAGCGA |
| 1621 | scaffold5967 | CATGGTGAAAAGGAGGAGGA | CCCTGAACAAAAACCAGACAA |
| 1622 | scaffold51649 | CATTCATCTTTTGAAACAATACCG | GGCGGTGGATGTGTCTCTAT |
| 1623 | scaffold7328 | CCAAAGCTGTTGGATCCTGT | GCAACTCCAAGGTATTTGCAT |
| 1624 | scaffold38427 | CCAGTGGGCCTTTCAGTAAA | AGAAGGTGGAGACGACGAGA |
| 1625 | scaffold47134 | CCATTGATTAGCCCCAAGAA | AGCCACCAAGAAAATACCCA |
| 1626 | scaffold17284 | CCCAACAGAAACTCAAGCAA | TTTCGAATTGGGAAGGTTTG |
| 1627 | scaffold39336 | CCCACAATTCGGTATTCACC | GTCTTGCACCACCGAAAGAT |
| 1628 | scaffold15263 | CCCACGAAATGCCACTCTAT | TCATCGACAAAGCTCCACAG |
| 1629 | scaffold18696 | CCCCAGTACCAGTGATAGCG | TTTTAGGATTGGAGGAGGCA |
| 1630 | scaffold1146 | CCCCCAAGTTAAAGGCAAAT | ATCCAGTTCAAGCCTGTTGG |
| 1631 | scaffold871 | CCGCATAGGGGTATTCATTG | GCAGCAAGGAAACAAAAAGC |
| 1632 | scaffold499 | CCGCCGATTCTGATTAGTGT | GGAATAATCGCTTCCGTCAA |
| 1633 | scaffold17929 | CCGCTTACCTTTCGATTTGA | TAAGGTCGGGAAGCAAGTTC |
| 1634 | scaffold33805 | CCTGAAACGAATCGGTGAAT | AGCTCCGACCTTTCTCAACA |
| 1635 | scaffold10213 | CCTGAAGGACGGAGACAGAG | TGCGAAAGAAAGAGAAACCC |
| 1636 | scaffold61309 | CCTTGGTAAGCCAGGCAATA | GATTTTGGGGAGCAAGGAAT |
| 1637 | scaffold14529 | CGAAGACATAGCCTTCTCCG | GCGCAAGAGGCGAGTTATTA |
| 1638 | scaffold21720 | CGAGAAGGAACTCCAACCAA | TAGCTCCACCTCCTCCTTGA |
| 1639 | scaffold5081 | CGAGAATCTCATCTGGCTCC | TGCATGACTAATGCTTCGGA |
| 1640 | scaffold60570 | CGAGCGAGAGAGTGTGACAG | GGATGATTCCGTCTTCCAAA |
| 1641 | scaffold16124 | CGCAACCCTAAGTCCACATT | AGCATCATCGACCCTGAAAC |
| 1642 | scaffold13319 | CGCATTTGGTACGAGGTTTT | CTTCGATCTCAGCCAGCTCT |
| 1643 | scaffold18276 | CGCTGAGCCTCACTCATGTA | GAGATGTTGCTCCTTTGGGA |
| 1644 | scaffold15317 | CGGATCGAAAGGAACAGACT | GCGGCAGAAAAAGGGAGATA |
| 1645 | scaffold13450 | CGTCTCTGAACTCCGTAGGC | AAACAGGCGTGAGCTCTTGT |
| 1646 | scaffold21199 | CGTTAAGGAGAGCAGGCAAC | GTGGAAGGGTAAAACGGTCA |
| 1647 | scaffold8328 | CGTTGAAGCCAAACGAAGAT | CTAACTGCCATCTCGAAGCC |
| 1648 | scaffold40733 | CTCATCGTTTCCACGGAGAT | CTTTCTCGCTCTCCAACCAC |
| 1649 | scaffold25647 | CTCATGATGACTTGGCGTTG | TGGAAAGGGAAGCATCAAAC |
| 1650 | scaffold46827 | CTCCTGGTCGATTTTGGCTA | CACCACTGACCTTGGATGTG |
| 1651 | scaffold2170 | CTGAGGGACTCTGAACCAGC | AAGGCAAAGACTTGACGGAA |
| 1652 | scaffold16872 | CTGATCCCAACCATCCAATC | GCTTCATCATCACGCAGAGA |
| 1653 | scaffold1833 | CTGATTTGGAGGGAAAAGCA | CACTCTTCCGACAACAGCAA |
| 1654 | scaffold36885 | CTGGCCCCTATCATATCGAA | GCCATGTCAAGGCAATTTCT |
| 1655 | scaffold24031 | CTTCGCGGTGAGTCAAAGA | GGTGGAACAAGAACCTTCCA |
| 1656 | scaffold4734 | CTTCGCTCGAATCTCAAATG | GCAACGAAACCCTGACTCTC |
| 1657 | scaffold29612 | CTTCTTAGGTCGTTCCTGCG | AAACCCCACCATAGATTCCC |
| 1658 | scaffold3700 | CTTTCACGCCTCGAGAGTTC | GGACATGAAGCTCTGATCGAA |
| 1659 | scaffold61061 | CTTTGGCAGAATGTGGGAAT | GGTCTTAGCGCAGAGGACAA |
| 1660 | scaffold17456 | GAAAGTGCCTTCCTTGCACT | CCCTTTCACTATTTCCAGCA |
| 1661 | scaffold15279 | GAACAATCGCGAGCCTAAAC | TTGCAAATGCAAACCAAAAA |
| 1662 | scaffold12546 | GAACTTGCAGCAGGACATGA | ACTCAAACAGAACCGAACCG |
| 1663 | scaffold61672 | GAAGGAACAAGCGGACAAAA | TTGCGGACGTCAAAATTGTA |
| 1664 | scaffold15948 | GAATCCCCCATTCATTCCTT | TTTGTTGATGCTCTTGCTCC |
| 1665 | scaffold3974 | GACACACGTCATTCTCCGAA | TTACCTCATCAGGTGGAGGC |
| 1666 | scaffold58441 | GACCGCTCAGCTTTTTCATC | AAATGGGGTTCCACAATTTTT |
| 1667 | scaffold22677 | GACTTCCATCGAGCACCTTT | ACTGGAAACTCTTCAGCGGT |
| 1668 | scaffold10230 | GAGAAGGCATGAAGACGGAC | TTGGGCACGTGGTAGTTGTA |
| 1669 | scaffold29198 | GAGCATCATTAACGCGGTCT | CTTCATTTGCTGGGGATCAT |
| 1670 | scaffold64159 | GAGCTCGGTAGTGGTTCGAG | GGACAGCCCCTTCTCTTTTC |
| 1671 | scaffold11424 | GAGGAAATCACCAGGCAAGA | TGCTCCTCTCTATCATCTCCG |
| 1672 | scaffold14578 | GATTCCCCGCATACAGCTAA | CTAGCATTGTCGACGAGACG |
| 1673 | scaffold11624 | GCAAAATGAGCACCCTTGTT | TCCGGCCATGTAGAAAAGTC |
| 1674 | scaffold5427 | GCAACATACCATTTTTGGGG | ATCTCCTTTCCAGCGAGTCA |
| 1675 | scaffold4078 | GCAACTAGGCAAGATCCGAC | CTCAGAGGTCACGGAGGAAG |
| 1676 | scaffold5141 | GCACTGTCCATCTGCAACAT | CCAGTCTTGGACATGCGTTA |
| 1677 | scaffold6353 | GCAGAAAGGTGGGATTGAAA | TACAACGACCGCGTTTATGA |
| 1678 | scaffold34142 | GCATCCATCTCCTTCCAAAA | TTTTCCGGTGACCTCGTATC |
| 1679 | scaffold33721 | GCATTGACGAGTGCTTCAGA | GGCGCTTCAGACTGGTAAAG |
| 1680 | scaffold16432 | GCCAAAAACATGAGAGCACA | GCGAAGAGCACAGATGATGA |
| 1681 | scaffold61703 | GCCAAACGTTACCCGTTTTA | TCCAAGGAAGTCAATGCAAA |
| 1682 | scaffold11255 | GCCACCAAGACAGAGACGAT | CGATTCACATTCACTCCACCT |
| 1683 | scaffold516 | GCCGGATCAGAATTTTTCAA | GCCTAAACCATGTTTCCACC |
| 1684 | scaffold15174 | GCGAGCGAGGGTGATTATTA | AAACCATGGTCGTCCGTAAG |
| 1685 | scaffold26956 | GCGGGTTACATCCGAAATTA | CTTTTTCAGTCCGTAAGGCG |
| 1686 | scaffold6048 | GCGGTGGAAGAGGAGAACTT | GGGTTAAGGAGAGGGCATGT |
| 1687 | scaffold1891 | GGAAACGTTGAGATGGGAAA | CTGATCATCTCAGGCCACAA |
| 1688 | scaffold51904 | GGAGTTGCGTTCCCATAGAA | TGAAGGGGATGTGTCAAAGG |
| 1689 | scaffold993 | GGATAATGTTGCCAAGTGCC | TTGCAATTATGAAGCGATGA |
| 1690 | scaffold16559 | GGATTTGTTATCCCGCAATG | GTACCAAAGCATATCGCCGT |
| 1691 | scaffold15947 | GGCGATAAACCGAACAAAAA | AGGTCCTCCTCAACACGATG |
| 1692 | scaffold40732 | GGGCCCAAAATTATGAGGTT | AACTGCGTCTGGATCCATCT |
| 1693 | scaffold36014 | GGGGTTTGCAGCAATGTAGT | CGTCGTCCATGTCTAAGCCT |
| 1694 | scaffold53883 | GGTGGACGAAGAAGAAGCAG | TTCAAAAGGGGTTGCTTTCA |
| 1695 | scaffold25358 | GGTGGTAGCTCCGATGAAGA | CTCGTTAAAACTACGGCCCA |
| 1696 | scaffold61797 | GGTGTCAAACCACTGGTGAA | GACCGACCAGACTTCTCTCCT |
| 1697 | scaffold39030 | GGTTATTTCCACCATGCCAC | CTTGCCGAGACTCATCATCA |
| 1698 | scaffold4168 | GGTTTCTTGAGAATGCTCGG | TTGGCAAATAGATGCTTTGAA |
| 1699 | scaffold5556 | GTACGGAGGTTGATCGGAGA | GCAAACTCCGGATCTAGCAG |
| 1700 | scaffold34584 | GTCAGCAAATGGGCAACTCT | CCAGGAGTGTTGTTTGAGCA |
| 1701 | scaffold8229 | GTCTCTCCGCATCTCTTTGG | CCATTCCAGCATCCTCCTTA |
| 1702 | scaffold64122 | GTCTTTCGCTGAAGCCAAGT | GCCAGATTTGATTTACGGGA |
| 1703 | scaffold40130 | GTGATCCGTCCGTCTGTTTT | GGATCGAAACCTGTTGGAGA |
| 1704 | scaffold6793 | GTGATGAGAAGCTAAGGGCG | TTTTGGGAAGTTCCTCTGGA |
| 1705 | scaffold10457 | GTGCCTGTCAAAGCATGTTG | GCTTTCCCACCTGTGTTTGT |
| 1706 | scaffold61309 | GTGTCAAGTCGATGTGGAGC | TTTTTGTCCTTTTGATCGGA |
| 1707 | scaffold8217 | GTTAACCTGCGGGATTTTGA | CCCTGAAACCACTGTCACCT |
| 1708 | scaffold9969 | GTTGCAGCTTCAGCCATTTT | GAGCGCAATTCTCTGAAACC |
| 1709 | scaffold15667 | TAAAATCTTGGATCGGGGTG | CAATTGAGTGGCCTTTTGGT |
| 1710 | scaffold5716 | TAAAGCTTAGAATCGCCGGA | ATTTCCGACGAACACAGTCC |
| 1711 | scaffold15947 | TAACCCTCGGGTCACAATTT | ATCTTGTCTTCATCGCACCC |
| 1712 | scaffold6145 | TAGAACGCAAACCAGTTCCC | CAGAGGCATTGGATGTGTTG |
| 1713 | scaffold29229 | TAGCTTTGCTTTTCGCCATT | TACCCTTTACCCGTCCATCA |
| 1714 | scaffold9074 | TCAACGGCTGTTTTCTCCTT | TCCGTCACTACATCCCATTTT |
| 1715 | scaffold10324 | TCAAGGTTTCTTGCAAATCTCA | TGCGGAACATACAACGAAGA |
| 1716 | scaffold10557 | TCACCCTATGCACCGTTAGA | AACTCAAGGCCGACAAAGAA |
| 1717 | scaffold6710 | TCACCCTGTGAAAAATGCAA | GGAAACAGCCACTTCACCAT |
| 1718 | scaffold18860 | TCACGCAAACAACCAAATGT | ATTGTCACAAACCCCACGTT |
| 1719 | scaffold7399 | TCATCGACAGGAAATCATGG | GCCCCTTCATCATTCTTCAA |
| 1720 | scaffold3294 | TCCACGAGACTCAACGACTG | TCCATTTTAGAGAGAGTGGGAGA |
| 1721 | scaffold3359 | TCCAGTTCCAGTAGCTTGTTTTC | GGTTTACCGAGTCAACCGAG |
| 1722 | scaffold29385 | TCGAGCTTTCAGCCTTTGAT | ACACGAAAGAAAGCGGAGAA |
| 1723 | scaffold7283 | TCGATGCAAGACCCTAAACC | CAAAACCAAAAGCAAAACCA |
| 1724 | scaffold26977 | TCGATTTACGTCTCTCACGG | AGGAATGTGGTTGTGGCATT |
| 1725 | scaffold1928 | TCGCCATCAAAATCAATCAA | CATTCAGTTTCCTTCTCCGC |
| 1726 | scaffold3359 | TCGGCTGAACTCAACAACAG | TGTGAAGCGTGCTGTTTCTC |
| 1727 | scaffold7383 | TCGGTAAACCAATCCCAAAA | GAAGGAGCTGAGCAAGGAGA |
| 1728 | scaffold1745 | TCGGTATACCAAGACCCTGC | CAGCAATAAAAACGGCACAA |
| 1729 | scaffold1745 | TCGTGGGTAAGGTCTCCATC | GGTCTCGATTTAAATGCCCA |
| 1730 | scaffold51371 | TCTACCATCAGTCTGCAGCG | TGCCGACAGGTTATCTCTCC |
| 1731 | scaffold51506 | TCTCCACCACTTCCCTATGC | ACGAGAATGTCCTTGTTCCG |
| 1732 | scaffold20576 | TCTCGTTCCTGAGACTCGCT | GATCGGTGGTGGAAGAAAGA |
| 1733 | scaffold10487 | TCTGAGGAATCGAAATTGGG | GCGTACACTTGTCCGGTTGT |
| 1734 | scaffold16432 | TCTGTGCACCCATTGATGAT | AACGGTGAATGCTTCTTGCT |
| 1735 | scaffold58676 | TGATGGTGAAAACAGCCAAA | CATGGAGAGAAAAAGACCCG |
| 1736 | scaffold17086 | TGATGGTGATGATGCCAGTT | TTCTCTGCCTCCGAGTCTTC |
| 1737 | scaffold17525 | TGCATGAATGCTTCGAAATG | CCCTAAAGCTTGCAGTGGTC |
| 1738 | scaffold32469 | TGCGAGATACGAGGAAGACA | GACCGCCGAAAACATTAAAA |
| 1739 | scaffold13535 | TGCTCCAAAAATAAGCTCAGG | ATTCACCGTTGTATTGGCGT |
| 1740 | scaffold51299 | TGCTGGAGACACACTTTTCG | CCACCACCTCCTCCTATTGA |
| 1741 | scaffold3528 | TGGAGAGCAAGTTGAGGACA | CACCCAAACACACACAAAGC |
| 1742 | scaffold16995 | TGGCTTGCATTCAGCTAAGA | CCTTCCTGTTTCAATCCTCG |
| 1743 | scaffold1025 | TGGGATCCATTTTGTTAGCC | TCATTGGTTTATGATTTGTAGAGCA |
| 1744 | scaffold21329 | TGGGGTACTTGGAACTAGCAA | GTATATGGAGGTGGCAGCGT |
| 1745 | scaffold6301 | TGTCCACAACGCTTTGTTTC | GGTCCAATGGGAGAACCTCT |
| 1746 | scaffold3074 | TTAATGTGGTGGCGAATGAG | CCACCGACACTGACCTTTGT |
| 1747 | scaffold58684 | TTCAAGACCGAAGAGCACAA | CGCTTGAACAAACGCAGTAA |
| 1748 | scaffold20669 | TTCAGCACCGACTGACTCAC | TCAGCGTCAAAGGTTTTGTCT |
| 1749 | scaffold20679 | TTCATCACCTCCACTCCACA | AAGGGTACGAAATGATGCGA |
| 1750 | scaffold46873 | TTCATCTTCAGCTCCAACCTT | GGACCAGTTTCCAGCCAGTA |
| 1751 | scaffold15313 | TTCCACCCGATGAGAAGAAC | GCTTAAGCTGAGAAGCCCCT |
| 1752 | scaffold3205 | TTCCGGCCCAGTAACTTTTA | AAAATGCACGTACCGTCACA |
| 1753 | scaffold51444 | TTCCTCCGATCGAGAATCAC | TTTTCTTTTAATTTGCGCCG |
| 1754 | scaffold32377 | TTCTCTTTTCCTTTTGGGCA | CACCCAAAAACAAGAACCCA |
| 1755 | scaffold36599 | TTGATCTCCCTCAGGTTTGG | CGAATCCATGTTCCCTTTGT |
| 1756 | scaffold7520 | TTGCTTTTGCTTCTGGGTTT | TGTTTGTGTCCGAAATCACG |
| 1757 | scaffold25261 | TTGGATATTCTTCGGTTCGG | CCAAGTTGGAAAGAGCGAAA |
| 1758 | scaffold15107 | TTGGGAGAGCGAGAGTGAAT | TGGCTGATTATTGACGAATTG |
| 1759 | scaffold2515 | TTGTTTTTCTTCGCTTCTTTTT | CGGAGAAGGAGTGGAGTGAG |
| 1760 | scaffold18169 | TTTCAAGCGAGGAGCTGATT | AAGATGCCCTTATGTGTCCG |
| 1761 | scaffold15949 | TTTCATGTCGGAGACTTCCC | CCAAATCGGATCTTTGAGGA |
| 1762 | scaffold58283 | TTTCCAGGGATGGTTATGGA | CCGGCTCTGATACGCATACT |
| 1763 | scaffold8545 | TTTTAGACGCAACGAGCAAA | GCGTTTGTCCAACGAATTTT |
| 1764 | scaffold11099 | TTTTGCAGCTTCCACCTTTT | CAAAGCTCGTTGGAGAAAGC |
| 1765 | scaffold62461 | AAAAAGGGAAGTGTGAGGGG | TGAATGTAAATGGATCGGCA |
| 1766 | scaffold14302 | AAACGCGGATCAAAGAGGAT | AGCTCTTCGACGGATTCAAA |
| 1767 | scaffold28166 | AAGCTTCTTCAGCCTCCTCC | CTAAGAAGGAACCGGGAAGG |
| 1768 | scaffold43843 | AAGCTTTCCAGCAAGGTCAA | AAGAGCGCTTCACTTTCGAG |
| 1769 | scaffold119 | ACATGGCCCCTCTAAAGGTC | ACTGGAAGTAAATGGTCGCA |
| 1770 | scaffold17311 | ACCAGACCTAAAAACGCAGC | CCGTTGAAGAGAAGAGACGG |
| 1771 | scaffold14637 | ACCAGGAGGACGCATGTAAC | CAAGTGAGAAGCGCAATCAA |
| 1772 | scaffold7400 | ACCCAACAAATGCTGGAGAG | CCGTTCTACCAATTCTGAGC |
| 1773 | scaffold9711 | ACCGCAAAACCAAACATAGC | GTTTGCATTTGAGGGCAGTT |
| 1774 | scaffold16916 | ACCGTCAATCAGAAACGGTC | CTAATTAACCACGGAGCCGA |
| 1775 | scaffold867 | ACGAAGATTTCCGCAACAAT | ATTTTCGAGACCCTGCTTCA |
| 1776 | scaffold1780 | AGCCTGTTCACGAGAAGCTC | TGCTGCCTGAAAGACTCAAA |
| 1777 | scaffold22889 | AGCTCGTCGGAGTCTGATGT | TCATTCATCTTTCTTCCCCG |
| 1778 | scaffold18469 | AGCTGCAATGCAACATCAAC | CTTCTACCTGCAAAGGCGAG |
| 1779 | scaffold23614 | AGCTTCTCGCCCTGACAATA | ACCGGCATCAAATCTCAATC |
| 1780 | scaffold12474 | AGCTTGAGAACCCTCCAACA | AGACTCGCTCGGTGATGATT |
| 1781 | scaffold39221 | AGGCCCTCTCTGTCCATTCT | TAACCACATGGCCTCCTCTC |
| 1782 | scaffold41199 | AGGCGAATCTGAGTAGCAGC | CTCCGCCATGAGAGAAGAAG |
| 1783 | scaffold60868 | AGGTTTCTTCGAGGTGGGTT | TCATAAAGATTCCTCGCGCT |
| 1784 | scaffold1653 | ATCATGGTTCCCCAAGACTG | TACAGATGGGAAGGGTTGGA |
| 1785 | scaffold10188 | ATGGAAACAGAGCCAATGCT | AAATTGAGTCGGTGGTGGAG |
| 1786 | scaffold5751 | ATGGCCATCGTACTTTGAGG | CAAAGCCACGATTGCATTTA |
| 1787 | scaffold1702 | ATTTGGTATTGCGACTTCGG | GGGTGCGTTTGTAAAGTCGT |
| 1788 | scaffold18593 | CAAACACCAAGAAAGCGACA | AAGCAATGATTTGTTTCCGTTT |
| 1789 | scaffold1930 | CAATCAACTTCGGATCGCTT | AGACAGGTTGCTTCCAGCAT |
| 1790 | scaffold49756 | CACAAATCGCATAACCTCCA | CAAGTTGGCCCTTGGAAATA |
| 1791 | scaffold12202 | CACACCAAGGACACTCCAAA | AAATCCCAAAACCCAACCTC |
| 1792 | scaffold50127 | CACCTCCCAAGCTCAGTAGC | TCGTCACATCTGCCCATAAA |
| 1793 | scaffold8422 | CAGAAACGCCCTTCAGTTTC | AAGAGGGCTTCCCTTCAGAG |
| 1794 | scaffold2781 | CAGAATACCCCTGCACGACT | CATATCGGCTTCTCCAATCC |
| 1795 | scaffold62369 | CAGGATTTCGGAGAGAACCA | TGGCCGAGTGCTATCTCTTT |
| 1796 | scaffold47877 | CCATTCGTGAGCTTTCCACT | TGGGAAATCCAGATTCAACC |
| 1797 | scaffold5040 | CCCTAAACCGATGCACAACT | GGGATTAGCATTGACCCTGA |
| 1798 | scaffold23251 | CCGATCTCAAAGTTCGGATT | TTGGTTTCTGGTTTTCCCCT |
| 1799 | scaffold6142 | CCGGAGATGATCTTTGGTTT | TGAGATGTAGCACAAGTCCTCAA |
| 1800 | scaffold997 | CCTTTTAGCAATGGGGAAGC | GTGCCGTTGGTAAAACCTGT |
| 1801 | scaffold24978 | CGAGTCTTTGGTTCCTCGAC | AGGTGAAACTGGGCTAGGCT |
| 1802 | scaffold2220 | CGCATATCCTTCTCAGGCTC | ACGACATTGCCCCACATAAT |
| 1803 | scaffold32962 | CGGTTTCGTCTTCACTGTCA | AACAACCTCAACTTCCACCG |
| 1804 | scaffold3607 | CGTAGCCGTCTCTTACGCAT | TTCAGTCCAGCGTTCAACAG |
| 1805 | scaffold35400 | CGTCGATTTTGCTCTTCCTC | TAGTCGCAGTGGTCAAGACG |
| 1806 | scaffold16091 | CGTGGGCCTATGTTTTCTTC | TCTTCTCTCTCCCAAAAGCA |
| 1807 | scaffold27943 | CGTTGTCTCAAATCAATGGC | TCATCCATTATCATCGGGGT |
| 1808 | scaffold799 | CTAACACACCCGTAGCGTGA | TGATTCATCTGCATTTGCGT |
| 1809 | scaffold6839 | CTACCTTTGGATCGCAGGAG | ACATCGGTTTGATGAAAGGC |
| 1810 | scaffold47425 | CTCATGTTGCGCCTACAGAA | CAGAGAATTGGGTTTGGCTC |
| 1811 | scaffold8640 | CTGAGCTCAAAACAGCACCA | AGATTAGCCTTCCCTCGCTC |
| 1812 | scaffold47235 | CTGCTGTGTCCGTCATTCAT | TCCAATCGAACTGGGGATAG |
| 1813 | scaffold10556 | CTGGCTCTGTCTCTTGAGGG | CCAACCACAAAGGAGGAAGA |
| 1814 | scaffold51081 | CTTCTCCAACAAAAGCTCGG | AGAGGACTGGTCCCCAAAGT |
| 1815 | scaffold9866 | GAGAGAAGTGAATCTGGCGG | TTCCGCGTTTTAACCATCTC |
| 1816 | scaffold15046 | GATCGAAGCGAACCTGTGAT | TGTCCATGAGCTGTTTCGTG |
| 1817 | scaffold6624 | GCAAAAACCACCTTCCTCAA | AGAAAAACTGGGGCGAACTT |
| 1818 | scaffold15283 | GCACGAAGACGCACTAAACA | TGAGCTTCATTCACGTCCAG |
| 1819 | scaffold3847 | GCATTTCGTTGGGTGTCTTT | TCTCCCTAATATCGATGCGG |
| 1820 | scaffold12255 | GCCATCATTGATCCCAGTTT | TCGAATTAAACCGAACCGAG |
| 1821 | scaffold50798 | GCGAGAGAGAGACATCCCAC | CATGTCCCTGAACCTTCCAT |
| 1822 | scaffold25649 | GCGTCAAATCATTCCCATCT | TGGTGAAAGTCATCCCATTG |
| 1823 | scaffold580 | GCGTTCGGATGAAGAAGAAG | TTTACCCACACAGGCTCACA |
| 1824 | scaffold1216 | GCTCTGTGACTGATCTGAGCC | CTTTGACCGCCATTGATTCT |
| 1825 | scaffold16292 | GCTGATCCGGGTATTAACGA | CCTCTTACAAGCTTCCACCG |
| 1826 | scaffold16898 | GCTGTAAATCCCCAACCTGA | TAAAAGAAGGGGATTTGGGG |
| 1827 | scaffold20031 | GCTTGAGGTAAGCTCATCGG | CGTCCATTGGGCTGTTAGTT |
| 1828 | scaffold6970 | GGAACTCAACGAGGAAAACG | AGAAGCTTTGCTTTGCTTGC |
| 1829 | scaffold632 | GGAGGGCGAAGAGAGAGACT | GTTTTGACGCAACGGATTCT |
| 1830 | scaffold39269 | GGATAAGACCAAGTTCGCCA | CTTTTGGCGCACACACATAC |
| 1831 | scaffold6125 | GGATCTAGGACACAAGCAGCA | TGAATCCTCCTACACCGTCC |
| 1832 | scaffold7435 | GGCAAGAGCTTGGGACACTA | CCTTCAAAAGAAAGGAGGGG |
| 1833 | scaffold24445 | GGGAATCGGACAAGAGATCA | ATTTCGATTGCCAAAACCTG |
| 1834 | scaffold10223 | GGGGAGATACGGTGTCTTGA | TCGGTTTAGCTTCAGTTCCG |
| 1835 | scaffold24821 | GGGTGGAACTTCAACTCCAG | ACATTTCCACGTCAACCCAT |
| 1836 | scaffold13472 | GGTTACGGAATCGAAAGCAA | ATTAGTCACAAAGCCCACCG |
| 1837 | scaffold970 | GGTTTCTCAGTTCGACGGAA | GCGCAAAAGCCATATAATCC |
| 1838 | scaffold22715 | GTTCCAACTGGTTGGTTGCT | ACAGAAACGCTTGCCTTCAT |
| 1839 | scaffold395 | GTTGAGTAGGGTTCCAGGCA | CATGGTGAAAAACATCCCAA |
| 1840 | scaffold7521 | GTTGCGATACGGAAGGAAAA | CAGAGTCCGTTCCCCTATGA |
| 1841 | scaffold941 | TAGGGGGAGATTGGGTCTTT | GTCATCGGAGCTCCATTTGT |
| 1842 | scaffold17169 | TCAAACGTTTTGCTGTTTGC | AGATTGAAGCTATGCGGAGG |
| 1843 | scaffold17217 | TCACACCACCTGAACCAAAA | GGTGGGTTTTGAAGCGAGTA |
| 1844 | scaffold17651 | TCAGATCCTTCAGAGCAGCA | GGAAAACCTGCACAAGAGGA |
| 1845 | scaffold18491 | TCATGGAGAAAATGGGGGTA | ATTATTCTTGGATCGGTGCG |
| 1846 | scaffold4225 | TCCAACTTTGTTTTCTCCACC | TGCACGAGATTCTCAATGGT |
| 1847 | scaffold4037 | TCGAGGGATGGATGAGAAAG | CAAATCCAACGTCTCCGTTT |
| 1848 | scaffold5088 | TCGGGGAAATTCAGAAACAA | AACGAAAGGGGGTAGTTTGG |
| 1849 | scaffold56236 | TCGTCAAATTAGTATTGGTTGGAA | AGGAAAACATATCCTCCGGG |
| 1850 | scaffold43442 | TCGTCGTCATTTCTCTGTCG | CTGTAACGAAAGGCGGGTAA |
| 1851 | scaffold12466 | TCTCTTTTTCGCCGTGATCT | CCTTGGAGCTAAAGTCCAACAC |
| 1852 | scaffold15746 | TCTTGATTAACACGCAGCAAA | TGCTCGAGTAAACCGGAGTT |
| 1853 | scaffold17969 | TGACCCAGCTATCGCACATA | GGTTCTTCTTGCCGTCGTAG |
| 1854 | scaffold16691 | TGCAGAGGAAGTTAGGTGGG | ATGGAACCAAAAAGCGAAAA |
| 1855 | scaffold12485 | TGCATGGCAAGAACAAGAAG | TGTCTGTTGCTTTTGATCGC |
| 1856 | scaffold50383 | TGCCACAGGTTTTCTTGTTTC | GCACCTAAAGCAAGTGCATGT |
| 1857 | scaffold8371 | TGCCTCATAGTTCAGGTCCC | CAGGAAACCTCACGGATGAT |
| 1858 | scaffold14321 | TGCCTCTTTCTTCCCTTGAA | AAAGGATATTGCTGCAACCG |
| 1859 | scaffold595 | TGGGCACCATTCCATATTTT | TCTCAACTCCTTTTTCCCTCA |
| 1860 | scaffold583 | TGTCTGCGTGGACTTGTTGT | ATCTGCGCTAAAGAAGGCAA |
| 1861 | scaffold17302 | TGTGATTCAAGAACAGCCCA | CACACGCACACCTACGACTT |
| 1862 | C18314727 | TTAGGGCTTAACTGCATGGG | TCTCTCTCTCTCTCTCTCTCTTCTTC |
| 1863 | scaffold18089 | TTATCGACGCCGTCTTAACC | TTGCTTTCAATAGGGGATGG |
| 1864 | scaffold29893 | TTCACAGATGATTGCAGGAGA | TTCGAAACCAACCCTTCATC |
| 1865 | scaffold6536 | TTCTGGTTCTCGCCATTTTT | AAACCAAACACCCCAACTCC |
| 1866 | scaffold10538 | TTGCTTCCTCCATCCCTAAA | CAAAATCTTTTTAAATTCTTTGGATCT |
| 1867 | scaffold44818 | TTTACTTGACGAAATCCGCC | CGCTTCCTCCCCTTTACTCT |
| 1868 | scaffold13220 | TTTGAGGCGTTGGGTTTAAG | ATGGGCCATATGCTTCAGTC |
| 1869 | scaffold30856 | TTTGCGGAAGTGTTTGAGGT | GCTGTCCCTTTCAGCTCATC |
| 1870 | scaffold22638 | TTTGTCAACAGACAGACGCA | TCGATTAAGGATCGGAAACG |
| 1871 | scaffold14586 | TTTTGGCGATTTTTGAAAGG | GGAAGGGGCCAATAGTTGTT |
| 1872 | scaffold14195 | AAAGGTTAGGTGGTTGGATAAAGA | TGTCTTCTGATGCCTTGGTCT |
| 1873 | scaffold5530 | AAATAGGCGGAGGAGGAGAA | TTTTGCTTTAGGTTGAGAAACG |
| 1874 | scaffold18089 | AACTGCTATCCATTCCCCCT | GGACCAATCGAGACGTTGTT |
| 1875 | scaffold24011 | AATACGTGTGTGCCACCAAA | CCTTGTACGCAAGATGAATAAA |
| 1876 | scaffold13578 | AATCACCGGCGTTGTTTTT | TGCTCTTAGGACCATCTGGG |
| 1877 | scaffold21183 | AATCTCTGAGACCAATGCCG | CAAAGTCTGCCTGCCTTCTC |
| 1878 | scaffold38115 | ACAGCCTGACCGTAACAACC | TGCGTGGAATTGATCTGAAA |
| 1879 | scaffold60757 | ACCAGAGTAAAGCGAAGCCA | TTCTTGATCAGCTCCCTCGT |
| 1880 | scaffold17938 | ACGGTGTCGCTGCTAAGTCT | CACTCGATCCTCCTCCACAT |
| 1881 | scaffold997 | ACTCACATCAGTGGCAGCAG | ATTTTCGCACCTCGAGTTTT |
| 1882 | scaffold1163 | ACTCCAAAGCCATTTCAAGC | AGGCGGATCTTAACGGTTTT |
| 1883 | scaffold35418 | AGAAATCATCCAACCAAGCG | TCGAAGTTGGGTTGAAGAGC |
| 1884 | scaffold23350 | AGAGAGGAAGGGTCTAGGCG | TGCCATTTCCCAATTCTCTC |
| 1885 | scaffold47341 | AGAGGAGGGAGATGGCAGAC | AAACACCATTTCATCTCCCG |
| 1886 | scaffold58610 | AGCAAAGCCATGAATCAGGT | CCGTTCCATCTGACGAGTTT |
| 1887 | scaffold54466 | AGGCTGAGGAGGGAGAAAAG | CGCAACTCGAATCAGAAACA |
| 1888 | scaffold27099 | ATCTGAACATGGGGGATTGA | TGACAACAAATCTTTTCAAGAGTCA |
| 1889 | scaffold33264 | ATGTCGGGGTCATCACACTT | CCTCGAAAGCCACGTACACT |
| 1890 | scaffold23252 | ATTCACCAAACGCCATCTTC | TCCTGTCCATTCTCTTTCGG |
| 1891 | scaffold56604 | ATTCCACAGAGACCTGGCTG | ATTCCACCTGAAACCCAACA |
| 1892 | scaffold43814 | ATTTAGCTGCAAGCCACAGC | TGGAGGACAGAGAGAGCACA |
| 1893 | scaffold13564 | CAAAGAACGAACAAGGGGAA | AGTTGCAGCCTTTATCCGAA |
| 1894 | scaffold6851 | CAACTGGTGGGTCCTTTGAT | CAACACACGCATACACACACA |
| 1895 | scaffold2553 | CACCACTCCACCACAAATGA | AACATTGTTTGGTCCGAAGC |
| 1896 | scaffold3783 | CACGGTCCAAAAGATCACCT | CAAGTTCCATTTCCCTGGTG |
| 1897 | scaffold2957 | CACGTAGACAAGAAGCGCAG | TTTGTTCATTTACTCCCGGC |
| 1898 | scaffold10837 | CAGATCTGCTTATTCCCCCA | CACTTACCACGTTCCACACG |
| 1899 | scaffold2517 | CAGTGAAGCAACAAGCCAAA | TCATCTTCAATCTTCATCGTGG |
| 1900 | scaffold61643 | CATGGAAGGATAGCTAGCGG | CGTGTCACTTTATCGACCCC |
| 1901 | scaffold18450 | CCAAGTCTGCTCCTTTCCAG | GATGAAGAAGATAAACAACAAGTTCC |
| 1902 | scaffold658 | CCACAAGAGCTGATGCGATA | GTCACCAGTCGGACCTTTTT |
| 1903 | scaffold7199 | CCACCAAACTCCATCGTTTT | TAAAACCCTGCGCCTAGAGA |
| 1904 | scaffold23165 | CCATCGATTCCGTCAAAACT | GCTTTGAGTGTGGTTTGGGT |
| 1905 | scaffold10869 | CCATTCTCAGGAAGTGCTCC | CCTCTCAGTCCCCAATTTGA |
| 1906 | scaffold2861 | CCCAAATCATCCTCTCCTCA | TAAATCAAAACACCCTCCGC |
| 1907 | scaffold23742 | CCCATTTCTCAATTCCCAAG | GATTGCTTCTTCCCTGAAACA |
| 1908 | scaffold37804 | CCGACACTAACCCGCTACAT | GAAGTTGCGTGTCGTCTTCA |
| 1909 | scaffold28303 | CCGACAGCAAACAAGATCAA | TCAGACCATCTGTTTGGTCG |
| 1910 | scaffold15976 | CCGAGTTAATCGAACCAGGA | TCGCCACCATCTACCTTACC |
| 1911 | scaffold2241 | CCGCTTTTCTATTCCTTCCA | ACGGATCTCCACATTGCTTC |
| 1912 | scaffold29125 | CCTGACGGTCCTCTTTGTGT | GTCAAACGTGCCAAAAAGGT |
| 1913 | scaffold20113 | CGAAACAATGATGAGAGCGA | TGCTAAAGAGAAACCCACCAA |
| 1914 | C18466649 | CGAAGACCTCTGCATGTTCA | AACAACTATGGGTGCAATGAAA |
| 1915 | scaffold51713 | CGAAGGTTTGATGAGTGGGT | TCTTTCCTCCCAAACAAACG |
| 1916 | scaffold40892 | CGATTGCTTTCTCTGCAACA | CACACTTTCGCAGAGTCCAA |
| 1917 | scaffold13364 | CGATTGTTGGGTCTCAAGGT | CGCAAAGGCAAGATTTTGAT |
| 1918 | scaffold7415 | CGGAATGAACCAAAAAGGAA | CAAATCCAGAGCGACGTACA |
| 1919 | scaffold31932 | CGGATAGGGTGAATCGAGAA | CTCCCTTCTTCCTGCTTCCT |
| 1920 | scaffold1118 | CGGATTGATCATCACCACAG | CTCATTTCACGCCTTCTTCC |
| 1921 | scaffold920 | CGTAGACGATGAAACGCAGA | TCCAATTGATTTGTGCGAGA |
| 1922 | scaffold61352 | CGTGAACCTTCATGGTGATG | GGAGAATGGTCGCGAGATAA |
| 1923 | scaffold2864 | CGTGCGGAGTATTTCCATTT | CAGAGCTCGCTTCTGGACTT |
| 1924 | scaffold30854 | CTCCGACCATCTTTCTGCAT | GGAAGGAGATGAAACTGCGT |
| 1925 | scaffold21631 | CTCCGGTCATTGATATTGGC | GCCTTTTTGGTGCATGTTTT |
| 1926 | scaffold21727 | CTCTTTCAAGATCCGCTTCG | AGCTGGAAAATAGCTGGCAA |
| 1927 | scaffold10837 | CTGTTCCTCGACTCCGTCTC | AGGAGGAACAAACGGAACCT |
| 1928 | scaffold32219 | CTTCTTCCTCGCAGTTACCG | CGGAAAATCTGGATATTGGC |
| 1929 | scaffold26500 | CTTGGGAAGCAAGGAAAACA | TCTGCTCGTGCCCTAGATTT |
| 1930 | scaffold25831 | GAAAACCCTGAGTCAATCGC | ACCATATCATCTTACCGGCG |
| 1931 | scaffold20240 | GAAACCTTGAACGCATGGAT | TGCCTCCATTACCATCAACA |
| 1932 | scaffold22567 | GAATGCAATCGAGGGCTAAG | CAAACTATCCCCGGATCTGA |
| 1933 | scaffold38856 | GAGAAGCAATCACTGGTCCC | AAACGCATGTTTTGTCGTGA |
| 1934 | scaffold35754 | GAGGAAATGAGATCCATAGGTCA | GGAATGGAAATATTCTGAAACTCC |
| 1935 | scaffold42607 | GAGGCTGGCTTTGTATGGAG | TCAAAACGATCCCTCAGGTC |
| 1936 | scaffold3162 | GATATTACTGGCCGGGGATT | TTTTCGGTGGAGTATCGGTC |
| 1937 | scaffold4026 | GATCGGTTAGATGGGAAGCA | TCGATTTCCGTTCAAATTAGG |
| 1938 | scaffold45569 | GATTGCTCACAAGGGTCGTT | CTCTTCGCAGTGTTCTTCCC |
| 1939 | scaffold39144 | GCAGGGGAGACTTGTGTCAT | TATGCTTTGCTGTGCCTGAG |
| 1940 | scaffold34495 | GCAGGTGAGGATCGGAATAA | ATTCCCGAGGAGAACTGTCA |
| 1941 | scaffold54282 | GCCAAGACTTCACGGTCATT | TGTCTGTCTGGTCCCTCTTT |
| 1942 | scaffold16910 | GCCGCAAATTACTCAATGGT | CATGATTATGCGCTTGGATG |
| 1943 | scaffold52724 | GCCGCTTAAAACGAATATGTG | GGTAACTAATCGAACCGCCA |
| 1944 | scaffold7640 | GCGAGGAAAACGTTGAGAAG | TCAAACCAATCTCCACCCTC |
| 1945 | scaffold61601 | GCTCCTCTTCATCGTTTTGC | AGACTGGAATCCGGGAAACT |
| 1946 | scaffold52892 | GGAGATGATGGTTTCTCGGA | ACTCGGACCTGTTTGGTGAC |
| 1947 | scaffold741 | GGAGCCCTAGGTCTAATGGG | CTCAGGATCCTTGGTCGAGA |
| 1948 | scaffold23828 | GGATCCCGTTTCTGAACTGA | TACATCGGCGGTTCACAGTA |
| 1949 | scaffold1891 | GGATTGGGGTGAGAAAGGTT | ATATGCCGCAACACCTGAAT |
| 1950 | scaffold16602 | GGCAGGCCAAATTTACGATA | GGTGACACCACTCAATGTCG |
| 1951 | scaffold36571 | GGCGAAAACAACAACCAAGT | TGGAAATTATCGAAGCTGGG |
| 1952 | scaffold38895 | GGGAGGTGGCGAATGTATCT | AAAAATCCACCAACGCTGAG |
| 1953 | scaffold698 | GGGCTTATCTCCACCTCACA | GAAGAAGCGGAGACTTGGTG |
| 1954 | scaffold27971 | GGGGACGAAGAAGAGGAAAG | TTAACCTCTCGTGCGGATTC |
| 1955 | scaffold36624 | GGGTGTTGCTCCTTTGTCAT | GTTCAAGCCATCCTCTCTGC |
| 1956 | scaffold57319 | GGTCCGAGTTCCAACACAGT | CAAGGGGGTTTTGATTCCTT |
| 1957 | scaffold36953 | GGTTTAATCTTTTAAGCCATAGCC | CAAGCAAAAGATGTGTCTGGA |
| 1958 | scaffold8150 | GTAAGTTGCGCAAGTGCAGA | TTGGAGAGGCGAGATTTTTG |
| 1959 | scaffold8161 | GTAGGTCGTCGAGCTTGGAG | TCTTACTATCCCCATCTTTACCTCA |
| 1960 | scaffold37347 | GTCACACGAGGCTTTCCATT | GTCTTTTCATTCTCGTCCGC |
| 1961 | scaffold10397 | GTCATACGCGAACAACAACG | CTGGGGAAAAAGAAAGCCAT |
| 1962 | scaffold26945 | GTCCGGACTCTTCGATCTCA | TCAAGACCGAACCAGCTTCT |
| 1963 | scaffold19424 | GTTAACCTGCGGGATTTTGA | AGCATCATCGACCCTGAAAC |
| 1964 | scaffold16077 | GTTTCCAAATACGGCAAGGA | CAGTAGAAGCAGGGTCCCAA |
| 1965 | scaffold63704 | GTTTGGATCGGATTTGGAGA | CCCCACTTTTCCTCATCTCA |
| 1966 | scaffold5520 | TACAAAAATGAAGGGCGAGC | GAAAGCAAGGCATTCACACA |
| 1967 | scaffold20009 | TACGGAATGTGTCAACGCTC | TGGAATCATAATGGGAGGGA |
| 1968 | scaffold13047 | TATCCCAGGCTGATTTCGAC | TCGGTTCAGATCTTGAAAAGAAT |
| 1969 | scaffold12913 | TCAAAATCGACCGTAAAGGG | ACAAATTACCCCACACGGAA |
| 1970 | scaffold37378 | TCAAACCAACCTTCCCTCAC | CCTCTTCTTCTTGCCCACAG |
| 1971 | scaffold6922 | TCCAGAGCAGCGTATAGCAA | CCAAAAACACGGAGAAAGGA |
| 1972 | scaffold5233 | TCGAAGATCCAACAACACGA | TCATCGGCTCTGTTTCTAAGG |
| 1973 | scaffold2874 | TCGATTTGAACGGAATGGAT | GGCCTAATGGGCTTCACATA |
| 1974 | scaffold26869 | TCGCCATGTCTCTCTCTTCA | GTCCCATGTTGTCGGAGATT |
| 1975 | scaffold21432 | TCGTTGCTTCCTGATGTGTC | CGGAAACGGATAACCTTTGA |
| 1976 | scaffold13234 | TCTCAAACGCTTCTTCTCTGG | TCGTTGGGTCTTTGCTTGAT |
| 1977 | scaffold29795 | TCTGACCGGAGAGAAATTGG | TGGTTTAACGAAACCAAGGC |
| 1978 | scaffold25806 | TCTGCAAGAGCTTGTCCCTT | TGGATCCGTAACATTGAGCA |
| 1979 | scaffold24558 | TCTGCGACGTACAGACATCC | AGATTCATGGCGTTTCTTGG |
| 1980 | scaffold13994 | TGAAACAAACAAAGCCTGTGA | TTGTTTTCCAACTCTTGGGG |
| 1981 | scaffold27784 | TGACGATTTTCACGGAACAC | GGCTTCGGATCTCTTCTCCT |
| 1982 | scaffold3778 | TGACTAATGAGGGAGGGGTG | CTCCTCGTCTGAGAACGGAC |
| 1983 | scaffold3513 | TGCACCACTCCCTGTAATCA | GTCTCTTGCTCAGGTTTGCC |
| 1984 | scaffold2823 | TGCATCACGTAGTCCCTTGA | TGCTTACAGTCACAGCCAGG |
| 1985 | scaffold58198 | TGCTCACGGATCTACAGTCG | CCAAAGTTTACTGCGGTGGT |
| 1986 | scaffold13776 | TGGTACCTACCACCGTCTCC | CCGGTTTGGTTTCAGTGAGT |
| 1987 | scaffold64134 | TGTTCACCGCATGAATGAAA | ACAATCAGATGGAAACCCCA |
| 1988 | scaffold17119 | TGTTCCTAAACCGCAACTCC | AACGCTGCTTCCTTCACAAT |
| 1989 | scaffold8295 | TGTTGGATTAAATCATGAGCCA | TCTTTGGGGAGATTTGTTGC |
| 1990 | scaffold33876 | TTAAGCCAAGCTTCCCAAGA | GGCTGTTGCGTATTACCGTT |
| 1991 | scaffold19944 | TTCATGGAGATTGGAGATTGG | CCCACCAGATGACTCAAACC |
| 1992 | scaffold1814 | TTCATTAATGGCGACGATCA | TCAGTGCTACGGTTGCTACG |
| 1993 | scaffold18450 | TTCTGTCTCTCTCCTCTCCTTCA | CGTCCGCAAGGACTATTCTC |
| 1994 | scaffold13465 | TTGATCTTCGGCATCATTCA | CATTGCTCCCACCAATCTCT |
| 1995 | scaffold10302 | TTTATCTGTAGCCCGTGGGA | CTCTCCCTATCCAGTCGGTG |
| 1996 | scaffold5839 | TTTCTCAGCACAGTTGACGG | AGCGAGCAACGTAGCCTTTA |
| 1997 | scaffold2388 | TTTGGGGTAAGCTCATGACA | TTTTACACTTATGGGTTTGATCG |
| 1998 | scaffold9523 | AAAACGAGTCAGACCCCAGA | TCACCTCGTGGGATTCTTTC |
| 1999 | scaffold6669 | AAACAGAAGACAAATTATGAAACGC | AACGGCTTCGAATGAAAGAA |
| 2000 | scaffold23662 | AAACATGAAGGGCCAAACAC | CGTCATGGCTCATGCAAGTA |
| 2001 | scaffold14068 | AAAGGAGAGGCCATCGATTC | TCGGTTTATGGGCTCTGAAG |
| 2002 | scaffold36599 | AACAACGGAAGCCTTTGAGA | TAAACCGGATATGCTCAGCC |
| 2003 | scaffold11771 | AACGATCTCGGTGGTGGTAG | AGGGACTCAGTGCTTCCTCA |
| 2004 | scaffold9302 | AACGCGGAACAGAGAGAGAG | AGAGTAAAGTGTGAGCCGCC |
| 2005 | scaffold34495 | AAGAAGGAGAGCGCACAAAA | AACTGGTGGGTGGTAATGGA |
| 2006 | scaffold10475 | AAGATGTCGCCGGAGTAAGA | TTTGCCATTGCCATACACAT |
| 2007 | scaffold1557 | AAGCTTCTGACTCCGGTCAA | TTCTCTCCATCGGTCTTCGT |
| 2008 | scaffold9211 | AAGCTTCTTCAGCCTCCTCC | TGTTGGCTCATTGTGGATGT |
| 2009 | scaffold930 | AAGGTAGGAGCCGGATTTGT | CATACTCTCTGCTGCCCACA |
| 2010 | scaffold18556 | AATGATCAGGAGGAACGTGG | ATCCCAAAACCCTAAATGGC |
| 2011 | scaffold22677 | AATGCCCCTAGGCTGAGATT | GAACGAACCAGACACCAACA |
| 2012 | scaffold24878 | AATGTCCCACACCTTCTTGC | CTGTCGTCGGAGTTCCATTT |
| 2013 | scaffold2567 | ACAAGTACCGGCGTCTATGG | AGCGATCCCTCCTCTCTCTC |
| 2014 | scaffold44277 | ACAGCTCCGTCGCTTTATCT | CATTTGGATCCATACGAGCC |
| 2015 | scaffold15569 | ACAGCTTTTGAGGTGTGGCT | CAGAGGGAAGCCAGGTAATG |
| 2016 | scaffold8780 | ACAGGAAGTTGCTACGCCAA | TTTTGAAGCCGATCAGTGTG |
| 2017 | scaffold24312 | ACCTGCAAAATGAATGAGCA | TTTGCAATTTCACAGGACCA |
| 2018 | scaffold24682 | ACGTCATGAAAGGTTTTGGG | AAACTCCACCCACCTCTCCT |
| 2019 | scaffold22828 | ACGTGGAGGCGTCTTATGTC | TTCTAAAGCACCTTACTTCTTTTTCA |
| 2020 | scaffold21246 | ACGTGGGACGTAGAAAGGTG | ATTCGCTCAAGCTTCCAAAA |
| 2021 | scaffold39869 | ACGTGTCCACGATAGGCATT | CTGTGGGCTCATGAATTGTG |
| 2022 | scaffold667 | ACTCAACCCCTCCCTTCAGT | CTTCCGCCTCACAATCGTAT |
| 2023 | scaffold8277 | ACTCTTATTCCACGGCATCG | AAGCTTAAACTTGCCACCGA |
| 2024 | scaffold3082 | ACTTCGCAGCACATCATCAG | CGCATTGGTCCATGTCTAAC |
| 2025 | scaffold24105 | AGAAAATTTCGCAGAACCCA | GCGATGAGAGGTGTCGATTT |
| 2026 | scaffold20539 | AGAAGTGGAAGCAAATGGATG | TCAGTGCATGATGAAGGCAT |
| 2027 | scaffold39891 | AGACCTTCGTAAAGCCCCTC | CGTGATGCTGTTCTGCTTGT |
| 2028 | scaffold15710 | AGAGACAGAAATGTGCGAAGC | GCTTGTTCAGGCGTTCATTT |
| 2029 | scaffold12896 | AGAGATTGCACACTCATGCTG | CCGATATCCGGATTGAACAC |
| 2030 | scaffold20474 | AGAGGGAGGATCAATGTGGA | CCCTCTCCATGCACTTGTCT |
| 2031 | scaffold20091 | AGCCAAGCTCCACAAGAAAA | CAGCTGAATTCCCCACAACT |
| 2032 | scaffold27173 | AGCCCATTCCTTTTCTTCGT | TTCCTCGGTTTTCCACTCAC |
| 2033 | scaffold24445 | AGCCTGTGGCTCTGAATTGT | TATTGCAATTTCTGCCGCTT |
| 2034 | scaffold10675 | AGCGACCTTTGGTGCTTCTA | AACCACAACTTTGACGGAGG |
| 2035 | scaffold4728 | AGGAGAGGGTGACGAGAGGT | GTCGAGCCGAAGAGACAATC |
| 2036 | scaffold24486 | AGGCTGAGACAAAGACTGGG | TTTCTTCTGCTTGATTCGGC |
| 2037 | scaffold2767 | AGGTTGTCGTGCCATACCTC | CCTGTACAATAGCCGCCTTG |
| 2038 | scaffold12935 | ATATGCCTTCGCAGTAACCG | AGAAAGGCGACGCTTCAATA |
| 2039 | scaffold48598 | ATCCTCAAGTCTCGCTCCAA | TAGCGACGATGGTAAGAGCA |
| 2040 | scaffold42007 | ATGTGGTAAGATTCGCTCCG | TTCTTTGGCTGCCTTCAACT |
| 2041 | scaffold28895 | ATTATCGAGTTCGGTGACGG | CTCTGGTTAGTCCCCTTCCC |
| 2042 | scaffold24605 | ATTGCAGCTTTTCATCCACA | CTCCTCTCGAAATCTGCGTC |
| 2043 | scaffold8855 | ATTTCCCGAACAGAACAACG | CCAAACCACCAATTCAACCT |
| 2044 | scaffold40783 | CAACCACGTGACACCGTTAG | TAAGCCAGAGCAACCGAAGT |
| 2045 | scaffold15830 | CAAGAGGCAAGGGAGATGAG | TAGGAAACCCTAGCCGTCAA |
| 2046 | scaffold37926 | CACAAGATTTTGACTGAATTTCCA | TCCCTCTTACGTGTTGCAGA |
| 2047 | scaffold34816 | CACCAACACCTGCTCTCAAA | GGGATGGGAAGAAGGAAAAG |
| 2048 | scaffold23071 | CAGAAAAGCTTTCAGCCACC | GGAACGGCTGGTTTGATTTA |
| 2049 | scaffold41100 | CAGTGCGAGGTACTGAAGCA | ATCTCGCTCAGACCAGAAGC |
| 2050 | scaffold4793 | CATCAACCTTCTTTAGCCGC | AAGGAGCAGGGAAGGAGAAG |
| 2051 | scaffold17072 | CATCCCCACTTCTTTCTCCA | GCTCAAGTGAGCTTTCTCCC |
| 2052 | scaffold33068 | CATGCTTGCCTGAAAAGACA | CCTTGTACTGCTCCTCTGCC |
| 2053 | scaffold31377 | CATTGTAGCGGTTACCGAAAA | TCGATAAGAAGCGAAGGGTG |
| 2054 | scaffold12861 | CCAAAGGCCACATTTGTTTT | CGATTCTCAGCTGTGGTGAA |
| 2055 | scaffold11099 | CCACCAGCATGAATTCAAAGA | GTCCTCATAATGCATCGCCT |
| 2056 | scaffold8071 | CCAGTTGCGTCTTTCAGTCA | GGTTTCCTTTTCTCCAAGCC |
| 2057 | scaffold59155 | CCATTTGAGTGGACTGGACC | TGAGAGAGCAGAGGAGACGA |
| 2058 | scaffold1269 | CCATTTTGCCTTCTCCTTGA | TATCGGAAGGACTCTGTGGC |
| 2059 | scaffold1623 | CCCAAGGAAATGCTTCAAAA | TGCATGCATCCACCTTGTAT |
| 2060 | scaffold11933 | CCCATCCAAGGAAATACGAA | CCCTTTGTGTATTTTGGCGT |
| 2061 | scaffold9574 | CCCATTCCATCAAAAGCACT | ATCGATTGGACAAAACTGGC |
| 2062 | scaffold45679 | CCCCTCAATCTCCACTTTCA | CGACATTCGGTTCTCCAGAT |
| 2063 | scaffold3314 | CCCGGAGATAAATCAGACGA | GGTGACGAAACCAAAGGAAA |
| 2064 | scaffold14227 | CCGACATCGGAAGATTAGGA | CCAACCTATTACTGGCGGAA |
| 2065 | scaffold4053 | CCGACTTTGATTGGTTTGGT | CCCTGCAAATTCGAGAAGAC |
| 2066 | scaffold7802 | CCGCTCCCTGTTTACAATCT | ACCTCCACCGTGATCTCAAC |
| 2067 | scaffold27225 | CCTACAGCAGTAACGGCTCC | AATGACAGGTCCCCTCCTCT |
| 2068 | scaffold27256 | CCTCGCAACCGTAAAAAGAA | CTTCCTCAACCCATCTCCAA |
| 2069 | scaffold23186 | CCTGCTGAAACTGTTGATGC | CGCCTCCGTGAGAGATTTAG |
| 2070 | scaffold38100 | CCTTGCGTCTGAAACATCAA | TTACCGGGAGTAAATGCAGC |
| 2071 | scaffold10837 | CCTTTATTCCATTTCCGGGT | TGGACCAGCCAAGGTTAGTT |
| 2072 | scaffold3474 | CGAATGGAGAAATAAGGGCA | TTACCTCATCAGGTGGAGGC |
| 2073 | scaffold41945 | CGAATTGCACATCCCTTCTT | GGAAGCTGATTGGATCCTCA |
| 2074 | scaffold11759 | CGACACGGAGCTTTAAGGAG | CGCGTCATCTACGCAAACTA |
| 2075 | scaffold25625 | CGACAGCTGCATGAGGATT | TCAGGAGATGGCTGTGAAGA |
| 2076 | scaffold26226 | CGAGGAAGTGATCGGAAGAG | ATCCAGAACAACACATGGCA |
| 2077 | scaffold20956 | CGAGGGGAGACTTGACAAAA | CGGTTTGAAGATGTTTGGGT |
| 2078 | C18534736 | CGCAAACAATTTCCTTACGC | TCGGATCAAATCATGGAGGT |
| 2079 | scaffold8317 | CGCTCACGAAAACTCCTTTC | TAACTAACATCCCAAGGCCG |
| 2080 | scaffold23090 | CGCTTGAGGTCGAGAGAGAG | AAGCAGCTACGTTGCTCACA |
| 2081 | scaffold44107 | CGTGTTGATCGGTGTTGAAA | CGTATACGTCTCGTTGGCAT |
| 2082 | scaffold4521 | CGTTAGCACAAAGACCGGAT | TGTGTTATCGTCGGGTTTGA |
| 2083 | scaffold3758 | CTAACAAGCAATCAACCGCA | AGCTCTTCGACGGATTCAAA |
| 2084 | scaffold10858 | CTAGAAGCTCCGGTGAATCG | GTGTTTAAACCCTCCGACGA |
| 2085 | scaffold122 | CTATAACCGAGGAGGGAGGG | CGGTGTCAAACCAACACCTA |
| 2086 | scaffold14020 | CTCCCCCAAGTGTGTGTTTC | CCATGTTCACAAATCCAACG |
| 2087 | scaffold11645 | CTCGAGAAGGTCGGTGAGAG | CCAACGGCTGAGATTAGAGC |
| 2088 | scaffold62274 | CTCGTTCTCGTAGAGCGTCC | AAGAGCTTACGGAGTGGCAA |
| 2089 | scaffold11646 | CTCTACAGCCACCTTCTCCG | TCCCTTCTCTCTCCTCACCA |
| 2090 | scaffold1184 | CTGATCGGCCAAGCAGTTAT | GTTCTTTCCACTGGCGACAT |
| 2091 | scaffold8563 | CTGCCTATAGGTCGATGCGT | TTCTGCGATTCTCGGAAACT |
| 2092 | scaffold26961 | CTGGGTTCGAGATGGAGAGA | AACTTAATGCGCCGTTTGAC |
| 2093 | scaffold49505 | CTTCTTGCTTTGGCCTTTTG | GTGTTTGCGTGTTGGACATC |
| 2094 | scaffold24135 | GAAACCCTAAAACCCCCAGA | GGAACGAAAAGCAATCGAAA |
| 2095 | scaffold8313 | GAAGCTGCTCTCCTTGTTCG | CACGATCAAACTCTATGCTGAAA |
| 2096 | scaffold19299 | GAAGTCCGCAACTCGCTAAG | TACATGCAACGCGCTATTGT |
| 2097 | scaffold18661 | GACTGAGGCAACACCTGTGA | ATGCTGAGTTCGATCCCAAA |
| 2098 | scaffold29812 | GAGAATCCAATCCGGACAGA | CACGTTCAGGTTCCCTGATT |
| 2099 | scaffold42189 | GAGCAGCAGAACCAAAGGAC | ATCATGACGATGACAAGCCA |
| 2100 | scaffold9073 | GAGCCTCGTCACTCACACAA | CTCTCGCATGTCGATTCTCA |
| 2101 | scaffold6890 | GAGGTTTCCACCGAGACAAA | CGTGTTCGCTCTTGGTTACA |
| 2102 | scaffold2271 | GAGTCATCGCAAGGCTCTTC | TCACCCACCCACCTATCCTA |
| 2103 | scaffold48395 | GAGTGGATGACGCGGTAAAG | ATGCAAATTTTTGCCCAGTT |
| 2104 | scaffold11867 | GATACACTCCGGGTCTGGAA | GCAAAAGAGAGGCACGTTTC |
| 2105 | scaffold27772 | GATGGATCGAGATGAAGGGA | CTTAACCGAATCGAACCGAA |
| 2106 | scaffold5594 | GCAAACCAAGTGGGAGATGT | ACGCTCTTTTTGCATGCTCT |
| 2107 | scaffold5227 | GCATCTGCTGGGAGATCTTT | AATTCGCTGAAAAGGCCTAA |
| 2108 | scaffold8882 | GCCATTGTCTTGTCTGCTCA | AAGCCCAGCCCATTAGTTCT |
| 2109 | scaffold62934 | GCCATTTGGTCTTCCACACT | TCTCTTCCTCCACGCTAAGG |
| 2110 | scaffold49000 | GCCTCTCAGGAGAACCCTCT | GTGCATTGGCACATTACTGG |
| 2111 | scaffold58365 | GCGCAAGGTAAAAACAATCA | CTGTGATAGACCCCTCCATGA |
| 2112 | scaffold16929 | GCGCCATACACTTCACCATA | CGACCCACGTACTTTCGTTT |
| 2113 | scaffold15627 | GCGGGATGGACTACAGGTTA | ACTGGAGCTACATAACGCCG |
| 2114 | scaffold48796 | GCGTCTTGGTCTCGTTCTTC | TGAGTTGTCACCAAACCCAA |
| 2115 | scaffold38895 | GCGTGACTCACAGTTCCTCA | ATTGCGGAGAAAGTGTACGG |
| 2116 | scaffold11050 | GCTCCTGAAGAGAGAGCGAA | TACCGCGTAAACCCTCACAT |
| 2117 | scaffold8584 | GCTCGTTGTTCAGCTCCTTC | CCATGATGCGTCTGGAGTAA |
| 2118 | scaffold11071 | GGACGTTCATGTCCCTTCAT | TCTTCGTTCATCGTCTGCAA |
| 2119 | scaffold14337 | GGATTTGAAACAAGGCAGGA | CGTGAAATCTCGACCCCTAA |
| 2120 | scaffold31961 | GGCACGTGAGTATGGAAGGT | GTCATGCATGCTGGTGAAAC |
| 2121 | scaffold27803 | GGCCCAAAACAAAAGAACAA | GAAGACGCGCATTTTCTAGG |
| 2122 | scaffold10499 | GGGATCAAAAAGAAAAACCCA | CATTTGGCTGCTGAAACTCA |
| 2123 | scaffold8150 | GGGCTTGAATGAGAAGTGGA | AAACGCCAGGATACGTGAAC |
| 2124 | scaffold12417 | GGTGAATGAGAAAACGGTGG | ATTCTCTCCACATTGCACCC |
| 2125 | scaffold13036 | GTATGGACCGGTTATGGACC | GGAGGTCTTTGGCCTTCTCT |
| 2126 | scaffold1397 | GTGTTTTGTCCGGATCTGCT | ACGTCATCAAACGAACTCCC |
| 2127 | scaffold3695 | GTTCCCATTTCTTTCCCGAT | GCTGGATCGAAAGAAAGCAC |
| 2128 | scaffold51190 | GTTCGCAGAGGAGAAGGATG | CGCCAAGCAGCTTCTTTAAT |
| 2129 | scaffold1358 | GTTCTCATTCGTCTTGGGGA | CGTGAGGTCATCTCCATCCT |
| 2130 | scaffold6355 | GTTTGCGCCCAATAGTGTTT | TTTGCGAATCTGTACTTGTCG |
| 2131 | scaffold50471 | GTTTGTCAGGGAACATGGCT | CTGCCACGTATTCGTCAAGA |
| 2132 | scaffold12027 | TAAAAGCTTCCGCTTTTTGC | GGGCGAGGTTCTTAAGGTTT |
| 2133 | scaffold64592 | TACCCTTTCACCCTCAGCAG | CCTCCGTCCTCATTATGGAA |
| 2134 | scaffold12033 | TATTGGGGGATGTTAACGGA | TGGTGAAGAAGGGTTCTTGG |
| 2135 | scaffold7406 | TCAATTGGGCGTATTCATCA | CGTGAAAAATGTCAACAATCG |
| 2136 | scaffold63998 | TCACAAGCAACGAGAACTGC | CTGGCTTGCTTCACTTTCCT |
| 2137 | scaffold13589 | TCCAAGCTGAGGAGGAAGAA | CTGCAGACATCAATTCCCCT |
| 2138 | C18470329 | TCCACGCAGGAGAAGCTAAT | CTCGCATACAAACAAACCGA |
| 2139 | scaffold51608 | TCCGACCACGTGAAACATTA | TAATCGACTTTTCCCGATGC |
| 2140 | scaffold40857 | TCCTTGCAGCCTTCCTCTTA | CTGGCCGTGGATAGATCAGT |
| 2141 | scaffold31510 | TCGATCAACAGGACCTGACA | ATAGTCCAGAGGTTGCTCCG |
| 2142 | scaffold12008 | TCGCGACCAAAATTCAAAGT | GGTTTCCATTGGTGTTGTCC |
| 2143 | scaffold6145 | TCGTCTGGAGGAAACAACAA | AAGCGTACGTGGACCATTTC |
| 2144 | scaffold673 | TCTGATCAACGAGATCGCAG | GCAACCCAGTACCCCCTATT |
| 2145 | scaffold15562 | TCTGCTTAAAATCCCCATCG | CGGAAGAGAGAAACACCAGC |
| 2146 | scaffold42050 | TCTTATCGCTGAGGAATGGG | GGTGGGGTTCTTATCGGAAT |
| 2147 | scaffold46974 | TCTTCGATCAGAGTCGCCTT | CTGGGTGGCTCTCTTAGACG |
| 2148 | scaffold22364 | TCTTTCCCATCGAATCATCC | GATACGGTTGACGTCGGAAG |
| 2149 | scaffold6174 | TGACATCAGCTCCACTACTACAGA | GTGAAGCTGTGGCGTATGAA |
| 2150 | scaffold31157 | TGATGGATTCATGACTGGGA | GGAGGCAGACCAAGTTTCAG |
| 2151 | scaffold5141 | TGATTTGTCAATCCCAGGGT | CGAAAGTGGAAACAACCTCG |
| 2152 | scaffold11927 | TGCATTTTGGAAAATTAAGCA | TGCATCATACAAGATTTGGACA |
| 2153 | scaffold17685 | TGGACACAGCTGGCAATAAA | CACTGGGCCTTCTGCTACTC |
| 2154 | scaffold15390 | TGGACATGTGGAAGGCTTTT | CACAACCCACCTCTCTCTCC |
| 2155 | scaffold13693 | TGGCGAGCAGAGAAGTTGTA | CTTACTTGGGCGGTGTGATT |
| 2156 | scaffold3403 | TTAGGCTCGGTTGCAGAAGT | TAACGCCCATGTCAACGATA |
| 2157 | scaffold6720 | TTCAATCGCCATTGTTCAAA | TTGGGAGGATAAGAACGACG |
| 2158 | scaffold17020 | TTCTCTTCTCAAACAACATGCAA | ATCCCTAGCAAGCAACCCTT |
| 2159 | scaffold17328 | TTCTTCCTCCGTTGGTATCG | ACTTCCCCAGCTTTTCTTCC |
| 2160 | scaffold23682 | TTCTTGGTGTGTCCTGCAAA | GAGCTATGGCAATCCTCCTG |
| 2161 | scaffold23809 | TTGAAAGTCGACGAGAGGCT | GCCGAACTGCAAATTTAAGC |
| 2162 | scaffold25426 | TTGACTATGACCCTCTGTTTTGAA | CCTTCACCCTACCAAAACCA |
| 2163 | scaffold4963 | TTGGAAGATAGGTGCGGTAAA | GGAGACATCACTCCCTTGGA |
| 2164 | scaffold24851 | TTGGGGACAGAGAAGGATTG | CTGTAGATAGAATCCGCGCC |
| 2165 | scaffold10201 | TTGGTATTAACCATTGCAACCTT | CCAAGCATTTGGTTTACACG |
| 2166 | scaffold13942 | TTGTGACTGTTACCTCGGCA | TATGCATATGGGTTGCTTCG |
| 2167 | scaffold15283 | TTTAAGGAAGCTGACCGTGG | GCTGAAGACTTTGGAGCAGC |
| 2168 | scaffold27871 | TTTATACTCGCCCCAAGCTC | TCGAAAACAACGTCGAAACA |
| 2169 | scaffold17948 | TTTGATACGATTTCACCGCA | AGGTATGACAGGCCCAAATG |
| 2170 | scaffold50823 | TTTGCAGGACTGAGCCTTTT | AATCATCATCGCCGAAACTC |

**Initial gels/blots are used in Figure 2**

**
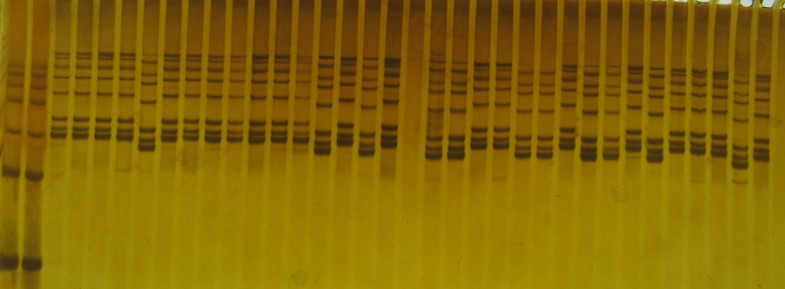

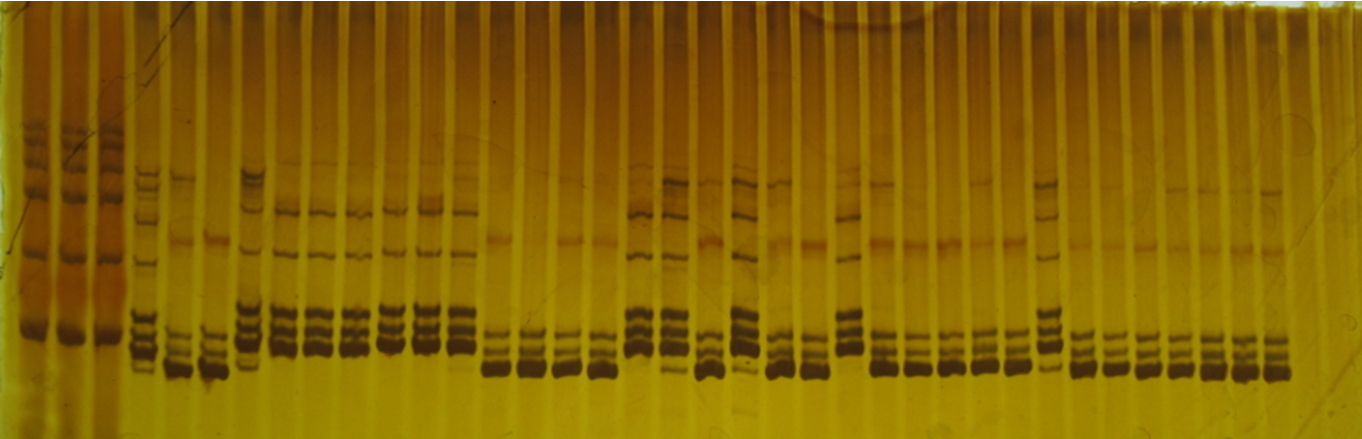

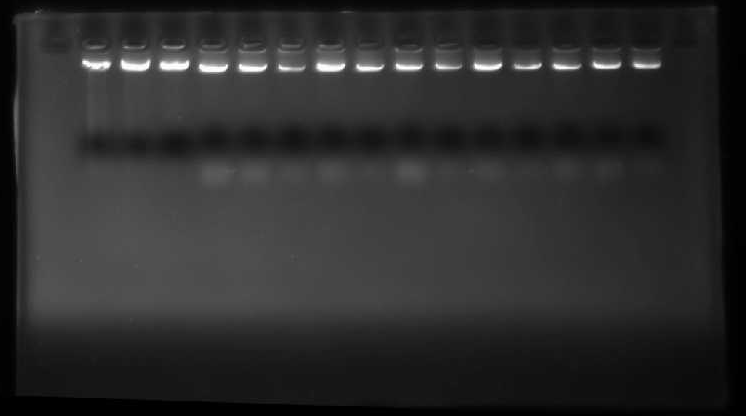
**
